# Precision nutrition to reset virus-induced human metabolic reprogramming and dysregulation (HMRD) in long-COVID

**DOI:** 10.1038/s41538-024-00261-2

**Published:** 2024-03-30

**Authors:** A. Satyanarayan Naidu, Chin-Kun Wang, Pingfan Rao, Fabrizio Mancini, Roger A. Clemens, Aman Wirakartakusumah, Hui-Fang Chiu, Chi-Hua Yen, Sebastiano Porretta, Issac Mathai, Sreus A. G. Naidu

**Affiliations:** 1 Global Nutrition Healthcare Council (GNHC) Mission-COVID, Yorba Linda, CA USA; 2 N-terminus Research Laboratory, 232659 Via del Rio, Yorba Linda, CA 92887 USA; 3 School of Nutrition, Chung Shan Medical University, 110, Section 1, Jianguo North Road, Taichung, 40201 Taiwan; 4 College of Food and Bioengineering, Fujian Polytechnic Normal University, No.1, Campus New Village, Longjiang Street, Fuqing City, Fujian China; 5 President-Emeritus, Parker University, 2540 Walnut Hill Lane, Dallas, TX 75229 USA; 6 University of Southern California, Alfred E. Mann School of Pharmacy/D. K. Kim International Center for Regulatory & Quality Sciences, 1540 Alcazar St., CHP 140, Los Angeles, CA 90089 USA; 7 International Union of Food Science and Technology (IUFoST), Guelph, ON Canada; 8 IPMI International Business School Jakarta; South East Asian Food and Agriculture Science and Technology, IPB University, Bogor, Indonesia; 9 Department of Chinese Medicine, Taichung Hospital, Ministry of Health & Well-being, Taichung, Taiwan; 10 Department of Family and Community Medicine, Chung Shan Medical University Hospital; School of Medicine, Chung Shan Medical University, Taichung, Taiwan; 11 President, Italian Association of Food Technology (AITA), Milan, Italy; 12 Experimental Station for the Food Preserving Industry, Department of Consumer Science, Viale Tanara 31/a, I-43121 Parma, Italy; 13 Soukya International Holistic Health Center, Whitefield, Bengaluru, India

**Keywords:** Nutrition, Mechanisms of disease

## Abstract

SARS‐CoV‐2, the etiological agent of COVID-19, is devoid of any metabolic capacity; therefore, it is critical for the viral pathogen to hijack host cellular metabolic machinery for its replication and propagation. This single-stranded RNA virus with a 29.9 kb genome encodes 14 *open reading frames* (ORFs) and initiates a plethora of virus–host protein–protein interactions in the human body. These extensive viral protein interactions with host-specific cellular targets could trigger severe *human metabolic reprogramming/dysregulation* (HMRD), a rewiring of sugar-, amino acid-, lipid-, and nucleotide-metabolism(s), as well as altered or impaired bioenergetics, immune dysfunction, and redox imbalance in the body. In the infectious process, the viral pathogen hijacks two major human receptors, *angiotensin-converting enzyme* (ACE)-2 and/or *neuropilin* (NRP)-1, for initial adhesion to cell surface; then utilizes two major host proteases, TMPRSS2 and/or furin, to gain cellular entry; and finally employs an endosomal enzyme, *cathepsin L* (CTSL) for fusogenic release of its viral genome. The virus-induced HMRD results in 5 possible infectious outcomes: asymptomatic, mild, moderate, severe to fatal episodes; while the symptomatic acute COVID-19 condition could manifest into 3 clinical phases: (i) hypoxia and hypoxemia (Warburg effect), (ii) hyperferritinemia (‘cytokine storm’), and (iii) thrombocytosis (coagulopathy). The mean incubation period for COVID-19 onset was estimated to be 5.1 days, and most cases develop symptoms after 14 days. The mean viral clearance times were 24, 30, and 39 days for acute, severe, and ICU-admitted COVID-19 patients, respectively. However, about 25–70% of virus-free COVID-19 survivors continue to sustain virus-induced HMRD and exhibit a wide range of symptoms that are persistent, exacerbated, or new ‘onset’ clinical incidents, collectively termed as *post-acute sequelae of COVID-19* (PASC) or long COVID. PASC patients experience several debilitating clinical condition(s) with >200 different and overlapping symptoms that may last for weeks to months. Chronic PASC is a cumulative outcome of at least 10 different HMRD-related pathophysiological mechanisms involving both virus-derived virulence factors and a multitude of innate host responses. Based on HMRD and virus-free clinical impairments of different human organs/systems, PASC patients can be categorized into 4 different clusters or sub-phenotypes: *sub-phenotype-1* (33.8%) with cardiac and renal manifestations; *sub-phenotype-2* (32.8%) with respiratory, sleep and anxiety disorders; *sub-phenotype-3* (23.4%) with skeleto-muscular and nervous disorders; and *sub-phenotype-4* (10.1%) with digestive and pulmonary dysfunctions. This narrative review elucidates the effects of viral hijack on host cellular machinery during SARS-CoV-2 infection, ensuing detrimental effect(s) of virus-induced HMRD on human metabolism, consequential symptomatic clinical implications, and damage to multiple organ systems; as well as chronic pathophysiological sequelae in virus-free PASC patients. We have also provided a few evidence-based, human *randomized controlled trial* (RCT)-tested, precision nutrients to reset HMRD for health recovery of PASC patients.

## Introduction

*Severe Acute Respiratory Syndrome Coronavirus 2* (SARS-CoV-2), an enveloped, positive-sense, single-stranded RNA virus, is the etiological agent of *Coronavirus Disease 2019* (COVID-19)^1^. The *World Health Organization* (W.H.O.) estimates that after recovery from acute phase of SARS-CoV-2 infection, around a quarter of such population experience persistent or new-onset symptoms in long-term referred to as ‘*post-acute sequalae of COVID*’ (PASC) or long COVID^2^. Accordingly, more than 173 million individuals around the world have PASC, based on a conservative estimated incidence of 25% of infected people and over 692 million documented COVID-19 cases globally^3–5^. The transition of post-COVID patients (after recovery from acute SARS-CoV-2 infection) to a virus-free disease state with lingering/chronic clinical manifestations, has emerged as a new global health crisis—the long-COVID.

PASC could encompass several adverse clinical impairments that may trigger chronic metabolic dysfunctions involving *cardiovascular* (CV), *central/peripheral nervous* (CNS/PNS), *gastrointestinal* (GI), pulmonary, reproductive, skeleto-muscular, and endocrinal systems^6,7^. New onset metabolic disorders also include *type 2 diabetes mellitus* (T2DM), *myalgic encephalomyelitis/chronic fatigue syndrome* (ME/CFS) and dysautonomia, especially the *postural orthostatic tachycardia syndrome* (POTS)^8–11^. PASC could inflict a plethora of long-term symptoms that may linger for years, while clinical manifestations of new onset ME/CFS and POTS may persist throughout lifespan^5,12,13^.

SARS-CoV-2, the newly emerged RNA (29.9-kb) virus possess a unique genomic ability to insert and ‘reprogram’ a mega-fold larger size human DNA (3.1-Mb) and its cellular metabolic machinery to prime, alter, and redirect host macro-molecules for its own life cycle. The SARS-CoV-2 genome interacts with a few thousands of human metabolites in a specific manner to facilitate its infectious process^14–16^. Furthermore, the virus particle categorically hijacks vital human factors for its cell surface binding, host invasion, and viral RNA integration with human DNA. Accordingly, SARS‐CoV‐2 genomic ‘reprogramming’ of human DNA and its host hijacking of vital cellular factors cumulatively results in metabolic ‘dysregulation’ in the body^17–20^. Interaction of SARS-CoV-2 proteins with specific host cell targets also rewire metabolic pathways, and alter or impair bioenergetics, immune response, and redox homeostasis in the human body, to favor the virus^21,22^. Thus, SARS-CoV-2 proteins could sense the host cellular metabolic status and trigger *human metabolic reprogramming/dysregulation* (HMRD) in the infected human host. Accordingly, the unique genomic map of SARS-CoV-2 virus and the extent of its mediated virus–host protein–protein interactions that trigger *human metabolic reprogramming and dysregulation* (HMRD) in favor of the virus life cycle, defines the ultimate severity and fate of COVID-19^23–26^.

In many COVID-19 cases, the virus-induced HMRD may not reset or revert even after patient discharge as virus-free (RT-PCR negative) survivors. Interestingly, several persistent clinical manifestation(s) of post-COVID symptoms in PASC patients, sustain as an aftermath from earlier SARS-CoV-2-mediated hijack of host cellular factors (i.e., ACE2, NRP1, furin, TMPRSS2, and CTSL); in tandem with other human factors such as the *human leukocyte antigen* (HLA), epigenetics, preexisting comorbidities (i.e., T2DM, CVD, obesity), age, preceding systemic impairments (i.e., hyperinflammation, micro-thrombosis, fibrosis, dysbiosis, autoimmunity, etc.), and socio-demographic factors (i.e., food security, environment, access to medical care)^22,27,28^.

Healthcare strategies to combat PASC, the novel virus-induced human metabolic syndrome, requires an in-depth understanding of the following: (i) the genomic and metabolomic (proteomic/lipidomic) signatures of SARS-CoV-2 and their interactions with host cellular metabolic machinery, (ii) the virus-induced HMRD and resulting pathophysiological manifestations in the onset and progression of COVID-19, (iii) the cumulative role of HMRD, symptomatic outcomes (disease spectrum), and comorbidities in systemic/multi-organ dysfunction during COVID-19, (iv) both virus- and host-mediated factors that contribute to transition of acute SARS-CoV-2 infection (COVID-19) into a persistent chronic state of virus-free PASC, (v) protracted effects of HMRD in tandem with patient’s history, in the development of ‘new onset’ metabolic syndromes (i.e., T2DM, CVD, ME/CFS and POTS) among PASC patients, (vi) stratification/categorization of PASC patients based on persistent symptoms, organ/system involvement, and metabolic dysfunction for specific target-delivered health recovery regimens, and (vii) the structure-function activity of specific bio-functional dietary compounds in formulating precision nutrition protocols to reset SARS-CoV-2-induced HMRD in chronic PASC.

This narrative review is an attempt to elaborate and consolidate our current understanding of the molecular mechanisms of SARS-CoV-2 infection, the detrimental effect(s) of this infectious process on human metabolism, consequential symptomatic clinical manifestations, and damage to multiple organ systems; as well as chronic pathophysiological sequelae in virus-free COVID-19 survivors – the long-COVID or PASC patients. We have also provided a few evidence-based, *randomized controlled trial* (RCT)-tested precision nutrients to reset virus-induced HMRD, a detrimental aftermath resulting from virus-hijacked host cellular metabolic machinery in post-COVID survivors, the affected long-COVID patients worldwide.

## SARS-CoV-2 infection: hijack of host cellular metabolic machinery

The SARS‐CoV‐2 obviously lacks metabolic enzymes, a critical requisite for viral genomic replication, protein synthesis, and lipogenesis. Therefore, the virus strategically hijacks host cellular metabolic machinery and re-directs free *amino acids* (AAs) and *fatty acids* (FAs), as building blocks for viral progeny and propagation. Accordingly, SARS‐CoV‐2 genome and its products reprogram and dysregulate human metabolism at transcription, translation, and *post-translational modification* (PTM) levels^17–20^. Interaction of SARS-CoV-2 proteins with specific host cellular targets could rewire sugar-, AA-, FA-, as well as nucleotide-metabolism(s), and distinctly alter or impair bioenergetics, immune response, and redox homeostasis in the human body, thereby facilitate viral life cycle^21,22^. SARS-CoV-2 proteins could sense the host cellular metabolic status and accordingly trigger *human metabolic reprogramming/dysregulation* (HMRD) in the infected human host.

### The viral genome

The SARS-CoV-2 genome is a 29.9-kb RNA that consists of 14 *open reading frames* (ORFs) encoding two large polyproteins (ORF1a and ORF1b) and 13 small ORFs that encode viral structural proteins and other polypeptides. Polyproteins from the large ORF1a/b are further arranged into 16 *non‐structural proteins* (*nsp1* to *nsp16*)^1,29^. The structural proteins comprise of *nucleocapsid* (N), *membrane* (M), *envelope* (E), and *spike* (S) proteins. The M and E proteins are located among the S-proteins in the viral envelope^30^. Based on the structural map of SARS-CoV-2, about 6% of the viral proteome mimics human proteins, while nearly 7% has been implicated in cellular hijacking mechanisms, and about 29% of proteome self-assembles into heteromeric components to support viral replication^31^.

### Virus–host interactome

Virus-human host protein–protein interactions play a major role in clinical outcomes of acute SARS-CoV-2 infection and its long-term sequelae, the PASC. A *ribonucleoprotein* (RNP) capture has identified a direct binding of SARS-CoV-2 RNA with 109 human host factors^32^. A comprehensive virus–host interactome of 29 viral (i.e., non-structural/structural) proteins, and 18 host/human cellular proteins (i.e., CSR, proteases, as well as restriction, replication, and trafficking molecules), showed an extensive involvement of >4780 unique high-confidence interactions of SARS-CoV-2 with human metabolome^14^. These diverse virus–host interactions could reprogram/dysregulate host cellular functions such as genomic, mitochondrial, lipidomic, and innate defense activities at various levels in human metabolism.

#### Viral infection reprograms host genomics

The SARS-CoV-2 protein, *nsp1*, binds to human ribosomes and inhibits host cellular translation^33^. The SARS-CoV-2 protein ORF3a interacts with host transcription factor ZNF579 and directly affects human gene transcription^34^. Viral ORF8 acts as a histone mimic and disrupts host cell epigenetic regulation^35^. Viral protein *nsp12 (RNA-dependent RNA polymerase)* could sense host nucleotide availability and modulate replication efficacy of the viral genome^36^. SARS-CoV-2 infection reprograms host folate and one-carbon metabolism at the PTM level to support de novo purine synthesis for replication of viral genome, through bypassing the viral shutoff of host translation^37^.

#### Viral infection reprograms cellular mitochondria

Interaction of viral gene *nsp6* with mitochondrial proteins (i.e., *ATP synthase*) alters cellular ATP synthesis^1^. Thus, SARS-CoV-2 infection dysregulates mitochondrial metabolism and forces the host cell to generate energy (ATP) and other metabolites to support viral life cycle^38^. Viral *nsp12* could alter AA metabolism (especially of the *branched‐chain amino acids*, BCAA), while *nsp12, nsp7*, and *nsp8* interactions with *electron transport chain* (ETC) and ribosomal proteins could potentially dysregulate mitochondrial respiration^39^.

#### Viral infection reprograms host lipid metabolism

Lipids play a major role in viral life cycle, accordingly the SARS-CoV-2 infection affects host lipidome by reprogramming cellular FA metabolism and nucleotide biosynthesis^40,41^. Viral protein *nsp7* could potentially alter host lipid metabolism, through its avid interaction with host enzymes involved in FA-β‐oxidation and lipogenesis^42^. SARS‐CoV‐2 up-regulates lipid biosynthesis to support the assembly of lipid bilayer‐envelope of virion particle^43,44^.

#### Viral infection impairs innate host defense

Viral ORF3a interacts with *heme oxygenase-*1 (HO-1) and reprograms heme metabolism leading to *iron (Fe)-redox dysregulation* (FeRD) during SARS‐CoV‐2 infection^27,45,46^. HO-1 is a stress-induced, anti-inflammatory, immune-modulatory, and cyto-protective enzyme that degrades heme into carbon monoxide, free iron, and biliverdin^47^, consequently, the virus-induced HMRD could compromise host innate and adaptive immune responses. Redox imbalance, FeRD in particular, results from virus-induced HMRD and represents a critical state both in the pathogenesis of SARS‐CoV‐2 infection and host inflammatory response^27,48,49^. Antioxidant enzymes such as *superoxide dismutase 1* (SOD1), and *glucose‐6‐phosphate dehydrogenase* (G6PD) decrease from HMRD-induced *oxidative stress* (OxS) and protein degradation^50,51^. Furthermore, viral protein *nsp5* and *nsp14* interact with host redox-enzymes: *glutathione peroxidase* (GPx) and *peroxiredoxin* (Prx), in both cytoplasm and mitochondria to dysregulate redox balance in different cellular compartments and enhance SARS-CoV-2 infection^21^. The viral protein encoded by ORF6 potently inhibits nuclear trafficking and helps viral evasion of IFN-mediated host defenses^52^. Viral protein, *nsp14*, interacts with the catalytic domain of Sirt1, dysregulates Nrf2/HO1 axis, and impairs host antioxidant defense^53^.

### Viral binding/attachment to human cell surface receptors (CSRs)

**Fig. 1:**
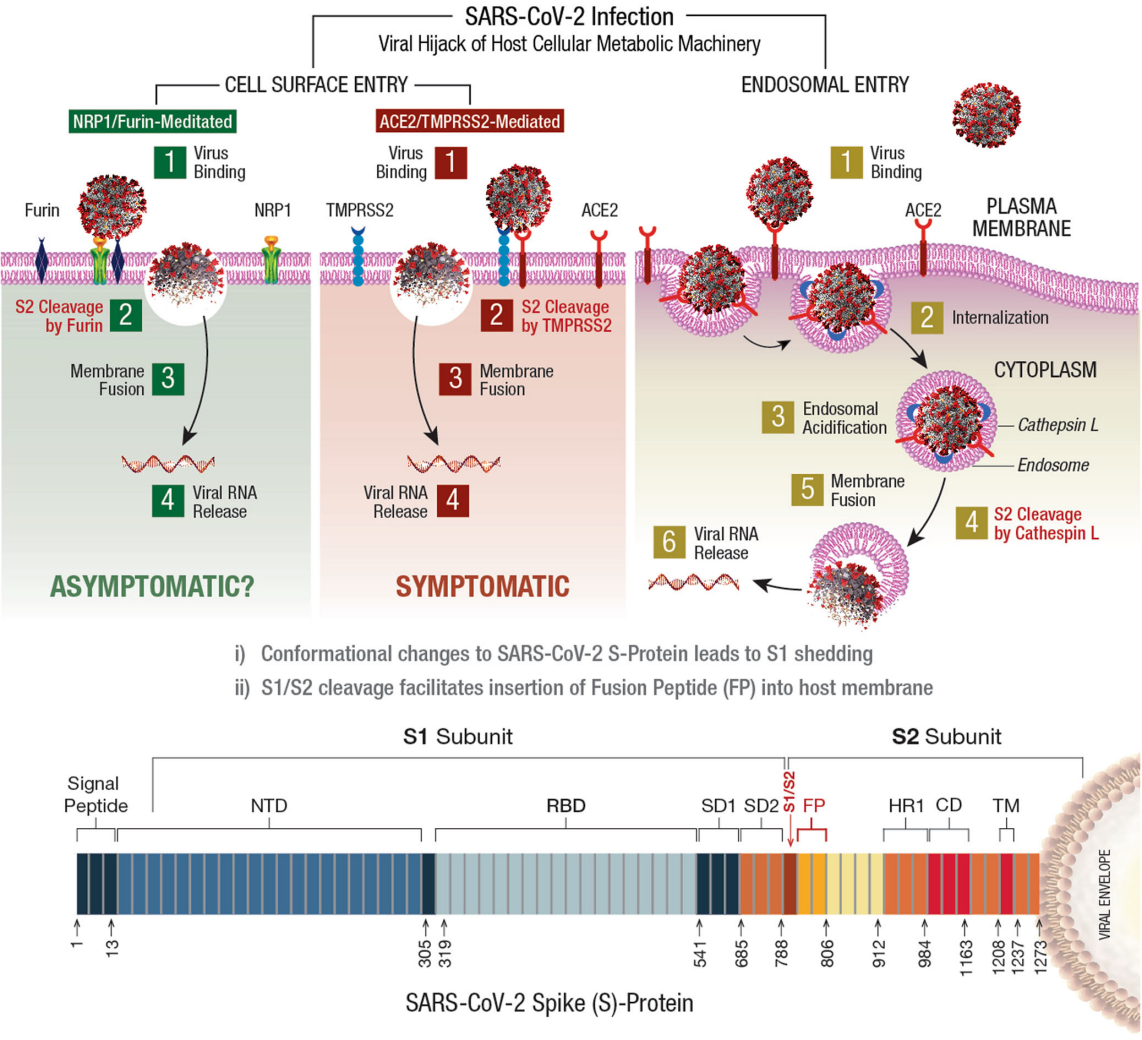
Viral hijack of host cellular metabolic machinery. SARS-CoV-2 infection of a susceptible host is achieved through viral *spike* (S)-protein-mediated hijack of human cell surface receptors (ACE2 and/or NRP1) and cell membrane proteases. The S1-region on viral S-protein contains a *receptor-binding domain* (RBD) that specifically recognizes host cell surface receptor(s) and exposes the S2 site^55^. For fusion with host cell membrane, the viral S-protein hijacks specific cellular proteases for activation (‘priming’) of viral S-protein at the S1/S2 region. Subsequent conformational changes to viral S-protein lead to S1 shedding by cleavage of S1/S2 fragments. This process facilitates insertion of *fusion peptide* (FP) into host membrane. Accordingly, proteolytic cleavage by cellular enzymes TMPRSS2 and/or furin accomplish the task of viral FP insertion into host cell membrane. Alternatively, SARS-CoV-2 could also hijack lysosomal protease *cathepsin L* (CTSL) for direct viral endocytosis, where the viral membrane fuses with luminal face of the endosomal membrane facilitating viral RNA transfer into the cytosol. Thus, SARS-CoV-2 could infect the human by hijacking these 5 major host cellular factors via different routes of entry and elicit a wide range of clinical outcomes. The *angiotensin-converting enzyme 2* (ACE2)/TMPRSS2-mediated viral infection and/or the ACE2/CTSL-mediated endosomal route may result in full-spectrum symptomatic COVID-19. The alternative *neuropilin 1* (*NRP1)/furin*-mediated route^62,63^ may down-regulate human pain receptors and manifest as asymptomatic to mild disease outcomes.

The virulent outcome of a SARS-CoV-2 infection depends on (i) binding/interaction of viral S-protein with human *cell surface receptors* (CSR) and (ii) priming of S-protein by human cellular proteases^54,55^. This infectious process is accomplished by viral hijack of cellular metabolic machinery through sequential steps of viral attachment, invasion, RNA replication, and propagation^56^. The viral S-protein serves as an anchor to interact with host tissue, followed by sequential cleavage of S-protein to facilitate viral entry^57,58^. The viral hijack of host cellular metabolic machinery during SARS-CoV-2 infection is depicted in Fig. 1

The SARS-COV-2 S-protein hijacks human *angiotensin-converting enzyme 2* (hACE2) to anchor on the host cell surface. The SARS-CoV-2 S/ACE2 complex undergoes conformational change for proteolytic priming/activation. The N-terminal S1 subunit contains *receptor-binding domain* (RBD) region, which avidly binds to the *carboxypeptidase* (CPD) domain on the hACE2 receptor and exposes the S2 site^55^. Co-expression of ACE2 with membrane serine proteases is high on ileal absorptive enterocytes in the GI tract, nasal goblet secretory cells and type II pneumocytes in the respiratory tract, as well as on the urogenital epithelia^59,60^.

SARS-CoV-2 may also infect host cells independent of the ACE2 receptor binding. The carbohydrate moieties on viral S-protein surface could facilitate viral internalization via innate immune factors, such as *neuropilin* (NRP)-1, *C-lectin type receptors* (CLR), and *toll-like receptors* (TLR), as well as the non-immune receptor *glucose-regulated protein 78* (GRP78) for systemic spread of infection^61^. NRP1, a transmembrane glycoprotein involved in *cardiovascular* (CV), neuronal, and immune regulation, is also hijacked by SARS-CoV-2 for host cell surface binding^62^. NRP1, widely expressed in olfactory and respiratory epithelia, is shown to enhance TMPRSS2-mediated viral cell entry^63^. NRP1 binds to S1 through a multi-basic *furin-cleavage site* (FCS) and promotes S1 shedding to expose the S2′ site for TMPRSS2 priming^64^.

The S-protein of SARS-CoV-2 has a polybasic insertion (PRRAR) region at the S1/S2 site, which is readily cleaved by furin enzyme^65^. *Furin cleavage site* (FCS) is an important determinant of SARS-CoV-2 transmission in the human population. After binding to ACE2 and/or NRP1 receptors, the S-protein is proteolytically pre-activated by human proprotein convertase *furin*^66^. High-affinity interaction of ACE2 and/or NRP1 with the RBD of viral S-protein, followed by cell-mediated furin pre-activation could effectively facilitate host cellular entry of SARS-CoV-2 while evading host immune surveillance^67^.

### Viral entry via host cell membrane fusion

The S-protein cleavage site, S1/S2 provides two sequential functions for successful viral entry. The RBD region on the S1 subunit recognizes anchor point(s) on the host cell surface, whereas the S2 subunit facilitates fusion of viral envelope with the host cell membrane after proteolytic cleavage of S1/S2 site to mediate viral entry^68,69^. Accordingly, SARS-CoV-2 hijacks several host proteases to enter human target cells and enhance its spread in the body. These proteases include cell surface *transmembrane protease/serine* (TMPRSS) proteases, *cathepsins*, furin, elastase, factor Xa, and trypsin^70^.

The S-protein harbors an FCS between the S_1_/S_2_ subunits, processed during biogenesis that sets this novel viral pathogen apart from other SARS-related CoVs^71^. Furin cleavage exposes the S2 subunit for further processing by the host serine proteases for subsequent viral entry^72,73^. Furin impacts the cellular entry of SARS-CoV-2 in a unique manner by pre-activation of S2 subunit thereby reducing viral dependance on other human proteases for cellular entry^54^. After furin cleavage, the S2′ site requires an additional proteolytic step to facilitate the fusion of viral envelope with host cell membrane. This process involves two major human proteases: the TMPRSS2 in plasma membrane and *cathepsin-L* (CTSL) in the endo-lysosome^74^.

Human TMPRSS2, an enzyme widely expressed in several human cells, acts on the S2 prime (S2′) region, and cleaves the S-protein^75^. This proteolytic process results in structural rearrangement of S-protein and allows fusion between the viral envelope and host cell membrane^57,76^, which cumulatively drives an efficient internalization (infection) of SARS-CoV-2 into target host cells^55,77^. CTSL, a pH-dependent endo-lysosomal protease, cleaves the S-protein and facilitates viral fusion with the host endosomal membrane. Also, SARS-CoV-2 could induce cellular transcription, elevate CTSL activity, and increase viral infection^78^.

Distinct variabilities of infection rates, epidemiological transmission, and clinical outcomes during COVID-19 pandemic raises an intriguing question, whether the emergence of SARS-CoV-2 *variants of concern* (VOCs) with function-specific mutations in ACE2, furin, and TMPRSS2 expression has played any role in disease manifestations and *case fatality rates* (CFR)^55,75^. The estimated *reproduction number* (R_0_) of COVID-19 is around 3.28^1^. *R*_0_ represents viral transmissibility, indicating an average number of new infections transmitted by an infected individual in a totally naïve population. For *R*_0_ > 1, the number of infected cases is likely to increase, and for *R*_0_ < 1, viral transmission is likely to die out. From an inanimate transmission standpoint, SARS-CoV-2 has a decay rate of 10^3.5^ to 10^2.7^ median *tissue culture infectious dose* (TCID)_50_/L, like the decay rate of SARS-CoV (10^4.3^ to 10^3.5^ TCID_50_/mL), and the virus could remain infectious in aerosols for several hours and on surfaces for up to one day^79^.

## COVID-19: clinical manifestatons

The symptomatic progression of COVID-19 requires that a genetically competent (virulent) SARS-CoV-2 pathogen (i) infects a susceptible host via specific CSR, invades and internalizes into the cell utilizing host membrane proteases, (ii) induces HMRD to ensure ready access to an active host cellular metabolic machinery for an uninterrupted viral replication, (iii) inactivates innate host defense to evade viral elimination, and (iv) exits the infected host cell and repeats the viral propagation cycle for exponential growth and transmission^26^.

The viral load usually reaches its peak at symptomatic onset during the initial weeks of infection and is detectable by *reverse transcription polymerase chain reaction* (RT-PCR) within the first week of infection. An infected person is estimated to carry about 10^9^ to 10^11^ virions at the peak of infection^80^. Severe COVID-19 patients might shed viral particles for prolonged periods of up to 4 weeks after symptomatic onset^81^. SARS-CoV-2 RNA (RT-PCR positive) could be detected in the upper respiratory tract (nasopharyngeal for about 7–8 weeks, throat, and sputum for about 4–5 weeks)^82^. Multi-organ viral tropism, mainly localized across lungs, trachea, kidney, heart, or liver, predominantly in cells expressing ACE2, TMPRSS2, or both has been reported. Viral RNA has also been detected in tonsils, salivary glands, oropharynx, thyroid, adrenal gland, testicles, prostate, ovaries, small bowel, lymph nodes, skin and skeletal muscle^83^. SARS-CoV-2 kidney tropism with high viral load in urine sediments from COVID-19 patients (within 2 weeks) correlates with increased incidence of AKI and mortality^84^.

**Fig. 2:**
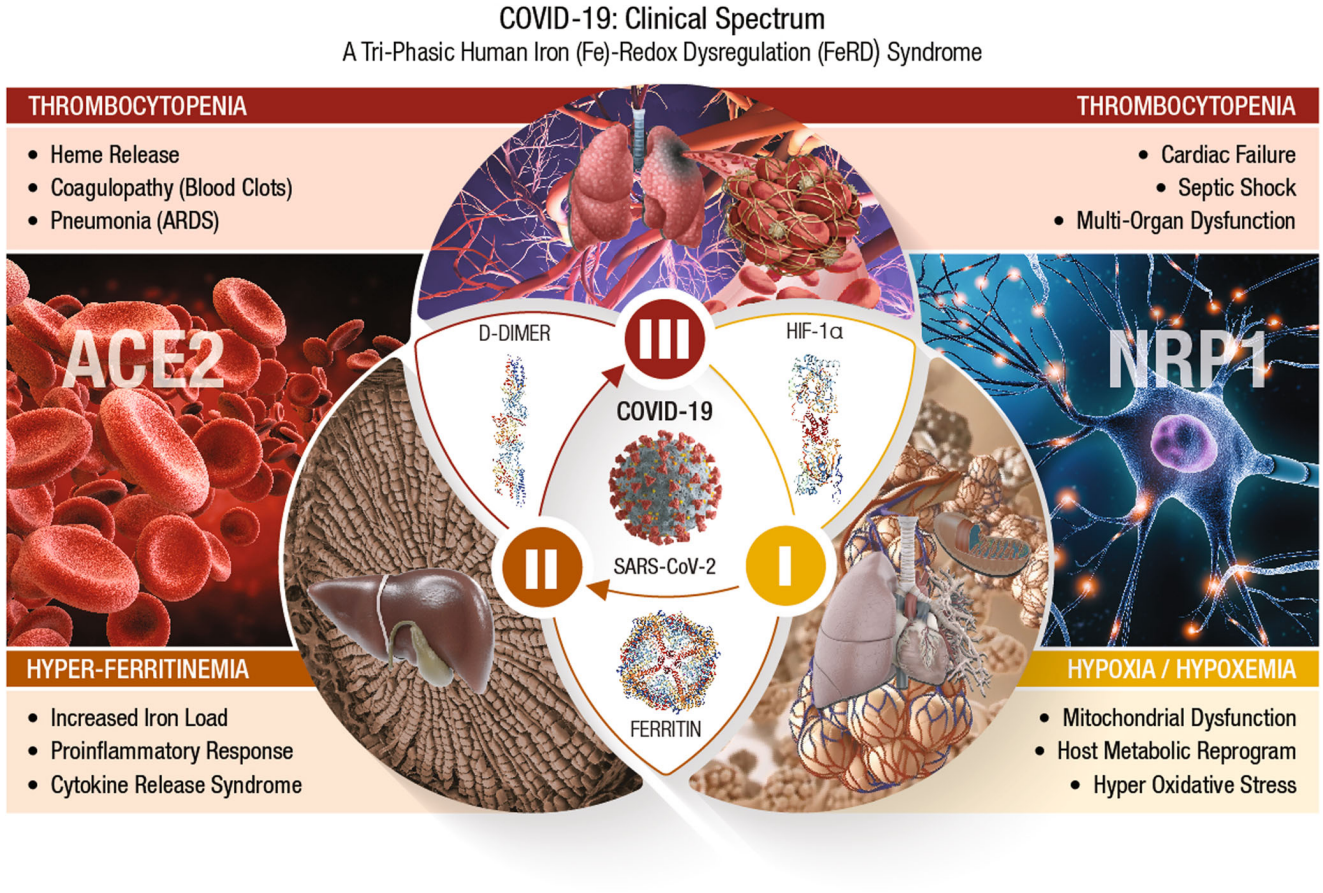
COVID-19 clinical spectrum. The symptomatic outcomes of SARS-CoV-2 infection manifest in a tri-phasic manner as iron (Fe)-redox disruptive hematological syndromes^27^. *Phase-I: Hypoxia/Hypoxemia*. Viral binding to ACE2 alters RAAS, subsequently lowers blood pressure, lung function, and reduces O_2_ transport (hypoxia) in the infected host. This condition triggers a mitochondrial metabolic shift by alteration of OXPHOS/TCA cycle and activation of anaerobic glycolysis, the ‘Warburg Effect’. This metabolic shift is regulated by HIF-1α that causes impairment of host immune response, exacerbates inflammation, and elicits tissue damage^88^. This clinical phase of COVID-19 is considered a hypoxia-induced blood disease, associated with FeRD and HMRD^27,93^. *Phase-II: Hyperferritinemia* is characterized by a hyper-inflammatory state with elevated proinflammatory cytokines, which stimulates synthesis of both ferritin and hepcidin, the ultimate mediators of FeRD^94^. The altered iron homeostasis is reflected by high iron content in reticuloendothelial cells and elevated serum ferritin levels. Such uncontrolled and dysfunctional immune response associated with macrophage activation leads to hyperferritinemia, and ‘cytokine storm’ or *cytokine release syndrome* (CRS)^97^. Hyperferritinemia, cellular redox imbalance and FeRD play a critical role in the disease progression of COVID-19,^27,98^. *Phase-III: Thrombocytopenia*. SARS-CoV-2 could invade blood vessels, induce vascular damage, and activate systemic thrombotic events with severe to fatal coagulopathies in COVID-19 patients^100^. This clinical state along with hypoxia, could cast signs of hemolysis with release of heme proteins and accumulation of free heme. Heme from hemolysis could initiate oxidative and inflammatory stress that may cause microvascular thrombosis, organ ischemia and multi-organ failure in severe COVID-19 cases^102,105^.

In accordance with its virulence spectrum and host susceptibility pattern, the symptomatic outcomes in COVID-19 patients are manifested in a tri-phasic manner as FeRD-induced hematological syndromes^27^, as shown in Fig. 2.

### COVID-19/phase-I: hypoxia/hypoxemia

SARS-CoV-2 binding to host CSRs (i.e., ACE2, NRP1) is an initial step in the pathogenesis of COVID-19. Viral binding to ACE2 receptors on alveolar epithelia affects *renin-angiotensin-aldosterone system* (RAAS), subsequently lowers the blood pressure and lung function of an infected host^85^. The reduced O_2_ transport (hypoxia) triggers a mitochondrial metabolic reprogramming/dysregulation via alteration of OXPHOS/TCA cycle and activation of anaerobic glycolysis, known as the ‘Warburg Effect’^86,87^. This shift in mitochondrial energy metabolism (or ATP synthesis) is regulated by different cellular systems, of which the *hypoxia-inducible factor* (HIF)-1α plays a critical role^88^. HIF-1α induced HMRD affects the available host energy reserves for immune function^89^. Ultimately, HIF-1α could impair host immune response, exacerbate inflammation, and inflict tissue damage. SARS-CoV-2 could evade host innate immunity and sustain intracellular viral replication cycle by altering the mitochondrial dynamics through targeting the *mitochondria-associated antiviral signaling* (MAVS) pathways^90^ HIF-1α could up-regulate *vascular endothelial growth factor* (VEGF) to cause vascular leakage, damage epithelial barriers of alveoli and vascular endothelia^91,92^. Therefore, phase-I of COVID-19 is considered a hypoxia-induced blood disorder, associated with FeRD and HMRD^27,93^.

### COVID-19/phase-II: hyperferritinemia

Severe COVID-19 is characterized by hyper-inflammation with elevated proinflammatory cytokines that stimulate the synthesis of both ferritin and hepcidin (which ultimately mediate FeRD)^94^. The iron homeostatic imbalance is reflected by high iron content in reticuloendothelial cells and elevated serum ferritin levels. When the iron-binding capacity of *transferrin* (TF) in the blood exceeds, free iron is released into plasma in a redox-active state known as the *labile plasma iron* (LPI), which forms tissue-damaging free radicals and cause fibrosis^95^. A ferritin/TF ratio >10 predicts a five-fold higher risk of ICU admission and an eight-fold higher risk for need of mechanical ventilation in COVID-19 patients^96^. A dysfunctional hyperimmune response in tandem with macrophage activation could trigger hyperferritinemia, and ‘cytokine storm’ or *cytokine release syndrome* (CRS). CRS is characterized by fulminant activation of a large number of lymphocytes that release inflammatory cytokines and result in severe tissue damage with *multi-organ dysfunction syndrome* (MODS)^97^. Hyperferritinemia, and FeRD collectively play a detrimental role in disease progression of COVID-19^27,98^. Phase II of COVID-19 is considered a wide-spectrum hyperinflammatory disease, amplified by CRS from HMRD^27^.

### COVID-19/phase-III: thrombocytopenia

Acute COVID-19 due to severe iron toxicity from oxidized iron could modulate several systemic pathways of coagulation cascade and cause thromboembolism^99^. SARS-CoV-2 could invade blood vessels, induce vascular damage, and activate systemic thrombotic events with severe to fatal coagulopathies in COVID-19 patients^100^. Such coagulopathies (or blood clots) are characterized by elevated procoagulant factors such as fibrinogen, along with high levels of D-dimers linked to increased CFR^101,102^. Hematological parameters such as *anemia of inflammation* (AI), reduced numbers of peripheral blood lymphocytes and eosinophils with increased neutrophil-to-lymphocyte ratios are recognized as major risk factors^103,104^. This clinical phase along with hypoxia, could exhibit signs of hemolysis with the release of heme proteins and accumulation of free heme. The hemolysis-derived heme could initiate inflammatory OxS that may cause microvascular thrombosis, organ ischemia and MODS in severe COVID-19^102,105^.

### COVID-19 pathobiological spectrum

The incubation period, defined as the time from infection to the onset of signs and symptoms, is a crucial index of epidemiology in understanding the pathobiological spectrum of acute SARS-CoV-2 infection, and PASC^106^. The median incubation period for COVID-19 was estimated to be 5.1 days, and 99% (101 out of every 10,000 cases) will develop symptoms after 14 days^107,108^. The median *viral clearance time* (VCT, RT-PCR negative) is 24 days. The VCT was 30 days among severe COVID-19 patients and 39 days among ICU-admitted patients^109,110^.

About 80% of SARS-CoV-2 infections are asymptomatic to mild, and many COVID-19 patients recover within 2 to 4 weeks. However, the onset of severe pneumonia and critical MODS may occur in 15 and 5% of patients, respectively, which could last for 3 to 6 weeks^111^. COVID‐19 patients may develop a wide range of clinical manifestations, including severe acute pulmonary disease, hepatic dysfunction, kidney injury, heart damage, gastro-intestinal, skeleto-muscular, pancreatic, and sensory (smell and taste) dysfunctions^112–117^. SARS-CoV-2 inflicts severe respiratory symptoms with a substantial pulmonary dysfunction, which may include severe arterial hypoxemia (low blood oxygenation) resulting in *acute respiratory distress syndrome* (ARDS)^118^. SARS-CoV-2 could also impair cardiovascular (CV) metabolism in COVID-19 patients. The viral S-protein and the ORF9b subunits could alter human cardiomyocyte metabolism and significantly impair the contractile function of the heart^119^. COVID-19 has a major impact on heart health and may lead to myocarditis or cardiac failure.

In COVID patients, the SARS-CoV-2 infection could also reach the brainstem and induce cerebral lesions as long-term sequelae^120^. Several neurological manifestations including cognitive dysfunction are often described in such patients. Thus, SARS-CoV-2 infections impact not only the respiratory organ but also inflict various bodily damage leading to shock and MODS^121^.

## Post-acute sequelae of COVID-19 (PASC) or long-COVID

*Post-acute sequelae of COVID-19* (PASC) or long-COVID refers to a wide spectrum of symptoms and signs that are persistent, exacerbated, or new clinical incidents during the time period that prolongs after acute SARS-CoV-2 infection^122,123^. About 25 to 70% of COVID-19 survivors may experience severe debilitating virus-free disease states with lingering symptoms lasting for weeks to months^2,124^. PASC affects asymptomatic, mild symptomatic, or self-quarantined (at home) individuals infected with SARS-CoV-2, as well as moderately to severely inflicted COVID-19 patients that require hospitalization and/or intensive care^4^. The incidence of PASC is estimated at 10–30% of non-hospitalized cases, 50–70% of hospitalized cases, and 10–12% of vaccinated cases^125–127^. PASC is reported in all ages, with the highest percentage of diagnoses observed between the ages 36 and 50 years. PASC is frequently diagnosed in non-hospitalized patients with mild illness, and this population represents most COVID-19 cases^5^.

After two years post-recovery, PASC continues to affect the *disability-adjusted life years* (DALYs per 1000 persons) of about 25.3% non-hospitalized and 21.3% hospitalized individuals^128^. Accordingly, the substantial cumulative burden of health loss due to persistent long-term PASC is overwhelming.

A prospective cohort study *(n* = 9764*)* conducted by *Researching COVID to Enhance Recovery* (RECOVER) consortium of the US *National Institutes of Health* (NIH) proposed a symptom-based criteria to identify and differentiate PASC cases^129^. The study identified six clinical manifestations, namely: *post-exertion malaise* (PEM) (87%), fatigue (85%), brain fog (64%), dizziness (62%), GI (59%), and palpitations (57%), as the most prominent PASC symptoms; an additional six common symptoms such as changes in sexual desire or capacity, loss of or change in smell or taste, thirst, chronic cough, chest pain, and abnormal movements were included. Other manifestations associated with selected symptoms such as dry mouth, weakness, headaches, tremor, muscle and abdominal pain, fever/sweats/chills, and sleep disturbance were also recognized.

The long-term sequelae of PASC could manifest with >200 different and overlapping clinical symptoms involving multiple organ/systems such as Pulmonary-PASC (general fatigue, dyspnea, cough, throat pain); *Cardiovascular* (CV)-PASC (chest pain, tachycardia, palpitations); *Gastrointestinal* (GI)-PASC (diarrhea, abdominal pain, nausea vomiting); Neuro-cognitive-PASC (brain fog, dizziness, loss of attention, confusion); Renal-PASC (renal failure, electrolyte disorders; Hepato-biliary-PASC; Skeleto-muscular-PASC (myalgias, arthralgias); Psychological-related PASC (post-traumatic stress disorder, anxiety, depression, insomnia); and other PASC manifestations (ageusia, anosmia, parosmia, skin rashes)^130,131^.

RECOVER study has proposed the following four multi-symptomatic PASC clusters or subgroups: *Cluster-1*—loss of or change in smell or taste*; Cluster-2*—PEM (99%) and fatigue (84%); *Cluster-3*—brain fog (100%), PEM (99%), and fatigue (94%); and *Cluster-4 with* fatigue (94%), PEM (94%), dizziness (94%), brain fog (94%), GI (88%), and palpitations (86%)^129^. Based on relapsing/remitting nature of acute- and post-COVID symptoms, an integrative classification has been proposed^132^. (i) SARS-CoV-2 infection-related acute COVID symptoms (up to 4–5 weeks), (ii) acute post-COVID symptoms (from week 5 to 12), (iii) long post-COVID symptoms (from week 12 to 24), and (iv) persistent post-COVID symptoms (lasting >24 weeks). This classification includes time reference points with predisposing intrinsic/extrinsic factors and hospitalization data in relation to post-COVID symptoms. The clinical transition from acute COVID-19 to symptomatic PASC seems to vary between hospitalized and non-hospitalized patients. In hospitalized COVID-19 patients, about 50–70% cases may continue to PASC symptoms lasting up to 3 months after hospital discharge^133^. In non-hospitalized subjects, about 50–75% may turn PASC-free one month after symptomatic onset^134^. PASC patients may also experience exercise intolerance and impaired daily function and quality of life^135^.

Based on the plethora of symptoms affecting different organs/systems, PASC-affected population could be categorized into four different clusters or sub-phenotypes: *Sub-phenotype-1* (33.8%) with cardiac and renal manifestations, *Sub-phenotype-2* (32.8%) with respiratory, sleep and anxiety disorders, *Sub-phenotype-3* (23.4%) with skeleton-muscular and nervous disorders, and *Sub-phenotype-4* (10.1%) with digestive and pulmonary dysfunctions^123,136^.

## Lon-COVID/PASC: virus-induced human metabolic reprogramming and dysregulation (HMRD)

**Fig. 3:**
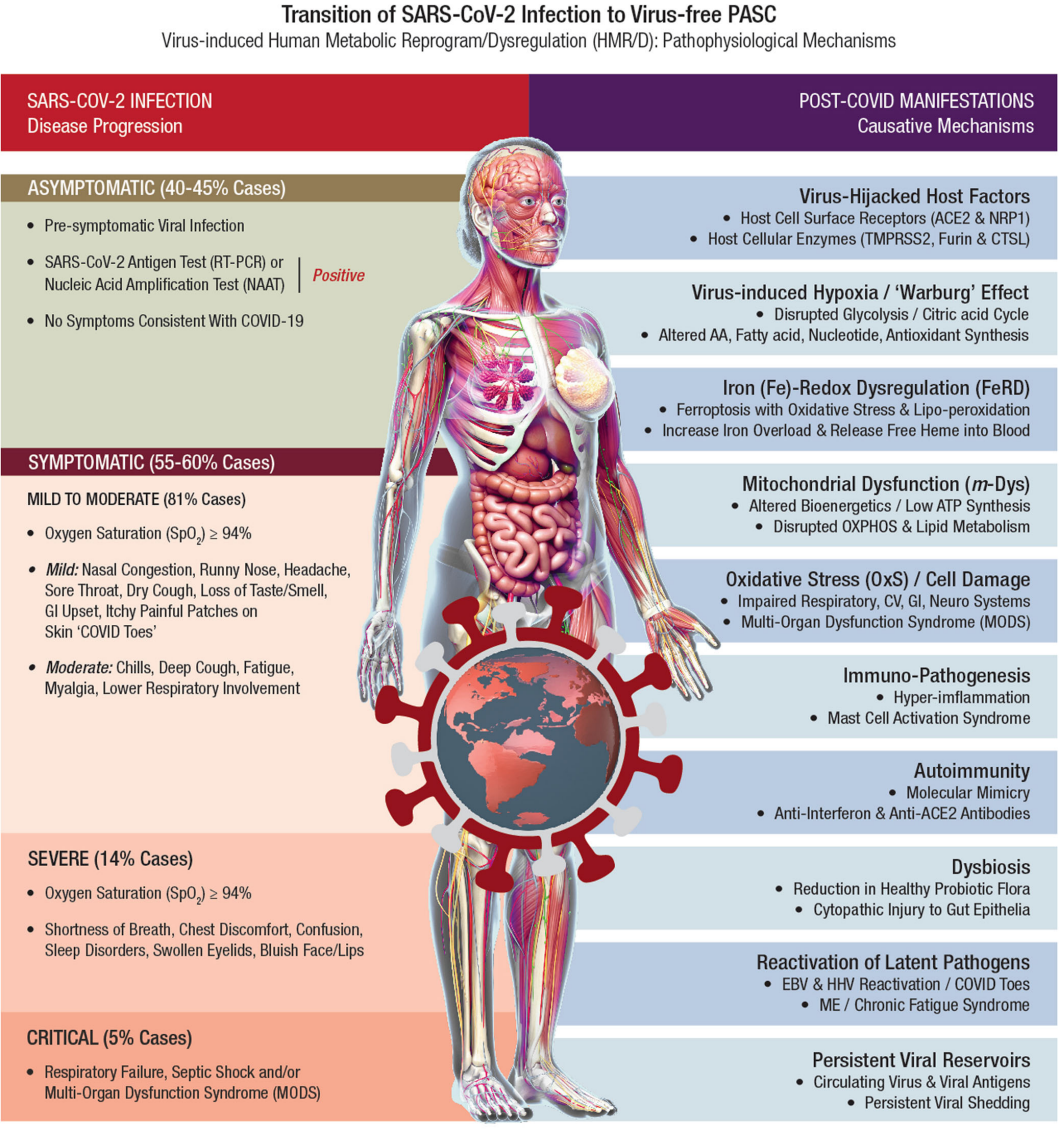
Transition of SARS-CoV-2 Infection to Virus-free PASC: Pathophysiological Mechanisms. *Post-acute sequelae of COVID-19* (PASC) or long-COVID refers to a broad spectrum of symptoms and signs that are persistent, exacerbated, or new clinical incidents in the period that prolongs after acute SARS-CoV-2 infection. In acute COVID-19, the SARS‐CoV‐2 genome and its products critically reprogram and dysregulate human metabolism (HMRD) at transcription, translation, and *post-translational modification* (PTM) levels. Interaction of SARS-CoV-2 proteins with specific host cellular targets rewires sugar-, amino acid-, lipid-, and nucleotide-metabolism(s), as well as alters or impairs bioenergetics, immune response, and redox homeostasis in the body, to facilitate viral replication and propagation^21,22^. However, several recoverees or survivors of COVID-19 (*RT-PCR negative* for SARS-CoV-2) continue to exhibit a plethora of clinical symptoms with impairment(s) of multiple organ systems. Accordingly, PASC or long-COVID is a virus-free, ‘new onset’ pathophysiological condition extending from a virus-induced HMRD. The HMRD in PASC pathology is a cumulative clinical outcome of several causative mechanisms comprising both SARS-CoV-2-derived virulence factors, as well as a multitude of host cellular factors and innate responses. A plethora of PASC clinical symptoms and related metabolic impairments indicate an involvement of different pathobiological mechanisms.

Several recoverees or survivors of COVID-19 (*RT-PCR negative* for SARS-CoV-2) continue to exhibit a plethora of clinical symptoms with impairment(s) of multiple organ systems. Accordingly, PASC or long-COVID is a virus-free, ‘new onset’ disease condition extending from an earlier virus-induced HMRD. The HMRD in PASC pathology is a cumulative clinical outcome of several causative mechanisms comprising both SARS-CoV-2-derived virulence factors, as well as a multitude of host cellular factors and innate responses. A plethora of PASC clinical symptoms and related metabolic impairments indicate involvement of different pathobiological mechanisms such as (i) virus-induced hypoxia/’Warburg’ effect, (ii) *iron (Fe)-redox dysregulation* (FeRD), (iii) *m-*Dys and altered bioenergetics, (iv) *oxidative stress* (OxS) and cellular damage, (v) immuno-pathogenesis and hyperinflammation, (vi) autoimmunity, (vii) dysbiosis, (viii) re-activation of latent pathogens, (ix) persistent viral reservoirs, and (x) viral-hijacked host cellular factors^22,27,137,138^. A wide range of pathophysiological mechanisms involved in the transition of SARS-CoV-2 Infection to virus-free PASC clinical condition is shown in Fig. 3.

### Virus-induced hypoxia/’Warburg’ effect

**Fig. 4:**
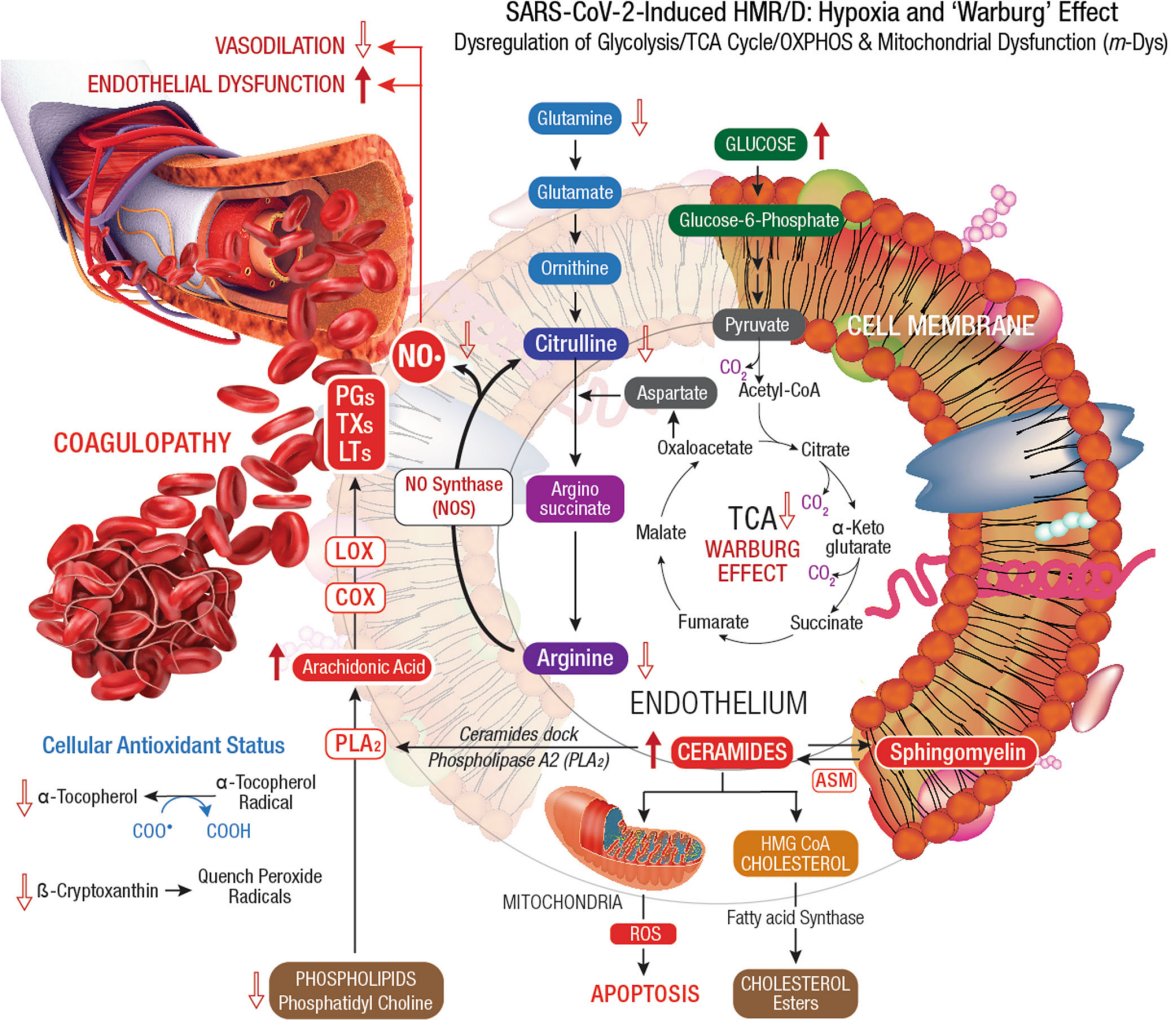
Virus-induced HMRD: hypoxia and ‘Warburg’ effect. Dysregulation of glycolysis/TCA cycle is a key feature of HMRD. COVID-19 patients exhibit elevated serum glucose levels with an upregulation of glycolytic intermediates. Glutamine deficiency and hyaluronan over synthesis are HMRD-induced metabolic events in SARS-CoV-2 infection^785^. M1 macrophages express *nitric oxide synthase* (NOS), which oxidizes arginine to *nitric oxide* (NO•) and citrulline. NO• modulates vascular tone, blood pressure and hemodynamics. Disrupted arginine metabolism further down-regulates NO• synthesis, aggravates endothelial dysfunction and triggers severe coagulopathies in COVID-19^184^. Downstream generation of amino acids ornithine, citrulline, arginine in the circulation also indicates a severe renal dysfunction^51^. Degradation of sphingomyelin by *acid sphingomyelinase* (ASM) generates stimulatory ceramides, the docking molecules for *phospholipase A2* (PLA_2_). The hydrolysis of phospholipids (i.e., *phosphatidyl choline*) by PLA_2_ elevates *arachidonic acid* levels, a precursor for broad spectrum eicosanoids produced by *cyclooxygenase* (COX) and *lipoxygenase* (LOX) enzymes. These enzymes further convert arachidonic acid to *prostaglandins* (PGs), *thromboxanes* (TXs), and *leukotrienes* (LTs), which collectively contribute to the development of vascular inflammation and disease severity in COVID-19^786^. Virus-induced HMRD alters host lipid metabolism with major impact on sphingolipid and arachidonic acid pathways^787^. A decline in fat-soluble antioxidants’ vitamin E and carotenoids could compromises ROS quenching capacity in the plasma membrane, causes lipid peroxidation and OxS. Elevated serum lipase levels indicate damaging clinical outcomes in COVID-19 patients^788^. The virus-induced HMRD alternations to glucose, amino acid, and lipid metabolism could aggravate the severity of COVID-19 and may extend to PASC pathology.

SARS-CoV-2 hijacks host cellular metabolic machinery to extract adequate energy and carbon skeletons to facilitate viral entry and facilitate molecular constructions for viral progeny inside a host cell for replication and propagation. The SARS-CoV-2 infection initiates complex human host-pathogen interactions and alters mitochondrial function with significant disruption of glycolysis/TCA cycle (Warburg effect), affecting several metabolic pathways of *amino acid* (AA), *fatty acid* (FA), nucleotide, and antioxidant synthesis^139,140^. The virus-induced hypoxia/Warburg effect could potentially compromise endocrinal, cardiovascular, neurocognitive, gastrointestinal, pulmonary, and reproductive functions that demand high levels of mitochondrial O_2_ consumption, OXPHOS, and ATP reserve. Failure to reset hypoxia/Warburg effect after viral clearance in COVID-19 survivors, could eventually evoke PASC with metabolic impairments including new onset T2DM, myocardial infarction, *chronic fatigue syndrome* (CFS), brain fog, and blood clotting issues^141^. Accordingly, PASC could be described as a SARS-CoV-2-induced chronic and self-perpetuating comprised state of *m-*Dys, where OxS potentially drives inflammation and shifts energy metabolism towards glycolysis while down-regulating OXPHOS^27,142,143^. Long-term consequences of virus-induced hypoxia/Warburg effect could amplify potential risks of HMRD with chronic multi-organ impairments in PASC (Fig. 4).

### Iron (Fe)-redox dysregulation (FeRD)

During SARS-CoV-2 infection, free iron released into the circulation induces inflammation of alveolar macrophages and causes oxidative damage to the lungs^144^. Increased iron load increases blood viscosity with recurrent diffused micro/macro circulatory thrombosis leading to high levels of D-dimers in COVID-19 patients. Altered iron metabolism, iron-restricted erythropoiesis from hyperinflammation causes FeRD^27,145^. In COVID-19 patients, FeRD could trigger several clinical manifestations including (i) decrease functional *hemoglobin* (Hb), (ii) increase cellular iron overload, (iii) release free toxic heme into the circulation, (iv) manifest hypoxemia and systemic hypoxia, (v) reduce *nitric oxide* (NO•) synthesis, (vi) activate coagulation pathway(s), (vii) trigger ferroptosis with OxS and lipid peroxidation, and (viii) induce mitochondrial degeneration^27,146^.

On the other hand, viral protein sequences could form complexes with porphyrin, affect heme on the 1-β chain of Hb, and release free iron^147^. SARS-CoV-2 *envelope* (E) protein directly binds to heme (from Hb) released from damaged erythrocytes and lysed phagocytes^148^. The viral genomic ORF8 protein could interact with the 1β-chain of Hb, capture the porphyrin and inhibit heme metabolism in the body^149^. Such an array of SARS-CoV-2 interactions with Hb could induce hemolysis and/or form complexes with released heme, generate dysfunctional Hb (hemoglobinopathy) with reduced ability to transport O_2_/CO_2_ and lead to O_2_ deprived multi-faceted syndromes, including coagulation disorders^146,150^. In severe stages of COVID-19, other Hb-associated markers such as bilirubin and ferritin progressively increase and worsen the clinical outcomes.

The FeRD-induced hyperferritinemia strongly correlates with different inflammatory phases of SARS-CoV-2 infection^98,101,151^. In SARS-CoV-2 infected patients, the plasma levels of ferritin and IL-6 steadily decrease with gradual recovery from COVID-19^152,153^. FeRD is highly prevalent among hospitalized COVID-19 patients and this clinical condition may continue for weeks or even months in PASC patients. Biomarkers of iron metabolism (i.e., ferritin, *transferrin* (TF), *lactoferrin* (LF), etc.) and Hb could provide risk stratification strategies for COVID-19 management. FeRD determinations are specific and sensitive to predict disease severity in COVID-19 and PASC patients^27,154^.

### Mitochondrial dysfunction (*m*-Dys)/altered bioenergetics

The mitochondrion is the cellular powerhouse involved in *oxidative phosphorylation* (OXPHOS), ATP synthesis, and regulation of *calcium* (Ca^2+^) signaling, redox homeostasis, lipid metabolism, cell differentiation, immune system, apoptosis, and cellular senescence (aging)^155,156^. These vital processes are perturbed when the host cellular machinery is hijacked by SARS-CoV-2, which ultimately manifests as *mitochondrial dysfunction* (*m*-Dys). SARS-CoV-2 infection leads to *m*-Dys including mitochondrial membrane depolarization, mitochondrial permeability transition pore opening, increased release of *reactive oxygen species* (ROS), and disrupted. mitochondrial redox homeostasis^157,158^. SARS-CoV-2 infection also affects fusion/fission kinetics, size, structure, and distribution of mitochondria in the infected host cells. COVID-19 patients with underlying primary mitochondrial disease and secondary *m*-Dys are prone to increased disease severity and CFR compared to patients with healthy mitochondrial functions^159^. Thus, *m*-Dys could heavily compromise host bioenergetics with detrimental consequences on COVID-19 and long-term PASC patients^160,161^.

After host cell entry, the ORF9b of SARS-CoV-2 RNA could directly manipulate mitochondrial function to evade host cell immunity, facilitate viral replication and trigger the onset of COVID-19. The ORF9b could further manipulate host mitochondria by releasing mitochondrial DNA (mt-DNA) into the cytoplasm to activate mt-DNA-induced inflammasome and suppress innate as well as adaptive immunity^162^. SARS-CoV-2 may also manipulate mitochondrial function via ACE2 regulation. A decline in ACE2 function in aged individuals, coupled with the age-associated deterioration in mitochondrial functions results in chronic metabolic disorders like diabetes or cancer, and predisposes the host for increased susceptibility to infection, vulnerability to health complications, and intensifies the risk of mortality^163^.

SARS-CoV-2 invades mitochondria and evades host defense by the formation of double-membrane vesicles. These virus-induced vesicles could damage mitochondrial membrane integrity, release mt-DNA into circulation, compromise innate immunity, and trigger an exacerbated pro-inflammatory response in COVID-19 patients^164^. SARS-CoV-2 infection could alter mitochondrial function(s), activate TLR9 signaling, induce hyper-inflammation and disrupt endothelial activity^165^. The viral infection could also cause rapid T lymphocytopenia with functional impairment of T cells, which may onset OxS, pro-inflammatory state, cytokine production, and apoptosis^166,167^. Hyper-inflammation (with CRS or cytokine storm) due to massive outburst of ROS, is a prominent clinical feature of COVID-19^145^. The mitochondrion is a significant source of ROS in human cellular metabolism that could trigger the onset and development of cytokine storm^168^.

SAR-COV2 could induce *m*-Dys, activate mitochondrial-dependent intrinsic apoptotic pathways, and cause microglial and neuronal apoptosis leading to neuropathological symptoms in COVID-19 and PASC patients^169,170^. In the current pandemic, about 40% of COVID-19 patients demonstrated neurological symptoms, lingering neuro-inflammation, where neuronal damage in PASC patients has emerged as a novel syndrome, the ‘Neuro-COVID’^169,171^. Peripheral blood monocytes of such patients demonstrate altered bioenergetics and reduced basal respiration, reduced spare respiratory capacity, and decreased proton leak^172^. The *m*-Dys-induced exercise intolerance with elevated arterial blood lactate levels and reduced fatty acid β-oxidation rates is a major health issue in PASC^173^. These patients complain about chronic fatigue during exercise, despite no obvious heart or lung abnormalities^174^.

During the aging process, progressive *m*-Dys occurs due to the loss of *thioretinaco-ozonide-oxygen-ATP* complex from mitochondrial membranes through the opening of mitochondrial permeability transition pore^175^. Disruption in mitochondrial OXPHOS could elevate OxS and activate sepsis cascade through HIF-α/Sirtuin pathway. Due to *m*-Dys, senescent cells fail to meet the hyper-metabolic demands of sepsis in COVID-19 patients. A decline in mitochondrial function in the aging population could be a possible risk factor for increased mortality in COVID-19 and PASC^176^. Furthermore, as a hallmark of the aging population, *m*-Dys could onset chronic inflammation with massive cytokine release and cause multi-organ failure with fatal outcomes in elderly COVID-19 patients^177^. Age-related comorbidities (metabolic syndromes) such as, obesity, T2DM, asthma, and CVD, could also increase severity and mortality in elderly COVID-19 patients. Preventive therapies to improve mitochondrial turnover, dynamics and activity could prove beneficial in protection against COVID-19 severity^178^. Therefore, nutritional targeting of mitochondrial metabolism could showcase as an effective treatment regimen for PASC management.

### Oxidative stress (OxS)/cellular damage

*Oxidative stress* (OxS) is a nonspecific pathophysiological condition that reflects a redox imbalance between increased production of ROS (free radicals) and the inability of antioxidant defenses to neutralize the reactive intermediates or to repair the ensuing damage^179,180^. ROS disrupts cellular metabolism by inflicting DNA strand breaks, protein degradation, lipid peroxidation, and cellular damage^181^. Combined with inflammation, OxS contributes to cardinal patho-mechanisms of both COVID-19 and PASC^182^.

After SARS-CoV-2 infection, the viremia stage could increase OxS, elevated levels of ROS/inflammation markers (i.e., peroxide, NO•, carbonylated proteins, and IL-6) and inflict severe cellular/tissue damage. This clinical condition may compromise mitochondrial functions and trigger apoptosis of leukocytes^183^. Hyper-inflammation, pro-oxidant cytotoxic milieu, and early apoptosis of leukocytes from SARS-CoV-2 infection, could cause severe endothelial-alveolar injury and MODS^184^. PASC patients exhibit a wide range of tissue/organ damage involving pulmonary, cardiovascular, neuro-cognitive, GI, reproductive, and dermatological systems^5,127,185^.

#### Pulmonary damage in PASC

SARS-CoV-2 infection of alveolar epithelia could induce cytokine storm and OxS (with ROS release) resulting in severe lung damage^186^. Viral envelope proteins could also trigger abnormal immune response, dysregulate type-1 IFN synthesis, increase NETosis and cause organ injury via microthrombi formation^187^. In COVID-19 survivors, respiratory abnormalities with reduced total lung capacity and airway dysfunction (i.e., dyspnea, chronic cough, and reduced exercise capacity) may persist as chronic manifestations^188^. About 36% of PASC patients complain of shortness of breath and about 26% develop lung impairment. In the long term, virus-induced hyper-inflammation and subsequent disruption of coagulant pathways could increase the risk of thrombosis in PASC patients^138^.

#### Cardiovascular (CV) damage in PASC

CV complications are prevalent among PASC patients since ACE2 receptor-rich cardiomyocytes provide SARS-CoV-2 direct access to the heart. Disease severity during acute COVID-19 establishes the clinical basis for the onset of CV-PASC. Persistent myocardial inflammation with elevated cardiac troponin levels (2 months after disease onset) is a distinct feature among COVID-19 patients^6^. In acute COVID-19, prominent CV conditions such as myocardial injury, myocarditis, acute heart failure, cardiomyopathy, cardiac dysrhythmias, and venous thromboembolic events may occur^189^. Three months after hospital discharge, about 30% of COVID-19 patients demonstrate adverse ventricular remodeling, which indicates cardiac sequelae^190,191^. Causative mechanisms for CV-PASC include chronic inflammation due to viral persistence in heart tissue, molecular mimicry invoking autoimmune responses against cardiac antigens, and ongoing endothelial/microvascular dysfunction^192^. Many PASC patients (89%) report CV symptoms including chest pain (53%), palpitations (68%), and new onset of *postural orthostatic tachycardia syndrome* (POTS, 31%)^193^.

#### Neuro-cognitive damage in PASC

SARS-CoV-2 crosses the *blood-brain barrier* (BBB), invades the brain stem, damages brain parenchyma, and manifests neuro-COVID sequelae^171^. Multiple mechanisms are proposed in the onset and progression of neuro-COVID including hypoxia, hyper-coagulability, endothelial dysfunction, nerve injury, neuro-inflammation, and neurotropism, where all conditions are induced by SARS-CoV-2 infection. Impaired neuron-glial homeostasis, neuron axonal damage, astrogliosis, and microgliosis, are frequent manifestations in neuro-COVID^194,195^.

During host cell entry, the viral S-protein disrupts BBB function, damages neurons, and activates brain mast cells^196^. Neuro-invasion of SARS-CoV-2 occurs via *transcribrial* (nose) route with damage to olfactory mucosa, and olfactory nerves, ultimately manifesting into *anosmia* (loss of smell)^197,198^. COVID-19 patients also display diffused white matter damage, microglial activation, and neuroinflammation at different CNS regions with olfactory neuritis (25%), nodular brainstem encephalitis (31%), and cranial nerve neuritis (6%)^199^. Reactive gliosis, astrocytosis, and microglial activation, along with neuroinflammation gradually advances from COVID-19 to PASC^200^. Fatigue, cognitive dysfunction (brain fog, memory issues, attention disorder) and sleep disturbances are prominent clinical features of PASC. Psychiatric manifestations (sleep disturbances, anxiety, and depression) are also common and significantly increase in due course of neuro-PASC development^201^.

#### Multi-organ dysfunction syndrome (MODS)

MODS due to virus-induced extensive tissue injury has long-term implications in COVID-19 survivors and in PASC. Patients recovered from COVID-19 show increased risk and about 1-year burden of GI disorders such as irregular bowel movement, acid-related illnesses (i.e., dyspepsia, gastroesophageal reflux condition, peptic ulcers), acute pancreatitis, hepatic and biliary dysfunction^202^. Prolonged GI manifestations in COVID-19 and PASC are attributed to dysbiosis (microbiome imbalance), immune dysregulation and delayed viral clearance from the gut. Bi-directional interactions between respiratory mucosa and gut microbiota (‘Gut-Lung Axis’) plays a major role in the progression of GI-PASC^203,204^. *Acute kidney injury* (AKI) is highly prevalent among discharged COVID-19 patients, and 35% of the recovered patients show reduced kidney function and may require kidney replacement therapy^193,205^. Virus-induced hyper-inflammation with complement activation in kidney tissue could inflict focal segmental glomerulo-sclerosis with glomerular involution and lead to AKI^138^.

#### Myalgic encephalomyelitis/chronic fatigue syndrome (ME/CFS)

ME/CFS is defined as persistent or relapsing fatigue for at least six months, which is not resolved by rest, and causes a substantial reduction in the ‘Activity of Daily Living’ (ADL)^206^. It is a hypometabolic state with impairment in multiple metabolic pathways linked to *m*-Dys with impaired OXPHOS and reduced ATP production^207^. Cognitive dysfunction, depression, and prolonged fatigue are the hallmark of ME/CFS^208^. Aberrant *mast cell activation* (MCA) could mediate hyper-inflammation in COVID-19 and initiate severe cascades of immune responses that trigger allergic flare-ups in PASC^209^.

### Immuno-pathogenesis/hyper-inflammation

SARS-CoV-2 infection could disrupt host immune homeostasis, inflict tissue injury, and may persist during the post-recovery phase of COVID-19 survivors and manifest as PASC^210^. Cell-mediated immune responses with antigen-specific T cells decrease in COVID-19 patients and affect viral clearance from infected host cells^211^. Cytotoxic T cells elevate in peripheral blood and bronchoalveolar lavage of PASC patients with severe airway dysfunction with persistent respiratory symptoms that last for 3 to 6 months^212^. SARS-CoV-2 induced T-cell imbalance resolve over time; however, the markers upregulated from T-cell exhaustion may remain up to 1 year in PASC patients^213,214^.

Elevated levels of neutrophils with *neutrophil extracellular traps* (NETs and NETosis) and associated immune-thrombosis are prominent features of COVID-19 pathology^215,216^. Monocytes and macrophages mediate lung fibrotic tissue injury, a drastic consequence in the immunopathogenesis of SARS-CoV-2 infection^217^. Myeloid cells may also incite local fibrosis-mediated tissue injury and sustain proinflammatory cytokine levels contributing to the clinical development of PASC. COVID-19 patients with severe disease exhibit increased monocyte counts with higher frequencies of classical monocytes, lower frequencies of intermediate/non-classical monocytes and elevated plasma levels of *C-reactive protein* (CRP) and serum TF in comparison to mild disease. This abnormal immune response may persist for >6 months after COVID-19 recovery^218^. Monocyte alterations in acute COVID-19 patients include aberrant expression of *leukocyte migration molecules* that extend to convalescence and correspond to specific symptoms of PASC. Monocytes from PASC patients with ongoing fatigue show a sustained reduction of prostaglandin-generating enzyme, the *cyclooxygenase 2* (COX-2)^219^. Circulating monocytes may remain dysregulated, especially in convalescent subjects for 1 to 3 months of post-COVID.

‘Cytokine storm or CRS’, a clinical state of hyper-inflammation, is a prominent feature of COVID-19 severity, linked to respiratory dysfunction, ARDS with adverse disease outcomes^220,221^. About 10% of patients recovered from COVID-19 show persistent symptoms up to 6 months after initial SARS-CoV-2 infection. Cytokine storm, in tandem with lymphopenia, lymphocyte dysfunction, and granulocyte/ monocyte abnormalities, could increase the disease severity of COVID-19^222^. Immune cytokine signatures of PASC patients reflect an ongoing chronic inflammation and angiogenesis with elevated plasma levels of IL-17a, stem cell factor, IL-12p70, IL-1β, *macrophage inflammatory protein-1β* (MIP-1β), *brain-derived neurotrophic factor* (BDNF), and VEGF^223^ Also, among other immune mediators, reduced levels of cortisol strongly correlate with pulmonary-PASC symptoms^224^. Hyperimmune activation and autoimmunity are considered as potential causative factors in the onset of PASC^225^.

### Autoimmunity

In COVID-19 patients, autoantibodies against nuclear bodies (*auto-nuclear antibodies*—ANA), phospholipids, *type I interferon* (IFN), *melanoma differentiation-associated protein 5* (MDA5), and ACE2 have been reported^226^. These autoantibodies against immunomodulatory proteins (including cytokines, chemokines, complement components and cell-surface proteins) could attack tissues of patients, thereby impair host cell signals, perturb immune function, damage organ systems, and increase COVID-19 severity^36^. *Human leukocyte antigen* (HLA) genetic polymorphism has also been observed in COVID-19 patients^227^. HLA polymorphism plays a key role in the onset of several autoimmune diseases^228^. Evidently, SARS-CoV-2 infection could elicit auto-inflammatory and autoimmune disorders such as *Guillain-Barré syndrome* (GBS), autoimmune hemolytic anemia, immune thrombocytopenic purpura, and *Kawasaki disease* (KD)^12,229,230^. Mechanisms of COVID-19-derived autoimmune disorders include (i) viral-mediated host hyper-immune response, (ii) virus-induced excessive NETs formation with neutrophil-associated cytokine responses, and (iii) the molecular mimicry between viral antigenic components and host molecules^231^.

Latent autoimmunity correlates with humoral responses against SARS-CoV-2 infection and autoimmunity has emerged as a prominent feature of PASC pathology^232^. Long-term persistence of immune activation and proinflammation with latent and overt autoimmunity are etiological factors in clinical manifestation(s) of PASC^233^. About 20 distinct autoantibodies that target *G-protein-coupled receptors* (GPCR) of the CNS and ACE2/RAAS-related molecules were linked to the clinical severity of PASC^234^.

### Dysbiosis

Gut dysbiosis is defined as the reduction in diversity of GI microflora or depletion of autochthonous or host commensal beneficial bacteria with an enrichment of microbial pathogens that may alter host susceptibility to SARS-CoV-2 infection^235,236^. Impairment of *short-chain fatty acid* (SCFA) and l-isoleucine biosynthesis in gut microbiome persists beyond 30 days after recovery from COVID-19, which could contribute to persistent leaky gut and dysbiosis in PASC patients^237,238^. Notably, even after viral clearance, more than half of patients suffer from PASC with persistent dysbiosis, deregulated GI metabolism and compromised host immune response^14,127^. Gut dysbiosis and disrupted intestinal barrier function could worsen pulmonary symptoms, augment neurological or hepatic inflammation through translocation of endotoxins and bacteria via portal veins^239–241^. Dysbiosis of gut microbiome with ensuing gut barrier dysfunction could severely impact patho-physiologies of both COVID-19 and PASC^242^.

The GI tract is the largest immunological organ in the body and any aberrant immune response to SARS-CoV-2 infection induced by resident microflora could affect recovery from COVID-19. Dysbiosis may lead to GI impairment with persistent symptoms of diarrhea and abdominal pain^243^. Gut dysbiosis could also increase susceptibility to respiratory infections, alter immune responses and affect lung homeostasis (the ‘Gut-Lung Axis’). Persistent gut dysbiosis after resolution of COVID-19 may be linked to PASC, particularly to neurological manifestations^244^. In a prospective follow-up study from Wuhan, China, patients (*n* = 187) recovered from COVID-19 demonstrated a strong correlation to gut microbiota dysbiosis and PASC symptoms, 1-year after hospital discharge^245^. In a subset of patients recovered from COVID-19, long-term dysbiosis correlated with PASC symptoms, especially fatigue, joint pain, diarrhea, headache, depression, and anxiety^246^. Dysbiosis in tandem with excess antibiotic use during the pandemic possibly have contributed to an array of PASC manifestations^247^. Since the diversity of gut microbiota erodes during aging, dysbiosis could also be a reason for high susceptibility of older adults to severe COVID-19^248^.

SARS‐CoV‐2 infection inflicts sustained metabolic damage to gut microbiome and GI function; therefore, opportunistic pathogens could selectively enrich in fecal microflora of COVID-19 patients^249,250^. Fecal enrichment of bacterial pathogens, such as *Coprobacillus spp*., *Clostridium ramosum*, and *Clostridium hathewayi*, directly correlates with the severity of SARS‐CoV‐2 infection. Conversely, symbiotic gut microflora (*Bifidobacteria, Roseburia* and *Faecalibacteria*) with prominent immunomodulatory functions, are extinguished from the gut of PASC patients^251^. Accordingly, SARS-CoV-2 infection could inflict direct cytopathic injury to gut epithelia and elicit indirect immune-mediated damage to endothelial cells^252^. SARS-CoV-2 could also induce GI inflammation, dysregulate intestinal ACE2 activity, and/or infect gut microflora (similar to bacteriophage-type transduction), as three potential inter-connected mechanisms in gut dysbiosis in PASC^253^. Effective clinical management of PASC is contingent upon the following critical evaluation of dysbiosis: (i) the duration of gut dysbiosis after COVID recovery, (ii) the link between gut dysbiosis and long-term persistent symptoms, and (iii) the possible adverse health effects from enriched or depleted specific gut microflora on COVID-19 recovered individuals^254^. Therefore, reversal of dysbiosis and restoration of normal GI function could alleviate PASC symptoms and support patient recovery.

### Reactivation of latent viral pathogens

Since the onset of COVID-19 pandemic, a strong correlation between SARS-CoV-2 infection or COVID-19 vaccination and herpesvirus co-infection/reactivation has been reported^255^. To date the reactivation of eight *human herpesviruses* (HHVs) have been identified, including *Herpes Simplex Virus* types 1 (HSV‐1) and 2 (HSV‐2), *Varicella‐Zoster Virus* (VZV or HHV‐3), *Epstein-Barr Virus* (EBV or HHV‐4), *Cytomegalovirus* (HCMV or HHV‐5), HHV‐6, HHV‐7, and *Kaposi’s Sarcoma‐associated Herpesvirus* (KSHV or HHV‐8). Almost 100% of the adult population in the world is infected with at least one HHV during their life^256^. A meta-analysis (*n* = 32 studies) has estimated the prevalence of HHV reactivation in hospital-ICU-admitted COVID-19 cases at 38% for HSV, 19% for CMV, 45% for EBV, 18% for HHV-6, 44% for HHV-7, and 19% for HHV-8, respectively^257^.

The incidence of HHV reactivation was found high among patients admitted to the ICU for severe COVID-19 and among individuals administered with COVID-19 vaccine^258^. Simultaneous occurrence of cytokine storm and immune suppression during SARS-CoV-2 infection may lead to reactivation of latent HHV in the body. Lymphopenia with reduced CD8+ levels and elevated CD4 + /CD8+ ratio indicate the severity of COVID-19^215,259^. This clinical condition leads to an immune-suppressed state, which could ultimately trigger the reactivation of latent HHV and aggravate SARS-CoV-2 infection^260^. In addition to viral co-infection, anti‐COVID‐19 therapies (i.e., azithromycin, nafamostat mesylate, and remdesivir) could activate various cell signaling pathways and trigger viral lytic reactivation^261^. Remdesivir, a widely administered anti‐COVID‐19 drug, is shown to induce lytic reactivation of KSHV and EBV, from virus‐associated lymphoma cells^262^.

SARS-CoV-2 infected patients demonstrate a wide spectrum of cutaneous manifestations, including maculopapular or perifollicular rash, urticaria, vesicles, petechiae, purpura, livedo racemosa, and pseudo-chilblains, often referred to as the ‘COVID toes’^263,264^. These cutaneous manifestations of COVID-19 are reportedly associated with reactivation of latent HHVs^265,266^. Reactivation of HSV-1 coincides with decreased expression of IFN-stimulated genes and concurrent increase in highly activated T-lymphocytes during acute stages of SARS-CoV-2 infection^267^. Reactivation of EBV could enhance the severity of SARS-CoV-2 infection. SARS-CoV-2 infected patients with EBV co-infection are prone to high fever with elevated levels of CRP, and *aspartate aminotransferase*^268^. A study from China reported higher mortality rates in COVID‐19 cases with EBV reactivation (29.4%) compared to EBV-negative patients (8.1%)^269^.

An exhausted dysfunctional antiviral immune response from SARS-CoV-2 infection could trigger reactivation of human adenovirus with a sequelae effect of ME/CFS in PASC patients^270^. Immuno-compromised individuals susceptible to HHV infection are at higher risk for SARS-CoV-2 infection, PASC, are vulnerable to develop virus‐associated cancers. Several HHVs, such as KSHV, and EBV are oncogenic viruses; therefore, a follow‐up surveillance of COVID‐19 survivors, PASC patients, and vaccinated individuals for possible risk(s) of latent viral reactivation is an important preventive public health strategy^258^.

### Persistent viral reservoirs

Infectious viral particle clears out and remain undetectable in the body for most COVID-19 cases; however, among certain patients, SARS-CoV-2 could persist for months after post-recovery^271^. Accordingly, total clearance of SARS-CoV-2 RNA or its protein antigens from host infected tissue may take a longer time, while the virus and its antigenic fragments continue to remain dormant for extended periods of time in the body. The persistence of SARS-CoV-2 or its viral components in the body could trigger a dysregulated immune response and proinflammatory cytokine release, which may cause chronic low-grade inflammation and MODS. These acute sequelae also have a genetic basis that may predispose COVID-19 survivors to a compromised immune status consequently affecting viral clearance^272^.

Multi-organ viral tropism predominantly in cells expressing ACE2, TMPRSS2, or both has been reported^82,83^. Viral shedding (as detected by RT-PCR) may be prolonged in certain tissues of post-COVID patients for an extended duration in the lower respiratory tract (59 days), serum (60 days), upper respiratory tract (83 days), and feces (126 days)^273^. Such viral persistence could serve as a chronic trigger for inflammation and cellular activation that may further inflict tissue damage and elicit PASC-related symptoms^274^. In the long-term persistence of COVID-19-associated anosmia (loss of smell), viral transcripts are detected in the inflamed olfactory mucosa. Viral persistence and associated inflammation in olfactory neuro-epithelium may account for prolonged or relapsing symptoms in PASC, such as anosmia^275^. Delayed immune clearance of SARS-CoV-2 antigen(s) or duration of viral antigen burden in the upper respiratory tract and other anatomical sites during acute COVID-19 could be linked to the development of PASC^276^.

Long-term shedding of SARS-CoV-2 is widely reported, even after resolution of symptomatic COVID-19. The continuous replication of live SARS-CoV-2, its viral RNA, or viral protein fragments could play a major role in the clinical onset of PASC. SARS-CoV-2 RNA could persist for several weeks in the respiratory tract of COVID-19 survivors^274^. Viral replication has been reported in multiple respiratory and non-respiratory tissues, including the brain. Persistent shedding of SARS-CoV-2 was detected for months in the feces of patients recovered from COVID-19, regardless of GI symptoms^277^. In gut mucosa of mild to acute cases of COVID-19 (with IBD as comorbidity), the persistence of SARS-CoV-2 RNA was detected in ~70% PASC patients, whereas the viral *nucleocapsid* (N) protein was found in ~50% of PASC patients after seven months of post-recovery^278^. Such SARS-CoV-2 antigen persistence in infected tissues could possibly trigger immune perturbations that may contribute to the development of PASC.

Also, persistent viral RNA has been detected in multiple tissues of recovered patients even months after the onset of COVID-19. In a cohort of COVID-19 patients with persistent symptoms, about 45% showed detectable plasma SARS-CoV-2 RNA. Viral RNA was also found in blood, stool, and urine of PASC patients^279^. Spike and/or viral RNA fragments could persist in COVID-19 recoverees up to 12 months or longer^280,281^. The S1 antigen in peripheral blood monocytes could remain up to 15 months after SARS-CoV-2 infection^282^. The S-protein of SARS-CoV-2 contains structural motifs that affect T-cell receptors and trigger hyperinflammatory responses observed in severe COVID-19 and *multi-system inflammatory syndrome in children* (MIS-C)^283^. Spike may not activate cytokine storm in PASC patients; however, it could impair endothelial function via down-regulation of ACE2 and disrupt the integrity of BBB^284,285^. In summary, viral persistence could play a major role in PASC, considering the ability of the SARS-CoV-2 pathogen to infect and reinfect individuals over a lifetime^274^.

### Virus-hijacked host cellular factors

**Fig. 5:**
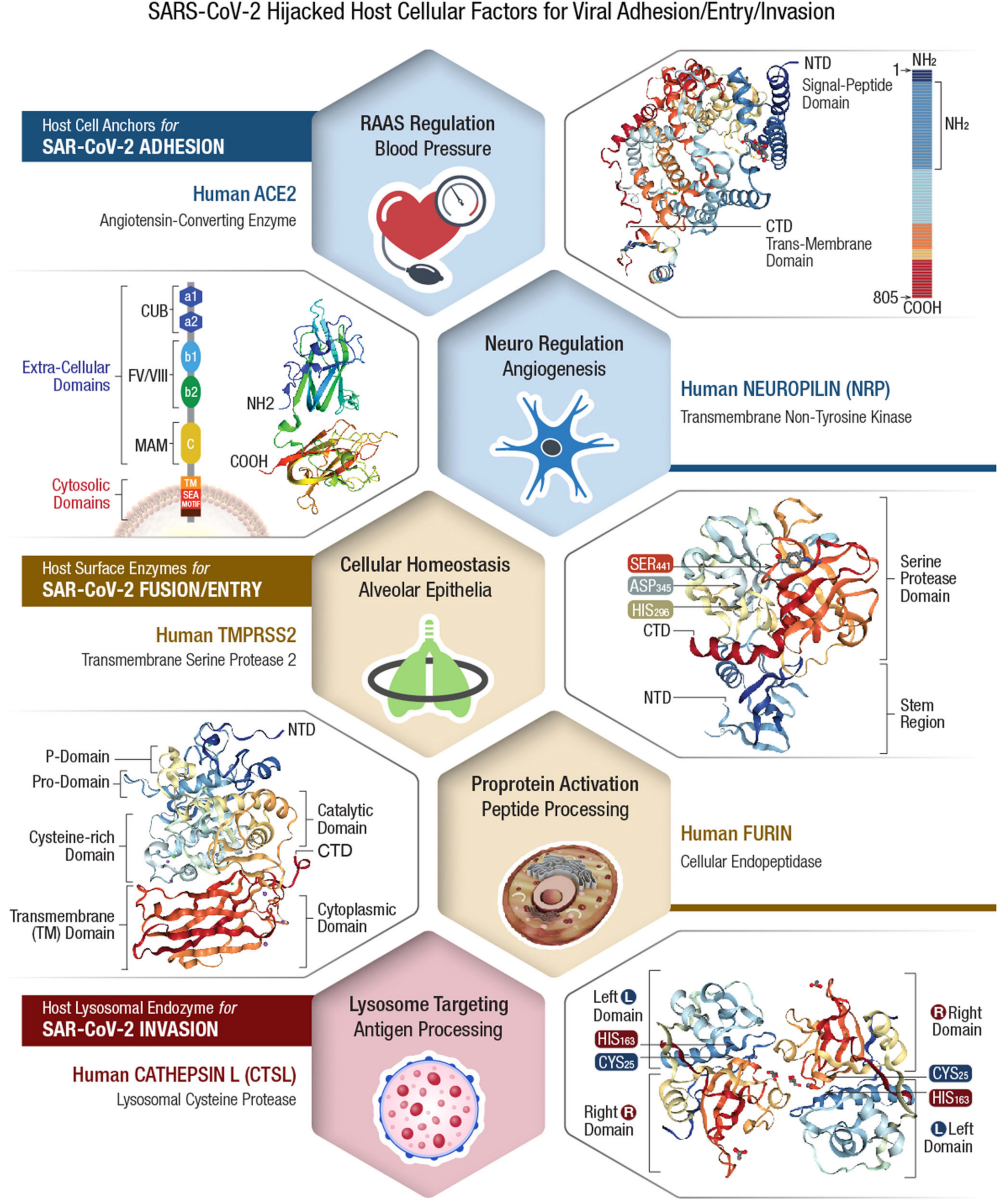
Viral-hijacked host cellular factors and consequences. Clinical outcomes of COVID-19 depend on the ability of SARS-CoV-2 pathogen to hijack host metabolic machinery as well as cellular factors of an infected individual for invasion and internalization, followed by intra-cellular replication to assemble and release multiple viral copies for ultimate propagation/transmission. Each of the viral hijacked host cellular factor is also a quintessential functional component of human metabolism. *Viral host receptor ACE2*, is a critical regulator of blood pressure, controller of blood volume involved in systemic vascular resistance, and in CV homeostasis^292^. *Viral host receptor NRP1*, is vital for several physiological pathways including nervous and vascular development, VEGF-dependent angiogenesis (i.e., new blood vessel formation), immunity and tumorigenesis^321,322^. *Viral membrane fusion priming enzyme furin*, is known for intracellular proteolytic processing of precursor polypeptides, which is an essential step in the maturation of many proteins such as plasma proteins, hormones, neuropeptides, and growth factors^331^. *Viral membrane fusion priming enzyme TMPRSS2* plays a key role in digestion, salt-water balance, iron metabolism, tissue remodeling, blood coagulation, auditory nerve development, and fertility^366^. *Viral endocytosis-mediator* CTSL is involved in functional development of immune system, skeletal physiology including bone collagen degradation/resorption and thyroid hormone release^384,385^. Consequential to the viral hijack, these essential host cellular factors could malfunction and lead to a plethora of organ/system impairments with detrimental consequences to the human body. If not corrected or reset, this HMRD condition may persist in a PASC patient for weeks or months even after viral clearance and recovery from COVID-19.

Clinical outcomes of COVID-19 are directly related to the ability of the SARS-CoV-2 pathogen to hijack host metabolic machinery as well as cellular factors of an infected individual, for viral invasion and internalization, followed by intra-cellular replication to assemble and release multiple viral copies for ultimate propagation/transmission. Each of the SARS-CoV-2 hijacked host cellular factor is also a quintessential functional component of several key physiological pathways of human metabolism. In consequence, the virus-hijacked host cellular factors undergo HMRD, with altered or compromised function, which ultimately contributes to a plethora of organ/system impairments with detrimental effects on human metabolism. If not reversed or reset, the virus-induced HMRD condition may persist as PASC for weeks or months, even after viral clearance and recovery from COVID-19. The following section elaborates on five such critical virus-hijacked host cellular factors exploited for initial steps of SARS-CoV-2 infection, the viral host cell surface adhesion, cellular entry, and intracellular invasion (Fig. 5).

## PASC: viral-hijacked host cellular factors and consequences

The initial step of COVID-19 pathogenesis involves that the viral *spike* (S)-protein interacts and anchors to susceptible host tissue by hijacking of specific CSRs (i.e., ACE2 and NRP1). Subsequent cellular invasion (internalization) of the pathogen takes place as the viral S-protein/host receptor complex is primed by furin cleavage at two sites: S1/S2 and S2’. This proteolytic cleavage induces conformational changes that favors S-protein recognition by host cell membrane proteases (i.e., TMPRSS2, CTSL). The cleavage of S2’ triggers fusion between viral envelope and cell membrane to facilitate SARS-CoV-2 entry into the host cell. The structure-functional properties of these 5 specific viral-hijacked host cellular factors, their ultimate pathophysiological consequence(s) due to virus-induced HMRD in acute COVID-19 and during long-COVID, the persistent virus-free PASC disease state, is comprehensively described below.

### **Viral-hijacked human angiotensin-converting enzyme-2 (hACE2)**

SARS-CoV-2 binds to the human *angiotensin-converting enzyme-2* (hACE2) as a potential CSR for anchoring to specific cellular tissue sites in the body^286–288^. Human cells that express ACE2 are potential targets for SARS-CoV-2 infection; however, other cellular factors such as human proteases that prime the viral S-protein are also critical for the next sequential steps of the viral infection process^55,289^. The hACE2 levels are highest in the small intestine, testis, kidneys, heart, thyroid, and adipose tissue; moderately present in the lungs, colon, liver, bladder, and adrenal glands; lowest in the blood, spleen, bone marrow, brain, blood vessels and muscle^290^. ACE2 exists either in free soluble form (sACE2) or in bound form immobilized on cell membranes (mACE2) of intestinal, renal, testicular, gall bladder, pulmonary, and cardiovascular (CV) epithelia^291^. Both soluble and membrane-bound ACE2 proteins are critical for the regulation of blood pressure in the body.

#### ACE2 structure/function

ACE2 is an essential counter-regulatory *carboxypeptidase* of the hormonal RAAS, a vital regulator of blood volume, systemic vascular resistance, and thus the CV/circulatory homeostasis^292^. ACE2 is expressed in most human tissues and cell types as a *type I integral membrane protein* solubilized by the action of *a disintegrin and metallopeptidase* (ADAM)-17^293^. The human *ACE gene* expresses two distinct isoforms, the *somatic ACE* (sACE) and *testicular ACE* (tACE)^294^. The tACE form is exclusive to the male germinal cells with an enzymatic activity critical for male fertility^294–296^. The other isoform, sACE, is widely distributed on the surface of endothelia, neuroepithelia, and immune cell cascade^297^. Besides these two isoforms, humans express an ACE homolog—the ACE2^298,299^. ACE2 enzyme degrades ANG II, the major effector of the RAAS that increases hypertension (by lowering baroreceptor sensitivity) to control heart rate, up-regulate vasoconstriction, sodium retention, OxS, inflammation, and fibrosis^300^. Therefore, hijacking hACE2 may shift RAAS homeostasis and compromise CV function^301^. ACE2 was also identified as a potential receptor for the cellular entry of several hCoVs, including HCoV-NL63, SARS-CoV, and SARS-CoV-2^302,303^.

#### Consequences of ACE2 hijack

The viral hijack of hACE2 could compromise patient’s health for extended periods of time, especially among COVID-19 and PASC with comorbidities (i.e., CVD, T2DM, brain and kidney dysfunctions)^300,304^. The human ACE2 gene is strongly associated with diabetes; therefore, any loss of hACE2 decreases insulin secretion and impairs the glucose tolerance^305,306^. This partly explains the higher morbidity and mortality rates observed in COVID-19 patients with preexisting diabetes^307^. Human ACE2 expression is high in tubular epithelia (from kidney), and the viral hijack of this enzyme could alter sodium transport, affect blood volume/pressure and lead to AKI^308^. Viral hijack of hACE2 in the BBB axis could impair *autonomic nervous system* (ANS) and dysregulate blood pressure and respiration^76^. Viral hijack of hACE2 in the brain stem may increase sympathetic nerve drive, alter baroreflex, and exacerbate hypertension^309^. Loss of hACE2 in the vasculature may lead to endothelial dysfunction, inflammation and aggravate atherosclerosis and diabetes^284,310^. A loss of pulmonary hACE2 may cause hypertension, respiratory distress, and fibrosis post-viral infection^311^. Thus, SARS-CoV-2-mediated cell surface reduction of hACE2 receptors could trigger widespread inflammatory sequelae observed in COVID-19, which may linger through long-COVID for an extended period of time.

### Viral-hijacked human neuropilin (hNRP)-1

SARS-CoV-2 anchors to human ACE2 as its primary host CSR; however, the broad-spectrum tissue tropism of COVID-19 raises the possible involvement of other host receptors in the binding of SARS-CoV-2 to other target tissue sites. *Neuropilin-1* (NRP1) is another prominent CSR that facilitates entry of SARS-CoV-2 into the CNS through olfactory epithelium in the nasal cavity^63^. Also, NRP1 (but not ACE2) serves as the principal CSR to mediate SARS-CoV-2 infection of astrocytes in the brain tissue. Viral infection of astrocytes resembles reactive astrogliosis with elevated type-I IFN production, increased inflammation, and down-regulation of transporters for water, ions, choline, and neurotransmitters^312^. These events lead to dysfunction and death of uninfected bystander neurons that could inflict severe symptoms of neuro-COVID including anosmia, ageusia, headache, delirium, acute psychosis, seizures, and stroke^171,197^.

NRP1 avidly binds to furin-cleaved S1 fragment of viral S-protein and potentiates cellular entry of SARS-CoV-2^63^. NRP1 is abundantly expressed in the respiratory and olfactory epithelia, with the highest localization in endothelial and epithelial cells. Blocking of NRP1/S-protein interaction with small-molecule inhibitors or monoclonal antibodies (mAbs) may reduce SARS-CoV-2 infectivity and provide potential intervention strategies for COVID-19 management^62^.

#### NRP1 structure/function

NRPs are single-pass transmembrane, non-tyrosine kinase surface glycoproteins, expressed by endothelial, immune, and vascular smooth muscle cells and are regulators of numerous signaling pathways within the vasculature^313,314^. Two homologous NRP isoforms are known to exist, namely NRP1 and NRP2, encoded by distinct *neuropilin* genes (*Nrp1* and *Nrp2*)^315–317^. Both NRPs originally identified as neuronal adhesion molecules, participate in *Semaphorin*-mediated axonal guidance^318^. They are also expressed in vascular and lymphatic endothelia, affecting proliferation, migration, angiogenesis, as well as the formation of small lymphatic vessels and capillaries^313^. NRP1 plays a key role in VEGF-dependent angiogenesis (i.e., new blood vessel formation)^319^. NRP2 is important for migration, antigen presentation, phagocytosis and cell–cell contact in the immune system^320^. Both NRPs play a multifunctional role in several physiological pathways including nervous and vascular development, as well as in immunity and tumorigenesis^321,322^.

#### Consequences of NRP1 hijack

The SARS-CoV-2 S-protein could hijack NRP1 signaling and directly affect VEGF-A-mediated pain. This may raise the possibility that pain, an early symptom of COVID-19, could be diminished by the SARS-CoV-2 S-protein interaction with NRP1^323^. Such ‘silencing’ of pain through subversion of VEGF-A/NRP-1 signaling could be an underlying factor for disease transmission through SARS-CoV-2 infected asymptomatic or minimally symptomatic individuals^324^. As a key player in VEGF-induced vascular permeability and angiogenesis, the viral hijack of NRP1 could impede capillary formation, tissue repair, and organ function in the body^319^. Furthermore, loss of NRP1 could compromise the integrity of vascular endothelium, a selective barrier that regulates macromolecular exchange between the blood and tissues. The ensuing vascular hyper-permeability of plasma molecules and leukocytes may lead to acute tissue edema and inflammation^325^. SARS-CoV-2 infection of astrocytes via NRP1 hijack could disrupt normal neuron function, induce neuronal cell death leading to abnormal manifestations in the CNS^312^. NRP1 deficiency in visceral smooth muscle cells could negatively impact GI contractility and motility^326^. Also, the viral hijack-mediated suppression of epithelial NRP1 could weaken the gut barrier function^327^. The NRP-1-mediated SARS‐CoV‐2 entry into *bone marrow‐derived macrophages* (BMM) could impede osteoclast differentiation and affect calcium/phosphorus metabolism in COVID‐19 patients^328^. Accordingly, viral hijacking of NRP1 could exert chronic disorders of bone metabolism such as osteoporosis or osteopetrosis in PASC patients^116,117^.

### Viral-Hijacked Human Furin

SARS-CoV-2, the etiological agent of COVID-19 pandemic, contains a unique insertion of amino acids (AAs), exclusively *proline-arginine-arginine-alanine-arginine* (P**R**RA**R**_685_ ↓ ) at the S1/S2 boundary of its S-protein, which is clearly absent in SARS-CoV and other related hCoVs^72^. Interestingly, this P**R**RA**R** insertion generates a ‘furin cleavage site’ on S-protein at the S1/S2 multi-basic region, considered as a potential ‘gain of function’ for the viral pathogen. Furin-mediated priming of viral S protein at S1/S2 (P**R**RA**R**_685_ ↓ ) [the underlined AAs refer to critical residues needed for the furin recognition] is a key determinant in the pathogenesis of COVID-19^55,73,329^.

Furin is a member of the *proprotein convertase* (PC) family of enzymes, known to process latent precursor proteins into their biologically active state^330^. Intracellular proteolytic processing of precursor polypeptides is an essential step in the maturation of many proteins such as plasma proteins, hormones, neuropeptides, and growth factors^331^. Due to their homology with bacterial *subtilisin* and yeast *kexin* proteases, PCs are also known as the *PC subtilisin/kexin type* (PCSK) enzymes^330^. Humans encode nine members of the PCSK family (from 1–9), where the PCSK3 represents ‘furin’^332^. Most viral envelope glycoproteins, like bacterial exotoxins, also require proteolytic cleavage to mediate entry into host cells. Accordingly, viral pathogens hijack cellular endo-proteases, such as furin/PCSK3 that prime polybasic cleavage sites and provide critical access for tissue tropism and viral spread in an infected host^333^. Accordingly, the canonical polybasic ‘furin cleavage site’ has been reported in several enveloped viruses, including *Herpes-, Corona-, Flavi-, Toga-, Borna-, Bunya-, Filo-, Orthomyxo-, Paramyxo-, Pneumo*, and *Retroviridae*^332^.

#### Furin structure/function

Furin is a 794-AA type-1 *transmembrane* (TM) protein with large luminal and extracellular regions, a common feature among PC enzymes. Furin is ubiquitously expressed; however, its mRNA and protein levels vary depending on the cell type and tissue. Furin levels are high in salivary glands, liver, and bone marrow, whereas its expression is relatively low in muscle cells^334^. Furin/PCSK3 cleaves basic AA motifs; therefore, also termed as PACE *(Paired basic AA Cleaving Enzyme)*. It cleaves diverse types of protein precursors in the secretory pathway at downstream of basic-AA target sequence (canonically, R-X(R/K)-R)^335^. Furin most likely cleaves and activates more than 150 mammalian, viral and bacterial substrates. These include viral envelope glycoproteins and bacterial toxins, as well as cellular factors that promote tumorigenesis^336^. Substrates for furin cleavage possess a specific 20-residue recognition sequence motif, which includes: pro-*parathyroid hormone* (PTH), *transforming growth factor* (TGF)-β1 precursor, *pro*-*albumin*, membrane type-1 *matrix metalloproteinase* (MMP), β-subunit of pro-*nerve growth factor*, and *von Willebrand factor*^337^.

Several bacterial and viral pathogens exploit human furin enzymes for proteolytic activation of their own virulent factors during the infectious process^332^. In bacterial pathogens, furin-activated toxins may promote tissue invasion, increase transmission rates, or suppress cellular immune responses^338^. The presence of ‘furin cleavage site’ in diphtheria toxin (R-V-R-R ↓ ) and *Pseudomonas* exotoxin A (R-Q-P-R ↓ ), enhances their cytotoxic spectrum^339,340^. Similarly, furin could cleave shiga and shiga-like toxins of certain *Shigella* spp. and *Escherichia coli* to enhance their ability to inhibit host protein synthesis^341^. Thus, without the proteolytic activation of certain bacterial exotoxins, diseases such as dysentery or diphtheria would not occur. In anthrax toxin, these three sub-unit proteins consist of a receptor-binding *protective antigen* (PA), an enzyme-active edema factor and a lethal factor^342^. Furin cleavage of PA results in its oligomerization at the cell surface into a pre-pore that facilitates membrane interactions with edema factor and lethal factor. Subsequently, the furin-activated anthrax toxin complex is endocytosed into the cytoplasm and elicits lethal outcomes^341,343^.

#### Consequences of furin hijack

Furin is essential for cardiovascular (CV) function; therefore, viral hijack of this proprotein convertase enzyme could alter lipid metabolism, and affect blood pressure regulation and vascular remodeling in COVID-19 patients^344,345^. Furin also regulates the transcription factor NKX2-5, which is essential for both normal heart development and CV function^346^. Viral hijack of furin may increase the susceptibility of tissue epithelia for ferroptosis-like cell injury^347^. Ferroptosis is a type of cell death linked to altered iron metabolism, *glutathione* (GSH) depletion, *GSH peroxidase 4* (GPX4) inactivation, and increased OxS, which is a prominent clinical feature of COVID-19^27,348^. Furin is an integral part of specialized cellular machinery that regulates *membrane type-1 matrix metalloproteinases (MT1-MMPs)* and its deterrence by SARS-CoV-2 could compromise proteolytic events on host cell surface and affect nutrient transport for cellular metabolism^349^. Loss of furin due to viral pre-utilization may result in increased growth, invasiveness and cytokine production in the bone-joint synovium and aggravate rheumatoid arthritis^350^. Dysregulation of furin may lead to neurodegenerative and neuropsychiatric disorders known to persist during PASC^171,351^. Co-existence of furin with ACE2/RAAS in the female and male reproductive systems pose a potential risk on human fertility in COVID-19 patients. Viral hijacking of host cellular factors in the female reproductive tract may disrupt ovarian function and thereby the oocyte quality. Higher expression of ACE2 in the endometrium with age and during the secretory phase raises concern about increased susceptibility to SARS-CoV-2 infection^352^. Furin also regulates placenta-specific (PS)-1-mediated oocyte meiosis and fertilization^353^. Furin plays a major role in oocyte development beyond the early secondary follicle stage and any virus-mediated loss of its proteolytic activity could lead to follicular dysplasia and female infertility^354^.

### Viral-hijacked human transmembrane protease, serine 2 (TMPRSS2)

Human *transmembrane protease, serine 2* (TMPRSS2) has been identified as a key host cell factor that determines the route of viral entry for SARS-CoV-2 infection and the pathogenic spectrum of COVID-19^355^. Specifically, TMPRSS2 processes the SARS-CoV-2 S-protein and enables the viral entry into host cells within <10 min in a pH-independent manner. In TMPRSS2-defecient cellular tissue sites, SARS-CoV-2 is endocytosed into lysosomes, and an alternative route of viral entry into the cytosol is achieved in about 40–60 min of post-infection via acid-activated CTSL protease^356^. TMPRSS2 cleaves the viral S protein at multiple sites, including the canonical S1/S2 cleavage site^355^. *TMPRSS2* expression is high in the human prostate gland under androgenic hormone regulation. As an apical surface serine protease, TMPRSS2 regulates epithelial sodium homeostasis in the prostate gland and plays a vital role in male reproduction^357^. In normal prostate, TMPRSS2 is involved with proteolytic cascades to activate *prostate-specific antigen* (PSA) in the seminal fluid (like fibrinolytic blood coagulation)^358^. TMPRSS2 expression is high in ciliated cells and *type I alveolar epithelia* (AT1), which is known to upregulate with ageing^359^. This may explain the relative protection of infants and children from severe respiratory illnesses. Recently, both TMPRSS2 and ACE2 were detected in human corneal epithelia, which suggests that the ocular surface is a potential route of cellular entry for SARS-CoV-2^360^.

#### TMPRSS2 structure/function

TMPRSS2 is a 492 AA polypeptide composed of an intracellular single-pass TM domain, and a bioactive ectodomain with three functional subunits: (i) N-terminal *low-density lipoprotein (LDL) receptor type-A* (LDLR-A) domain, which is a Ca^2+^ binding site; (ii) class-A *scavenger receptor cysteine-rich* (SRCR) domain, which binds to other cell surface or extracellular molecules; and (iii) C-terminal trypsin-like *serine peptidase* (SP) domain with a canonical *His*_*296*_*-Asp*_*345*_*-Ser*_*441*_ catalytic triad for proteolytic activity to cleave *Arg* or *Lys* residues^357,360^.

TMPRSS2 belongs to the *type 2 transmembrane serine protease* (TTSP) family comprising of 19 surface-bound trypsin-like serine proteases. TTSPs initiate several pericellular proteolytic pathways vital for degradative remodeling of extracellular matrix, proteolytic activation of membrane proteins, and putative epithelial homeostasis^361–364^. Human TMPRSS2 mRNA is expressed in many tissues, including prostate, breast, bile duct, kidney, colon, small intestine, pancreas, ovary, salivary gland, stomach, and lungs^365^. *TMPRSS2* plays a vital role in several biological functions such as digestion, salt-water balance, iron metabolism, tissue remodeling, blood coagulation, auditory nerve development, and fertility^366^. It is also required for many pathobiological pathways that involve inflammatory responses, tumor cell invasion, apoptosis, and pain^367^. TMPRSS2 modulates dendritic cells and regulates cytokine release, a major clinical manifestation in COVID-19 pathology^368^.

#### Consequences of TMPRSS2 hijack

As a membrane-anchored enzyme of the host cellular machinery, TMPRSS2 activates precursor molecules in the pericellular milieu to establish metabolic homeostasis^362^. Viral hijacking of this protease could dysregulate lipid metabolism, adipose tissue phenotype, and thermogenesis via direct growth factor activation or indirect hormonal mechanisms^366^. TMPRSS2 expression is high in human prostate gland and this enzyme regulates sperm function in the seminal prostasome^357^. Increasing evidence suggests that COVID-19 could inflict detrimental effects on spermatogenesis and hormonal regulation in male patients^369^. Abnormal serum *follicle-stimulating hormone* (FSH), *luteinizing hormone* (LH), and *testosterone* (T) levels were also reported, which suggests a dysfunctional *hypothalamic-pituitary-gonadal* (HPG) axis in COVID-19 patients^370^. These male reproductive health issues may aggravate and continue to linger in PASC patients. Dysregulation of TMPRSS2 expression and/or catalytic activity may cause both tumor formation and metastasis, contributing to the etiology of several cancer types, especially prostate cancer^371^. Interestingly, TMPRSS2 is reportedly associated with tumor cell expression, different complex(es) formation, and pathways, as well as transcriptional mis-regulation in prostate cancer among COVID-19 and PASC patients^372^.

### Viral-hijacked human cathepsin L (hCTSL)

In the pathogenesis of COVID-19, the cleavage and priming of S-protein is critical for viral entry into host cells. SARS-CoV-2 uses different routes of host cell entry: i) membrane fusion (with cells that express both ACE2 + serine proteases i.e., TMPRSS2 and furin) and/or ii) receptor-mediated endocytosis (to target cells that express only ACE2 + cysteine proteases i.e., CTSL)^74,373^. Interestingly, CTSL alone could activate membrane fusion of viral S-protein and facilitate host cellular entry of the virus^374^. Therefore, viral hijack of CTSL provides an alternative entry mechanism (via endo/lysosomal route) for SARS-CoV-2 invasion of host cells that lack TMPRSS2 enzyme^375^. The activated/primed S protein further mediates fusion of viral envelope with host cell membrane and releases the SARS-CoV genome into the cytoplasm for subsequent viral expression/replication. The CTSL expression is up-regulated during chronic inflammation and is involved in the degradation of the extracellular matrix, an important process for SARS-CoV-2 to enter host cells^376^. Furthermore, the circulating level of CTSL is elevated after SARS-CoV-2 infection and positively correlates with the disease course/severity of COVID-19^78^. The SARS-CoV-2 *Omicron* variant, which recently dominated the pandemic, prefers the endo/lysosomal cysteine protease CSTL over TMPRSS2 for host cell entry^377^. Inhibition of CTSL is therefore, considered an effective strategy to minimize internalization of the virus.

*Cathepsin L* (CTSL), a member of the lysosomal cysteine protease family, shares a catalytic mechanism and sequence homology with non-specific plant protease, *papain*^378^. CTSL contains lysosomal targeting motifs with maximal catalytic activity at acidic pH (3.0–6.5) in the presence of *thiol* (-SH) compounds. The enzyme activity and stability of CTSL at physiological pH strictly depend on the ionic strength of the milieu^379^. The endopeptidase activity of CTSL generates active enzymes, receptors, transcription factors, and biologically active peptides by limited proteolysis^380^. Limited endosomal proteolytic activity of CTSL is critical for diverse cellular processes such as normal lysosome-mediated protein turnover, antigen/proprotein processing, regulation of signaling molecules, extracellular matrix remodeling, and apoptosis^381,382^. CTSL plays a vital role in the functional development of the immune system^383^, in skeletal physiology including bone collagen degradation/resorption and thyroid hormone release^384,385^. Human cysteine proteases are involved in pathogenesis of several diseases including rheumatoid arthritis, osteoporosis, tumor metastasis, renal diseases, diabetes, periodontal diseases, and viral infections^376,386^.

#### Consequences of CTSL hijack

In the cytosol and nuclei, CTSL is critical for several biological pathways, including cell division^387^. CTSL regulates oocyte maturation and early embryonic divisions. Any interference with CTSL activity could impair female competence for embryonic development^388^. Accordingly, the viral hijack of CTSL may reduce female competence for embryonic development (also a major cause of infertility) and may account for early miscarriages during COVID-19 pandemic^389^. Furthermore, several lysosomal enzymes are involved in female ovulation, especially CTSL, in ovarian follicle growth and maturation^390^. The CTSL-mediated activation of progesterone receptors in granulosa degrades extracellular matrix in the follicular tissue during female ovulation^391^. Viral hijack of CTSL could severely compromise female reproductive health.

In secretory vesicles, CTSL generates active neuropeptides including enkephalin, β-endorphin, and dynorphin, as well as *proopiomelanocortin* (POMC)-derived peptide hormones *adreno-corticotropin hormone* (ACTH), and *melanocyte stimulating hormone* (MSH), are essential for cell-cell communication in the nervous and endocrine systems^392^. CTSL also converts proenkephalin into the active enkephalin, an opioid peptide neurotransmitter that mediates pain relief^393^. Inhibition of CTSL alleviates microglia-mediated neuroinflammatory responses from *caspase-8* and NF-κB pathways^394^. A majority of COVID-19 and PASC patients show neurological symptoms, including headache, impairment of memory, seizures, and encephalopathy, as well as anatomical abnormalities, such as changes in brain morphology^201,395^. The viral hijack of CTSL could be a contributing factor for these cognitive dysfunctions in SARS-CoV-2 infection.

In addition to cardiac injury (myocardial infarction, fulminant myocarditis, arrhythmias, venous thromboembolism, and cardiomyopathies), the vasculature is severely affected in COVID-19 and PASC, directly by the SARS-CoV-2 hijack of host factors, and indirectly from the systemic inflammatory cytokine storm^396^. Senescence of vascular endothelium is a hallmark of vascular aging, which leads to the initiation, progression, and advancement of CVD. CTSL plays a key role in vasculo-endothelial senescence via regulation of AKT/ ERK1/2-P21 pathway^397^. Lysosomal CTSL attenuates cardiac hypertrophy and preserves cardiac function by facilitating autophagy and proteasomal protein processing^398^. Hypertension is another independent prognostic factor of poor clinical outcomes in elderly COVID-19 patients^399^. The development of hypertension involves extensive arterial wall remodeling, in which CTSL plays an essential role. CTSL regulates tissue inflammatory responses and extracellular matrix accumulation, thereby preventing arterial remodeling and hypertension, in part by inhibition of smooth muscle cell proliferation in the vessel wall^400^. CTSL is also involved in inflammation and remodeling of vascular as well as extracellular matrix, the cardinal pathological events in systemic sclerosis. Loss in dermal CTSL expression may lead to dermal fibrosis in systemic sclerosis^401,402^. Thus, CTSL hijack may have detrimental consequences on CV health in COVID-19 and PASC patients.

Obesity, diabetes, and other related metabolic syndrome pose a higher risk of severe COVID-19 infection with poor prognosis^403^. CTSL is known to degrade fibronectin, *insulin receptor* (IR), and *insulin-like growth factor-1 receptor* (IGF-1R), essential molecules for adipogenesis and glucose metabolism. Inhibition of CTSL results in reduced body weight, low serum insulin levels, and increased glucose tolerance^404^. New onset T2DM, arterial hypertension and dyslipidemia are possible sequelae of COVID-19 infection^405^. Viral hijack of CSTL in obese and diabetic COVID-19 patients suggest that this protease is a novel target for new-onset metabolic disorders in PASC patients.

## Long-COVID/PASC: precision nutrition to reset virus-induced HMRD

Viral hijacking of host metabolic machinery by SARS-CoV-2 genome and its expressed proteins is critical for viral biogenesis and propagation^21^. During the pathogenesis, SARS-CoV-2 reprograms and dysregulates several host cellular pathways that are involved in metabolism, bioenergetics, iron-redox signaling, and immunity—collectively termed as HMRD^22,27^. The pathophysiological onset and persistence HMRD in COVID-19 and PASC patients is a cumulative outcome of metabolic remodeling culminating from both SARS-CoV-2-induced cellular damage as well as host antiviral responses. Extensive clinical data combined with genomic and metabolomic profiles has revealed several acute as well as chronic physio-chemical vulnerabilities both among SARS-CoV-2 infected COVID-19 cases as well as virus-free (RT-PCR negative) PASC patients. SARS-CoV-2-induced HMRD triggers viral pathogenesis by re-directing free AAs and FAs from host cellular metabolism, as building blocks to support viral progeny and propagation. Therefore, precision nutrition to ‘reset’ (or reverse) HMRD is a functional strategy to combat both COVID-19 and PASC. Thus, an ideal nutritional remedy needs to demonstrate human *randomized clinical trial* (RCT)-proven health benefits with optimal ADME (*Administration, Distribution, Metabolism, Excretion*) profile and functional efficacy to reset HMRD and facilitate total recovery of PASC patients^22^. Accordingly, such precision nutrition protocols consisting of specific bio-functional compounds to reset or resolve SARS-CoV-2-induced HMRD are shown in Table 1.

**Table 1:** Precision nutrition categories to reset or resolve SARS-CoV-2-induced HMR/D

Precision nutrition category	RCTs	
	Done	Total
*Fe-redox dysregulation*		
Hypoxia		
L-arginine	4	6
L-tryptophan	1	1
Fe-redox regulators		
Lactoferrin (LF)	2	13
Hemoxygenase-1 (HO-1)	4	6
Erythropoietin (EPO)	3	4
Hepcidin/HEP modulators	4	6
Ferroptosis inhibitors		
Vitamin E	3	5
Ferrostatin (FER-1)	–	–
Dietary phytochemicals		
Quercetin	1	4
Glycyrrhizin/liver disease	7	15
Curcumin	1	2
*Altered bioenergetic systems*		
Mitochondrial dysfunction (*m*-Dys)		
Nicotinamide adenine dinucleotide (NAD)	3	6
Alpha-lipoic acid (ALA)	-	1
Coenzyme Q10 (CoQ10)	3	6
Creatine	2	3
Vitamin B12 (cobalamine)	2	7
Oxidative stress (OxS)		
Superoxide dismutase (SOD)	1	2
Catalase (CAT)	1	2
Glutathione (GSH)	2	8
* N*-acetyl-cysteine (NAC)	2	8
Glutamine	–	–
Maillard reaction products (MRP)	–	–
*Viral hijacked host factors*		
ACE2/RAAS		
L-carnitine	1	3
NRP1		
Melatonin	4	6
Serine protease		
Flavone-3-OL	–	–
Sulforaphane	–	–
*New ‘onset’ disorders*		
Immune impairment		
Vitamin D3 (cholecalciferol)	23	44
Omega-3/PUFAs	3	3
Vitamin C (ascorbic acid)	15	34
Dysbiosis		
Probiotics/lactic acid bacteria (LAB)	11	17
Metabolic syndromes		
Diabetes (T2DM)	–	–
Obesity (hyperlipemia)	–	–

Specific dietary-based ‘reset’ interventions from registered randomized controlled trials (https://ClinicalTrials.gov) undergoing efficacy evaluation against COVID-19 and/or PASC are listed. Each dietary intervention shows the number of RCTs completed and total number of registered RCTs.

### Nutritional reset of hypoxia/’Warburg’ effect

Dysregulation of glucose metabolism with elevated serum glucose levels and upregulation of glycolytic intermediates is a prominent feature of COVID‐19. Glucose metabolism supports OXPHOS/TCA cycle (ATP synthesis) in mitochondria and generates malate, an indicator of mitochondrial activity. Interestingly, plasma malate levels dwindle after SARS‐CoV‐2 infection, which suggests *m*-Dys due to hypoxia/Warburg effect in the pneumonia state^27,51^. The SARS-CoV-2-induced hypoxia activates gluconeogenesis and porphyrin (or iron) metabolism, thereby steers the clinical onset/progression of COVID-19. Furthermore, the hypoxia-mediated ‘Warburg’ effect also alters both TCA cycle as well as lipid metabolism through perturbation of tryptophan biosynthesis, aminoacyl-tRNA degradation, phenylalanine, and arachidonic metabolism^406^. Dysregulated arachidonic acid metabolism and fatty acid β‐oxidation in tandem with platelet aggregation and bile acid synthesis help identify SARS-CoV-2 infected asymptomatic individuals, the hidden drivers of COVID-19 pandemic as well as massive victims of PASC^21,407^.

***l-Arginine*** metabolism is vital for immune and vascular functions in the body^408^. Its metabolic functions include conversion to *nitric oxide* (NO•) by *NO synthase* (NOS) or arginine catabolism to ornithine by arginase^409^. NO• is master regulator of cardiovascular function, metabolism, neurotransmission, and immunity^410^. Upregulation of arginase depletes serum levels of arginine, subsequently inhibits NO• production. Accordingly, low availability of plasma arginine has been implicated in endothelial dysfunction, T cell dysregulation, and thrombocytopenia (coagulopathy) in ARDS patients, the most severe form of COVID-19^408,411^. Thus, restoring optimum levels of arginine through oral supplementation could maintain immune homeostasis, particularly to reverse altered T cell activity and reset T cell/macrophage functions in PASC patients^412,413^.

Perturbations in l-arginine metabolism is prevalent among COVID-19 and PASC patients, across all disease stages^414,415^. Oral supplementation of l-arginine (1.66 g twice a day) for 10 days in COVID-19 patients (*n* = 101) show significant reduction both in respiratory/ventilation support (71.1%) and in-hospital stay^416^. Oral supplementation with l-arginine (1.66 g/d), in a human RCT (*n* = 110) during COVID-19 pandemic showed patient improvement in cardiac rehabilitation after myocardial infarction and cardiac revascularization^417^. Interestingly, arginine depletion with arginine-metabolizing enzymes has also been suggested as a therapeutic approach to block viral proliferation during acute COVID-19^418^. However, the persistent hyper-inflammatory state and immune dysregulation which is common among virus-free PASC patients, arginine supplementation may prove beneficial to this group. In PASC pathology, the markers of NO• bioavailability are low; accordingly, oral supplementation of these patients with l-arginine + vitamin-C for 4 weeks could significantly elevate serum l-arginine levels and thereby increase NO• bioavailability^419^. In another human RCT (*n* = 50), oral supplementation of l-arginine (1.66 g/d) + vit-C (500 mg/d) for 4 weeks restored physical performance, endothelial function, fatigue, and relieved persistent symptoms in PASC patients^415^. Oral supplementation of l-arginine could be a promising nutritional remedial to reset HMRD-associated cardiovascular disorders and immune dysfunction in PASC patients.

**Fig. 6:**
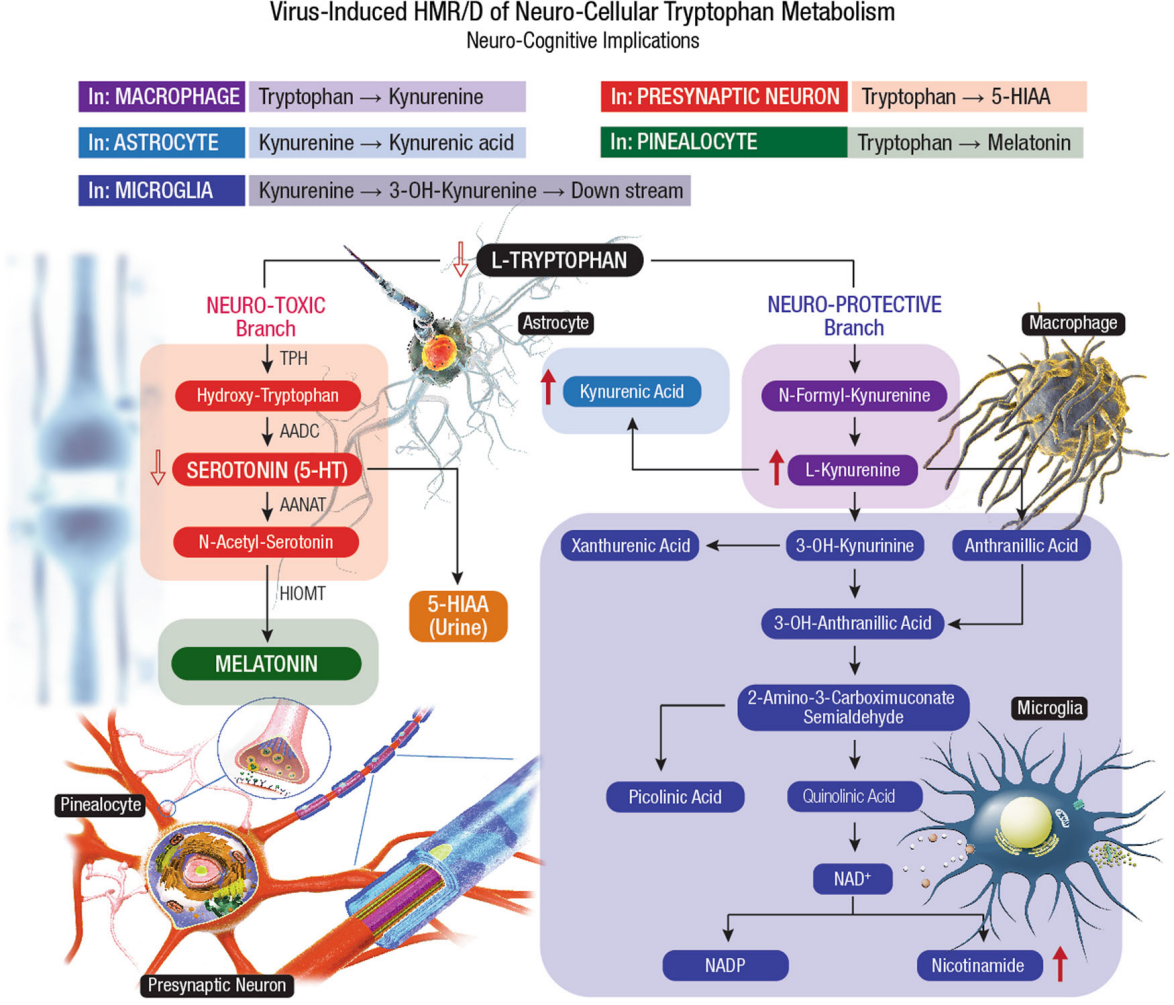
Virus-induced HMRD of tryptophan metabolism/neuro-cognitive implications. The SARS-CoV-2-induced HMR affects tryptophan metabolism by lowering the levels of tryptophan, serotonin, and indole-pyruvate, while elevating the levels of kynurenine, kynurenic acid, picolinic acid, and nicotinic acid^51,424^. After conversion to kynurenine, the tryptophan catabolism divides into different branches, leading to the formation of 3-OH-kynurenine, anthranilic acid or kynurenic acid. The 3-OH-kynurenine catabolism further leads to the generation of picolinic acid, quinolinic acid, and nicotinamide. The neuroprotective kynurenic acid is present mainly in astrocytes, neurotoxic 3-OH-kynurenine and excitotoxic quinolinic acid are found in microglial cells. Besides directly targeting neurotransmitter receptors, the tryptophan metabolites, in particular 3-OH-kynurenine and 3-OH-anthranilic acid, are redox active that impact brain physiology^789^. The modulation of the tryptophan-kynurenine pathway is an indicator for a coherent metabolic shift^790^. The tryptophan-nicotinamide pathway is associated with inflammatory signals and coordinator of cell metabolism in SARS-CoV-2 infection^791^. The broader virulence spectrum of SARS-CoV-2 with ability to cross the BBB and inflict a plethora of neuropathological manifestations by HMRD in host brain metabolism has been elucidated as ‘Neuro-COVID-19’^171^.

***l-Tryptophan*** metabolism is the most prominent pathway affected by HMRD during SARS-CoV-2 infection. The virus-induced re-wiring of l-tryptophan metabolism alters kynurenine pathway, dysregulates host inflammatory and immune responses^420^. The l-tryptophan-kynurenine pathway is a regulatory ‘hub’ for canonical and non-canonical transcription, macrophage release of cytokines, which could trigger hyper-inflammation and cause poor clinical outcomes in COVID-19 and PASC^421^. Major clinical manifestations in PASC such as depression, fatigue, sleep disturbances, attention disorders, anxiety, muscle weakness, and dyspnea are directly linked to ‘kynurenine shunt’, which is known to increase l-tryptophan degradation towards kynurenine and away from serotonin synthesis^422–424^.

l-tryptophan is an essential AA, vital for biosynthesis of neurotransmitter serotonin (*5-hydroxytryptamine*, 5-HT), sleep hormone melatonin and co-factor NAD^+^ through its downstream metabolic pathways^425^. l-tryptophan catabolism by *indoleamine-dioxygenase* (IDO) through the kynurenine pathway generates several bioactive metabolites collectively referred to as kynurenines^426^. SARS-CoV-2 infection could deplete plasma l-tryptophan levels and increase IDO-stimulated generation of neuroactive tryptophan catabolites, including kynurenine^427^. Kynurenine is precursor for the vital cellular effector NAD^+^ and for several other intermediate metabolites that modulate immune and neuronal functions. SARS-CoV-2 infection deprives the host for NAD^+^ by inhibiting the biosynthetic pathway from quinolinic acid, and simultaneously acquiring NAD^+^ from nicotinamide in a salvage pathway^428^. Thus, l-tryptophan metabolism via kynurenine pathway produces *niacin* (vit-B3), a building block for NAD^+^ synthesis. In oxidized form, NAD^+^ is a potent inhibitor of pro-inflammatory cytokines and ventilator-induced *acute lung injury* (ALI) in COVID-19^429^. Accordingly, reduced serum levels of niacin or NAD^+^ reflects l-tryptophan deficiency and increased severity of COVID-19^430^. Activation of kynurenine pathway in elderly COVID-19 and PASC patients is a major cause for cerebrovascular damage^431^. Virus-induced HMRD of tryptophan metabolism and its clinical implication on neuro-cognitive function(s) is shown in Fig. 6.

*Serotonin* (5-HT) is a precursor for melatonin, a chrono-biotic pineal hormone, which may elicit potential adjuvant effects to combat COVID-19 and PASC^432^. Melatonin could also reverse aerobic glycolysis in immune cells and inhibit SARS-CoV-2-induced hyper-inflammatory response in COVID-19 patients^433^. l-tryptophan deficiency could deplete melatonin levels and aggravate pathophysiological risks of COVID-19 and PASC. l-tryptophan deficiency could also lower serum levels of 5-HT and augment disease manifestations such as anosmia, ageusia, and dysfunctional chemesthesis in COVID-19 and PASC^434^. Furthermore, 5-HT deficiency could worsen silent hypoxemia, weaken hypoxic pulmonary vasoconstriction, and compromise the recovery of COVID-19 and PASC patients.

The gut-brain’ axis is a bi-directional system that links emotional and cognitive centers of the brain with the peripheral functioning of the GI tract. The serotonergic system forms a diffuse network within the CNS and plays a neuroprotective role while regulating mood and cognition^435^. Based on 11 human RCTs, oral supplementation of l-tryptophan (0.14 to 3 g/d) with regular meal seem to improve the mood of individuals^436^. l-Tryptophan supplementation, especially at ≥1 g is shown to improve sleep quality and resolve insomnia^437^. Nutritional reset of l-tryptophan levels, and optimization of peripheral and central 5-HT levels could help alleviate neuro-cognitive dysfunction in PASC patients.

### Nutritional reset of Iron (Fe)-Redox dysregulation (FeRD)

SARS-CoV-2 mediated *acute lung injury* (ALI) induces death of inflammatory cells with sloughing of alveolar epithelia and damage of pulmonary vasculature with hemolytic consequences^438^. Free heme released during hemolysis could induce pro-inflammatory, pro-oxidative, and pro-thrombotic effects. FeRD is highly prevalent among hospitalized COVID-19 patients. Serum levels of iron and hepcidin are low in COVID-19 patients, whereas *erythropoietin* (EPO) and haptoglobin levels significantly decline in critical and deceased patients^439^. Other biomarkers of iron metabolism (i.e., ferritin, TF, LF, etc.) and Hb could provide risk stratification strategies for COVID-19 management, as initial anemia is strongly linked to increased CFR. Altered iron-redox homeostasis (Fe-RH) with elevated ferritin/TF ratio predicts subsequent insufficient pulmonary oxygenation (with the need for ICU admission) and mechanical ventilation^96^. Serum TF levels decrease within the 1^st^ week of hospitalization in many COVID-19 patients; however, a continuous decline is prominent among subjects with fatal outcomes^440^. Therefore, nutritional strategies to reset FeRD with Fe-redox regulators and ferroptosis inhibitors could be an effective strategy for optimal recovery of COVID-19 patients and in post-recovery management of PASC^94,441^.

### Fe-Redox regulators

In the human body, the total iron content is ~3-g for women and ~4-g for men, distributed in two main forms as heme-iron, mostly found in the Hb, myoglobin, and cytochromes (2 to 2.7-g); and as non-heme-iron, a cofactor for several enzymes^442^. Free iron levels in human body fluids are regulated at <10^–18^ M to avert microbial infections as well as to prevent the precipitation of insoluble ferric hydroxides and the formation of toxic free radicals. Innate Fe-Redox regulators such as *lactoferrin* (LF), *heme oxygenase-1* (HO-1), *erythropoietin* (EPO), and *hepcidin* (HEP) serve as first innate barriers against free radical damage and hyper-immune responses during COVID-19 and PASC^27^. In COVID-19 recovered virus-free PASC patients, the host Fe-RH is disrupted for an extended period; while the Hb, ferritin, and TF levels slowly restore back to normal after onset of initial FeRD, around a median of 122 days after discharge from the hospital^440^.

*Lactoferrin (LF)*, an iron-binding glycoprotein present in milk as well as several body fluids including saliva, tears, nasal secretions, gastric/cerebrospinal/synovial fluids, sperm, vaginal secretions, and neutrophils, is a key component of innate host defense^443^. Due to iron-chelation and iron-transport properties, LF is considered an innate iron regulator with a multifunctional role in scavenging iron-catalyzed free radicals (i.e., ROS, RNS) and maintain Fe-RH in the body^171^. Free radical scavenging mechanisms of LF involve stimulated glycolysis, increased ATP generation to sustain ion gradient with membrane potential and morphology of the cell^444^. Oral supplementation of LF could prevent oxidative damage by heme iron and reverse ferritin-bound iron overload during chronic inflammation and aging^445^. Notably, both OxS and related metabolic syndromes are considered as potential risk factors in COVID-19 pathology^446^. As an innate regulator of Fe-RH, LF could combat OxS at the cellular level, modulate inflammatory responses at the tissue level and play a therapeutic role in clinical management of COVID-19 and PASC^27,447^.

Several studies have elucidated a broad-spectrum antiviral activity for milk LF against SARS-CoV-2^171,448–450^. Breast milk from several positive COVID-19 mothers were found negative for SARS-CoV-2 pathogen^451,452^. The antiviral spectrum of milk LF include (i) direct interaction with the viral protein target(s) and blockade of viral attachment to host target cells^453^; (ii) binding to *heparan sulfate proteoglycans* (HSPGs) on the host cell surface with subsequent inhibition of viral attachment and cell entry^454^; and iii) interference with intracellular trafficking of the virus^455^. Also, LF is a potent anti-inflammatory agent that modulates hepcidin and *ferroportin* (FPN) synthesis through down-regulation of IL-6^456,457^; thereby inhibits intracellular iron overload^442^. Oral administration of 20–30% iron-saturated milk LF (corresponding to 70–84 μg of elemental iron) twice a day could down-regulate IL-6 and restore FPN-mediated iron export from cells to blood in both hepcidin-dependent or independent pathways^456^. LF regulates both pro-inflammatory and anti-inflammatory responses^447^; thereby could prevent viral insult-induced cytokine storm^458^. LF also activates plasminogen that regulates coagulation cascade and antithrombotic activity, a promising clinical intervention for COVID-19 and PASC^22,459,460^.

Apo-LF is a normoxic mimetic of hypoxia that effectively stabilizes redox-sensitive transcription factors HIF-1α and HIF-2α^461^. In hypoxia, such as during the early stages of SARS-CoV-2 infection, these transcription factors could provide synergistic protection through activation of the Keap1/Nrf2 signaling pathway^462^. Also, LF could block HIF-1α activity and provide therapeutic benefits to retinal neuronal cells during neuro-COVID^171,463^. Taken together, milk LF could reverse iron overload and reduce inflammation, both considered as critical factors in the pathogenesis of COVID-19 and PASC; accordingly, LF could serve as a promising all-natural intervention to resolve FeRD and reset virus-induced HMRD in the ongoing new onset global metabolic syndrome.

*Heme Oxygenase-1* (HO-1) also known as the *‘heat shock protein-32’* (hsp32), is an inducible intracellular enzyme upregulated by >100-fold during infections and clinical conditions such as sepsis, renal ischemia-reperfusion injury and *acute lung injury* (ALI)^464^. At the cellular level, HO-1 exists in the endoplasmic reticulum, mitochondria, nucleus, and plasma membrane^45^. HO-1 mediates catalytic breakdown of heme, a potent pro-oxidant and pro-inflammatory molecule^465^. HO-1 regulates Fe-RH and provides cyto-protection via endogenous mechanisms to sustain body’s antioxidant response against OxS^466^. Human HO-1 binds to the SARS-CoV-2 ORF3a protein and inhibits virus-induced inflammation and tissue damage via the NLRP3 pathway^23,467^. The ability of HO-1 to protect against SARS-CoV-2 infection is probably an emergency inducible defense mechanism to ameliorate OxS from heme-released oxidants^27^. Thus, the cyto-protective function of HO-1 is a promising intervention strategy to control SARS-CoV-2 infection and alleviate virus-induced cytokine storm as well as the subsequent lung dysfunction during COVID-19 and PASC^468,469^.

Several natural compounds could modulate HO-1 expression. *Nimbolide*, a limonoid tetranortriterpenoid isolated from neem plant (*Azadirachta indica*) could upregulate the HO-1 enzyme^470^. Phytochemicals such as, the *Resveratrol* (3,4’,5-trihydroxy stilbene) and the *Curcuminoids* (with *α*, *β*-unsaturated carbonyl groups) are potential inducers of HO-1 expression through Nrf2/*antioxidant-responsive element* (ARE) pathway^471,472^. *Quercetin*, a polyphenolic flavonoid found in a variety of fruits and vegetables could induce HO-1 expression via *mitogen-activated protein kinase* (MAPK)/Nrf2 pathway^473^. In response to inflammation and OxS, the activated HO-1 is a powerful down-regulator of pro-coagulant factors to prevent thrombotic events, endothelial injury from vascular inflammation, resolve FeRD and reset HMRD in COVID-19 and PASC patients^27,46^.

*Erythropoietin (EPO)* is a hypoxia-inducible growth factor expressed in various organs and tissues of the body^474^. Erythropoiesis is the single largest consumer of iron, quintessential for *hemoglobin* (Hb) synthesis in the body. Critical as well as deceased COVID-19 patients demonstrate significantly lower serum levels of EPO, haptoglobin, and hepcidin compared to survivors or mild cases^439^. EPO treatment could restore Hb levels, increase *red blood cell* (RBC) count, and improve O_2_ delivery to the tissues, thereby help in the recovery of both COVID-19 and PASC patients^475^.

Overexpression of proinflammatory cytokines during COVID-19 results in cytokine storm, which ultimately leads to the clinical development of ALI and ARDS. Such hyper-inflammatory conditions could trigger NO• release and inhibit EPO synthesis causing *anemia of inflammation* (AI)^476^. Notably, fatal cases of COVID-19 demonstrate 2.5 times lower serum EPO (2.8 vs. 7.1 mU/mL), and 1.24 times lower Hb levels (14.0 vs 17.4 g/dL) compared to survivors^477^. COVID-19 patients also show ground-glass opacities localized to alveoli indicating the presence of ALI^478,479^. In a clinical study, recombinant human (rh)-EPO showed effective attenuation of ARDS symptoms and facilitated recovery from COVID-19 via multiple mechanisms including cytokine modulation, anti-apoptotic effects and leukocyte release from the bone marrow^475^.

EPO could potentially benefit neuro-COVID-19 patients with acute and chronic-progressive downstream sequelae of the CNS and *peripheral nervous system* (PNS)^171,480^. Therapeutic benefits of EPO on COVID-19 patients may include (i) respiratory improvement at several levels including lung, brainstem, spinal cord, and respiratory muscles^481^; (ii) counteract hyperinflammation caused by cytokine storm/inflammasome^482,483^; (iii) neuro-protection and neuro-regeneration in brain and peripheral nervous system^484^. Thus, EPO could be a potential bio-replenishment to reverse FeRD in COVID-19 and help reset HMRD in PASC patients^27,484,485^.

#### Hepcidin (HEP) and HEP-modulators

*Hepcidin (HEP)*, a peptide hormone secreted by the liver, is a master regulator of iron intake and systemic Fe-RH^486^. HEP regulates iron levels by binding to *ferroportin* (FPN), and the FPN/hepcidin regulatory axis that allows precise control of iron at both systemic as well as cellular levels^487^. HEP synthesis in liver is controlled by four pathways: (i) iron store-related regulation, (ii) erythropoietic activity-driven regulation, (iii) inflammation-related regulation, and (iv) mandatory signaling pathway. These regulatory pathways interact with hepatocytes to initiate or inhibit the production of sufficient HEP to regulate Fe-RH^488^. HEP expression calibrates physiological iron levels, inflammatory cues, and iron requirements for erythropoiesis. Circulating factors (LF, TF, cytokines, erythroid regulators) contribute to HEP modulation in different pathological conditions^456,489^.

Several compounds could act as HEP agonists to prevent iron overload from HEP deficiency^490^. *Homocysteine* up-regulates hepcidin expression through the BMP6/SMAD pathway, which suggests a novel approach to reset Fe-RH^491^. *Calcitonin* is a potent inducer of HEP expression, which may provide an interventional strategy to reverse FeRD^492^. *Genistein*, a member of the isoflavone-related estrogen could induce HEP transcription^493^. As potential inducer of HEP expression, phytoestrogens are promising dietary supplements to reduce iron overload and prevent any sequelae of iron-induced toxicities such as hyperferritinemia, coagulopathies and/or thromboembolism, which are prominent clinical manifestations in COVID-19 and PASC^94,144,494,495^. HEP and HEP-modulators are potential candidates to reset FeRD and reverse iron-overload syndromes such as anemia and *chronic kidney disease* (CKD) in COVID-19 and PASC patients^27,496,497^.

### Ferroptosis inhibitors

*Ferroptosis* is an iron-catalyzed, non-apoptotic form of regulated necrosis that causes oxidative lipid damage in cell membranes leading to *m*-Dys^498^. Ferroptosis with characteristic accumulation of oxidized phospholipids (or their breakdown products) in myocardial and renal tissue is responsible for ischemia-reperfusion injury, which is a detrimental factor for cardiac damage and MODS in COVID-19 patients^499^. Ferroptosis is more immunogenic than apoptosis and plays a detrimental role in hyper-inflammation such as the CRS^500^. Accordingly, ferroptosis might serve as a potential treatment target for COVID-19 and PASC management^27,112^. A hallmark of ferroptosis is iron-dependent lipid peroxidation, which could be inhibited by the key ferroptosis regulator Gpx4, free radical trapping antioxidants and ferroptosis-specific inhibitors^501^. *Nuclear factor (erythroid-derived 2)-like 2* (Nrf2) could suppress ferroptosis and resolve cellular Fe-RH in COVID-19 and PASC^502^.

*Vitamin-E (Vit-E)* or α-tocopherol is a fat-soluble antioxidant that prevents ROS formation during lipid oxidation. Vit-E could effectively prevent ferroptosis by reducing the Fe^3+^ center to inactive Fe^2+^ in *lipoxygenase* (LOX), thereby inhibit enzyme activity of LOX. Notably, LOX oxidizes cell membranes via peroxyl (ROO•) radicals and forms lipid hydroperoxides. Vit-E could neutralize ROO• radicals and prevent formation of lipid hydroperoxides. Afterward, GSH, GPX4, and other antioxidant agents may detoxify oxidized lipids and inhibit ferroptosis^503^. A meta-analysis (*n* = 19 studies) of Vit-E supplementation (mean dosage: 384 mg/d) has shown to reduce OxS in hemodialysis patients^504^.

*Ferrostatin-1* (FER-1) (*Ethyl 3-amino-4-cyclohexylamino-benzoate)* is a potent lipophilic free radical scavenger that could reduce cellular accumulation of lipid peroxides and peroxyl radicals^505^. The anti-ferroptotic activity of FER-1 is associated with its ability to scavenge alkoxyl radicals and remain unconsumed while inhibiting iron-dependent lipid peroxidation^506^. During SARS-CoV-2 infection of bronchial epithelia, the inflammatory IL-6 could induce ferroptosis as well as ROS-dependent lipid peroxidation, leading to severe FeRD^507^. A combination of FER-1 and *N-acetylcysteine* (NAC) could reverse ferroptotic effects of IL-6 and help resolve FeRD in COVID-19 and PASC patients^27,508^. Also, chrono-biotic hormone melatonin could effectively reduce ferroptosis through activation of Nrf2 and HO-1 signaling pathways^509^. The strong ability to inhibit ferroptosis and platelet activation, makes melatonin a potential nutritional intervention to treat hemolytic, thrombotic, and thrombocytopenic conditions^510^, which are widespread among COVID-19 and PASC patients.

*Dietary phytochemicals* could serve as natural ferroptosis inhibitors to resolve FeRD-related manifestations in COVID-19 and PASC^27^. *Glycyrrhizin*, the main extract from licorice (*Glycyrrhiza glabra*), is a natural antioxidant, anti-inflammatory, antifibrotic and antiviral agent widely used in the treatment of chronic hepatitis^511^. *Glycyrrhizin* shows significant reduction in the degree of ferroptosis and inhibits OxS and provides anti-ferroptotic liver protection through up-regulation of Nrf2, HO-1 and Gpx4; and down-regulation of *lactate dehydrogenase* (LDH), Fe^2+^, *malondialdehyde* (MDA), and ROS^512^. *Quercetin* (pentahydroxy flavone), a natural flavonoid, could up-regulate the GSH levels and inhibit ferroptosis by reducing MDA and lipid ROS levels in the renal proximal tubular epithelia^513^. Two tannin hydrolysates, *chebulagic acid* and *chebulinic acid*, were identified as potent ferroptosis inhibitors. Their ferroptosis inhibition is mediated by regular antioxidant pathways (ROS scavenging and iron chelation), rather than the redox-based catalytic recycling pathway by FER-1^514^. *Curcumin* could inhibit *myoglobin* (Mb)-induced ferroptosis in renal tubular cells. Curcumin could reduce Mb-mediated inflammation and OxS by inhibiting the TLR4/NF-κB axis and activating the cytoprotective enzyme HO-1^515^. Dietary polyphenols such as *piceatannol* and *astringin* could strongly inhibit ferroptosis via preferential transfer of hydrogen atoms at the 4’-OH position as conventional antioxidants^154,516^.

### Nutritional reset of mitochondrial dysfunction (*m*-Dys)

*Mitochondrial dysfunction* (*m*-Dys) is characterized by a loss of efficiency in the OXPHOS and reductions in the ATP synthesis, a causative mechanism for several metabolic syndromes including PASC. *m*-Dys leads to fatigue (with reduced tolerance to exercise), a common persistent symptomatic feature amongst COVID-19 survivors. Nutritional strategies to resolve *m*-Dys should comprise specific bioactives and cofactors essential for mitochondrial bioenergetic pathways, as well as provide effective free radical (ROS) scavengers to prevent OxS, maintain Fe-RH, and reset virus-induced HMRD.

*Nicotinamide adenine dinucleotide (NAD*^*+*^*)* is a vital cofactor in mitochondrial bioenergetic pathways, including glycolysis, fatty acid β-oxidation, and the TCA cycle^517^. It exists in both oxidized (NAD^+^) and reduced (NADH) forms, the latter is generated by NAD^+^ accepting high-energy electrons from glycolytic and TCA intermediates and acts as a primary electron donor in ATP synthesis to drive mitochondrial OXPHOS^518^. NAD^+^ also regulates non-redox NAD^+^-dependent enzymes such as *poly-ADP-ribose-polymerases* (PARPs) and sirtuins^519^. The macrodomain-containing protein, *nsp3* of SARS-CoV-2 could counteract the host antiviral defenses from PARPs, SARM1, sirtuins and CD38^157^. Therefore, NAD^+^ and NAD^+^-consuming enzymes are critical for immune responses, cellular bioenergetics, and to design antiviral strategies for COVID-19 and PASC.

Furthermore, NAD^+^ plays a key role in several essential cellular processes including DNA repair, immune cell function, senescence, and chromatin remodeling^520^. Cardiac tissue is dense with mitochondria; as one of the most metabolically demanding organs, the heart has the highest NAD^+^ levels. A decline in NAD^+^ metabolism and low tissue levels of NAD^+^ is a common trait among the elderly. Alterations to NAD^+^ metabolism and/or decline in tissue NAD^+^ levels are directly related to *m*-Dys and pathological conditions such as CVD^521^. SARS-CoV-2 infection dysregulates NAD^+^ metabolism, which could manifest *m*-Dys and lead to *chronic fatigue syndrome* (CFS) in PASC patients^522^. Therefore, nutritional reset of *m*-Dys with NAD^+^ or its natural dietary precursors could be effective in improving myocardial bioenergetics and function.

NAD is synthesized de novo from tryptophan or bio-replenished through NAD^+^ precursors such as nicotinic acid, nicotinamide, or nicotinamide riboside, collectively referred to as niacin/B_3_ vitamins. Dietary NAD^+^ could partially resolve SARS-CoV-2-induced dysregulated gene expression and mitochondrial metabolism^523^. NAD supplement could also alleviate intestinal barrier injury by protecting mitochondrial function in gut epithelia^524^. Also, NAD^+^ supplement could directly inhibit PARP-1 and prevent pro-inflammatory cytokines and resolve hyper-activated immune system. Oral administration of NAD^+^ precursors, such as tryptophan, nicotinic acid (niacin), nicotinamide, and *nicotinamide riboside* (NR), could increase tissue NAD^+^ levels^525,526^. Food supplement cocoa flavanol is shown to boost the NAD^+^ and NADH content, stimulate sirtuin metabolism, reduce cellular H_2_O_2_ production as well as OxS, and improve mitochondrial function in PASC patients^527^. Increasing NAD^+^ levels could also stabilize telomeres and help recovery of elderly PASC patients^528^.

*Alpha-lipoic acid (ALA)* is an intracellular redox regulator that reduces OxS, blocks activation of NF-kB and lowers both ADAM17 activity and ACE2 upregulation^529^. ALA upregulates ATP-dependent K^+^ channels in the cell, subsequently raises intracellular pH and thereby inhibits viral entry into host target cells^530^. Furthermore, ALA could increase intracellular GSH levels and reinforce human host antiviral defense^531^. Therefore, ALA could be considered an effective intervention against SARS-CoV-2 as well as a potent redoxeutical to resolve *m*-Dys-associated clinical manifestations in COVID-19 and PASC^532,533^. A combination of ALA (50 µM) and palmitoyl-ethanolamide (5 µM) could reduce OxS (overproduction of ROS and NO•) and modulate the major inflammatory cytokines (IL-β, IL-6,TNFα, and IL-10) involved in COVID-19 infection^534^. A human RCT from Wuhan, China, showed that ALA therapy (1200 mg/d) for 7 days, could reduce a 30-day all-cause mortality rate (37.5%) in critical COVID-19 patients^535^.

*Coenzyme Q10 (CoQ10)* or ubiquinone is a lipophilic cofactor in the mitochondrial ETC of the OXPHOS system that exerts powerful antioxidant, anti-apoptotic, immuno-modulatory and anti-inflammatory effects in cellular metabolism^536^. CoQ10 is also a potent anti-inflammatory agent that effectively down-regulates cytokines (i.e., TNF-α, IL- 6, CRP) and could optimize viral-disrupted ACE2/RAAS system, by exerting anti-angiotensin II effects and decreasing OxS in COVID-19 patients^537,538^. Excess release of cytotoxic ROS during *m*-Dys leads to OxS, which may hyper-activate platelet function and pose risk of thrombosis in COVID-19 patients. As a potent mitochondrial redox regulator, CoQ10 could prevent thrombotic events in COVID-19 and PASC patients by resolving ROS-induced platelet aggregation^539^. CoQ10 is expressed in all tissues; however, its biosynthesis drops down with ageing and sharply declines during OxS in COVID-19^540^. Thus, CoQ10 as an adjuvant combined with other mitochondrial nutrients could provide potential therapeutic options to resolve hyper-inflammation and reset HMRD in COVID-19 and PASC^541,542^.

*Creatine* could replenish mitochondrial viability and restore cognitive function(s) by down-regulating *toll-like receptors* (TLRs) involved in neuroinflammation and neurodegeneration. The potent antioxidant activity of creatine could also protect mitochondrial DNA from ROS-mediated oxidative damage^543,544^. Orally administered *guanidino-acetic acid* (GAA) could positively affect creatine metabolism, alleviate several aspects of fatigue and improve both physical as well as work capacity in patients with CFS^545^. Human RCTs have suggested that creatine (monohydrate form) could revitalize cellular bioenergetics, neuro-metabolism, and immune function, thereby may provide a multifunctional benefit in recovery of PASC with MS/CFS complications^546,547^.

*Vitamin-B12 (Vit-B12)* also known as cobalamin, is vital for cardiovascular function, as well as for immune regulation and antiviral defense^548,549^. Vit-B12 is also an essential nutrient for ‘skeletomuscular–gut–brain’ axis to maintain skeletal muscle, neuro-cognitive functions, and modulate gut microbiota^550,551^. Vit-B_12_ has ranked among the top four bioactive nutrients for potential management of COVID-19 and PASC^552^. Thus, vit-B_12_ combined with clinical nutrition is a potential adjuvant to reset HMRD in COVID-19 and PASC patients.

### Nutritional reset of oxidative stress (OxS)

Virus-induced OxS with excess levels of ROS i.e., *superoxide anion* (O_2_^•-^), *hydroxyl radical* (^•^OH), *singlet oxygen* (^1^O_2_), and *hydrogen peroxide* (H_2_O_2_), could trigger severe clinical manifestations including hyper-inflammation, tissue damage, thrombosis, and MODS in COVID-19, which may continue in PASC^553,554^. In the body, O_2_^•-^ anions are intended products of redox signaling enzyme cascade and byproducts of several metabolic processes including mitochondrial respiration^555^. Superoxide (O_2_^•-^) anions are scavenged by redox enzyme *superoxide dismutase* (SOD), whereas H_2_O_2_ by *catalase* (CAT), *glutathione* (GSH), *GSH-peroxidase* (GPx), *thioredoxin peroxidase* (Trx), and *peroxiredoxins* (Prdx)^556^. Any decline in redox enzymes may increase free radical generation with subsequent induction of lipid peroxidation, protein oxidation, and DNA/RNA degradation^557–559^.

Serum levels of SOD, CAT, GSH, and GPx are significantly altered in COVID-19 patients^560,561^. The depleted total antioxidant capacity (in blood) of SARS-CoV-2 infected individuals serves as a predictive marker for COVID-19 severity^562^. Both OxS and hyper-inflammatory state during the acute phase of COVID-19, could also predict severity of chronic fatigue, depression, and anxiety symptoms even after 3 to 4 months in the virus-free PASC patients. Based on cluster analysis, a majority of PASC patients show severe abnormalities in SpO_2_, increased OxS and reduced antioxidant indices^563^. Therefore, antioxidant enzymes could be considered an effective nutritional strategy to resolve OxS and reset HMRD in COVID-19 and PASC.

*Superoxide dismutases (SODs)* are metalloenzymes that trigger endogenous antioxidant machinery, the first-line defense against ROS in the body^564^. SOD catalyzes the conversion of *superoxide* (O_2_^•-^) into O_2_ and H_2_O_2_^557^. The H_2_O_2_ is further hydrolyzed to water via CAT and GPX enzymes^565^. Three isoforms of SOD exist in human body: the cytosolic Cu-, Zn-SOD (SOD1), the mitochondrial Mn-SOD (SOD2) and the extracellular Cu-, Zn-SOD (SOD3)^566^. PC-SOD (recombinant human SOD1 covalently coupled to four molecules of lecithin) is a potent superoxide-radical scavenger with a 100-fold increase in protective effects against endothelial cell injuries, compared to unmodified SOD^567,568^. OxS plays a critical role in COVID-19 and PASC; therefore, the therapeutic use of SOD and SOD-mimetics (e.g., *Mangafodipir*) may prove beneficial in PASC recovery^565,569^.

*Catalase (CAT)*, a heme enzyme that catalyzes the decomposition of H_2_O_2_ to water + molecular O_2_, provides a vital cellular antioxidant defense^570^. Excessive production of H_2_O_2_ in mitochondria could damage lipids, proteins, mDNA, resulting in necrosis or apoptosis; where then CAT could protect such cells from H_2_O_2_-induced oxidative injury^571^. Apart from its main substrate H_2_O_2_, the CAT enzyme could also process other oxidative species such as O_2_^•-^, ^•^OH, ^1^O_2_, *hypochlorous acid* (HOCl), NO•, and *peroxynitrite* (ONOO^-^). A number of these free radicals are formed under oxidative ‘eustress’ (good stress) and ‘distress’ (bad stress), where CAT could help regulate the cellular redox-oxidative status^572^. CAT-mediated decomposition of H_2_O_2_ to water minimizes the downstream flow of excessive ROS, which otherwise could trigger OxS and *m*-Dys in COVID-19 and PASC patients. CAT plays a crucial intermediary role in S-protein binding to hACE2 receptors, thereby affects the host susceptibility to SARS-CoV-2 infection^573^. CAT could also regulate cytokine production in leukocytes, protect alveolar cells from oxidative injury, and block SARS-CoV-2 replication^574^.

Solid lipid nanoparticles based on phosphatidylcholine stabilizers, is a functional CAT supplement designed to resist enteric digestion and deliver potent antioxidant activity^575^. Supplemental CAT is shown to alleviate OxS in the GI tract and improve gut microbiota^576^. Lactic acid bacteria, *Lactobacillus casei* BL23, *L. delbrueckii* subsp. *bulgaricus* CRL 864, and *Streptococcus thermophilus* CRL 807, produce both antioxidant enzymes CAT as well as SOD, and provide intrinsic immunomodulatory benefits in the GI tract^577,578^. Programmable probiotics are promising dietary adjuvants to prevent OxS in the gut, curb intestinal inflammation, and resolve certain persistent GI pathologies (e.g., colitis, inflammatory bowel disease, and dysbiosis) in PASC patients^579^.

*Glutathione (GSH*) (γ-l-glutamyl-l-cysteinyl-glycine) is a tripeptide synthesized in the cytosol by two ATP-consuming enzymatic reactions^580^. GSH reaches millimolar levels (1–10 mM) within cells, micromolar levels (10–30 μM) in plasma, and its low redox potential (E^′^_0_ = − 240 mV) makes GSH an ideal cellular redox buffer^581,582^. GSH is commonly found in reduced GSSG form in cytosol, nucleus, mitochondria, and endoplasmic reticulum^583^. The GSSG/GSH redox couple interacts with other antioxidant enzymes to maintain mitochondrial function and cellular redox homeostasis^584^. The GSSG/GSH redox couple plays a vital role in several enzymatic reactions, including the elimination of peroxides by *GSH peroxidases* (GPx), in covalent addition of cysteines to proteins by *glutaredoxin*, and in detoxification of electrophiles by *GSH-S-transferase* (GST)^585–587^. GSH plays the role of ‘master antioxidant’ in tissues; where the high millimolar levels of GSSG in reduced form emphasizes its regulatory role in processes such as detoxification, protein folding, antiviral defense and immune response^588^. Mitochondria are the main source of ROS, generated from the ETC/OXPHOS and any excess release of toxic free radicals could trigger OxS and *m*-Dys^589^. GSH is the main cellular antioxidant to reduce H_2_O_2_ and lipid hydroperoxides (LOOH) catalyzed by GPXs^589,590^. A key enzymatic step in antioxidant clearance with GSH redox cycle is converting H_2_O_2_ to water, which is further catalyzed by GPx. This reduction step occurs via oxidation of GSH to GSSG, and the GSSG is subsequently reduced back to GSH in the body via the enzyme *glutathione reductase* and NADPH^591^.

The SARS-CoV-2-induced FeRD, its ensuing OxS could deplete cellular antioxidant reserves and increase severity of COVID-19 and PASC^27^. Decreased expression of GSH synthesis leads to low free GSH levels, resulting in elevated ROS, immune dysfunction, and increased disease severity in COVID-19 patients^592^. SARS-CoV-2 infection causes GSH deficiency in the body through inhibition of *nuclear factor erythroid 2–related factor 2* (*Nrf2*) pathway, the up-regulator of GSH synthesis^593^. During OxS condition, Nrf2 is transported from cytoplasm into the nucleus by *karyopherins*, where SARS-CoV-2 interferes with transfer process and reduces GSH synthesis^594^. GSH precursors, particularly NAC, are widely used to revert OxS and replenish low GSH levels in pulmonary episodes such as ARDS, bronchitis, or emphysema in COVID-19 and PASC^595^. Furthermore, comorbidities such as hypertension (56.6%), obesity (41.7%), and diabetes (33.8%) are frequently linked to OxS and chronic inflammation in hospitalized COVID-19 patients^596^. In obese patients, OxS is associated with diminished GSH levels and decreased GSH/GSSG ratio^597^. Low GSH levels could also increase viral replication, pro-inflammatory cytokine release, endothelial damage, and immune-thrombosis, which is a hyper-coagulative clinical condition that could exacerbate morbidity and mortality in COVID-19 and PASC^598^. Since *m*-Dys and OxS jointly contribute to both COVID-19 and PASC pathology, nutritional to replenish optimal GSH levels could be a promising strategy to reset HMRD and support patient recovery^582^.

Ferroptosis, the programmed cell death due to iron and lipid dependent peroxidation, is associated with ageusia and anosmia, the early clinical manifestations of COVID-19^197,599,600^. Ferroptosis is regulated by lipid repair enzymes, which also include GSH and GPx4 reducing lipid hydroperoxides (L-OOH) to lipid alcohols (L-OH)^601^. Ferroptosis could cause severe tissue damage and MODS in COVID-19 patients. Administration of liposomal GSH could boost intrinsic GSH levels, enhance GPx4 function and reduce tissue damage from ferroptosis in COVID-19 and PASC^602^.

*N-Acetyl-L-Cysteine (NAC)* is a sulfur-containing AA that breaks disulfide bonds, increases viscosity of mucoproteins and serves as an antioxidant in pulmonary mucous secretions of the respiratory tract^603^. NAC is widely used as a mucolytic agent to improve airway clearance in chronic respiratory diseases. *Glucose 6-phosphate dehydrogenase* (G6PD) deficiency predisposes GSH depletion and increases susceptibility to SARS-CoV-2 infection, and such GSH depletion could be reversed with NAC administration^604^. As a precursor for GSH synthesis, adjuvant therapy with NAC could resolve SARS-CoV-2-induced OxS via GSH release and help restore cellular redox homeostasis during COVID-19 and PASC^605^.

SARS-CoV-2 infects type II pneumocytes and disrupts the cellular Fe-RH with increased ROS release causing severe OxS. Treatment of COVID-19 with NAC (dosage: 600 mg) could provide a prophylactic benefit, and at higher dosage (1200 mg) could serve a therapeutic regimen when administered at the first onset of symptoms^606^. Oral administration of NAC (600 mg every 8 h) to COVID-19 patients (*n* = 19,208) with high-risk comorbidities (i.e., hypertension, dyslipidemia, diabetes, and COPD) is shown to significantly lower mortality rate^607^. NAC intervention (1200–1800 mg/d) markedly improve oxygenation (SpO_2_/FiO_2_) parameters in 10 days, reduce inflammatory markers, and shorten the length of hospitalization in COVID-19 patients^608^. In a retrospective study, COVID-19 patients (*n* = 1083) receiving NAC (1200 mg/d) had a shorter length of hospital stay^609^. In a cross-sectional study, COVID-19 patients (*n* = 164) receiving NAC with standard therapy had an average hospital stay duration of 12 days, a 97% rate of discharge, an average duration of O_2_ therapy for 8 days with limited transfer to ICU, and only one case of fatality^610^. NAC, as a precursor for reduced GSH, demonstrates antioxidant, anti-inflammatory and immunomodulatory effects, which may prove beneficial in modulating any excess inflammatory activation during COVID-19^611,612^. Therefore, nutritional supplementation with NAC could effectively resolve OxS and target pathophysiological pathways involved in SARS-CoV-2 infection and persistent pulmonary fibrotic sequelae in PASC^613^.

*Glutamine* is a precursor for several bioactive molecules in plasma and skeletal muscle, largely utilized for gluconeogenesis in the liver. Glutamine increases cellular GSH levels, improves antioxidant capacity, reduces OxS and inflammation in the body^614^. Severe OxS with elevated blood levels of *high sensitivity-C-reactive protein* (hs-CRP) is a hallmark of hyper-inflammatory state in COVID-19 and PASC^290,553,615^; therefore, proper nutrition rich in antioxidants is critical for recovery of these patients^616^. Glutamine is also a widely used nutritional antioxidant in several hospital ICU-admitted patients with respiratory infections^617^. Glutamine supplementation (10 g/3x daily) for 5 days could reduce serum levels of IL-1β, hs-CRP, TNFα and increase appetite in COVID-19 patients with pulmonary complications^614^.

#### Maillard reaction (MR) and maillard reaction products (MRP)

MR, also known as *Maillard conjugation* or *glycation*, is a non-enzymatic process that forms covalent bonds between the NH_2_ group of AAs and the carbonyl (C = O) group of reduced sugars^618^. MR generates MRPs that include several protein/peptide-saccharide conjugates. MRPs demonstrate enhanced free radical (ROS) scavenging activity and other bio-functional properties; therefore, widely used in the food industry as emulsifiers, antioxidants, antimicrobials, gelling as well as anti-browning agents. MRPs are also effective delivery systems to enhance stability and bioavailability of several dietary compounds.

Dietary MRPs are powerful antioxidants that chelate metal ions, breakdown radical chains/*hydrogen peroxide* (H_2_O_2_), and scavenge ROS^619^. For example, *rutin* (a bioflavonoid found in medicinal herbs and plant-derived foods) interacts with α-AAs (i.e., lysine, isoleucine, histidine, or glutamic acid) to generate phenolic-MRPs. A lysine-based thermal MR process (at 120 °C for 30-min) converts rutin to less-polar ‘quercetin’ with increased ROS-scavenging activity in hepatocytes^620^. *Quercetin*, the rutin-lysine-generated MRP, effectively inhibits free radicals, enhances activity of antioxidant enzymes (i.e., SOD and CAT), initiates Nrf2-dependent pathway, and upregulates phase II detoxifying antioxidant genes (including NQO1, HO-1, GCLG, and GCLM)^621^. Notably, the cellular equilibria between pro-oxidants versus antioxidants (redox homeostasis) regulates the ROS levels and ensuing OxS. The two major transcription factors: i) the NFκB upregulates the pro-oxidant mediators, and ii) the Nrf2 activates the antioxidant responses^622^.

*Quercetin* is considered a major natural bioactive intervention to combat OxS in the ongoing COVID-19 pandemic (4 RCTs on COVID/PASC; https://ClinicalTrials.gov/)^623^. Quercetin affects the expression of 30% of genes that encode viral target proteins in human cells, and potentially interfere with the activities of 85% of SARS-CoV-2 proteins^624^. Quercetin also inhibits *protein disulfide isomerase* (PDI) enzyme involved in platelet-mediated thrombin formation, thus could ameliorate coagulation abnormalities in PASC^625^.

MRPs could enhance the antioxidant potential of several dietary/food systems and play a supportive role in precision nutrition to help reset virus-induced HMRD. MRPs (i.e., gliadin) in the bread crust are shown to induce NF-kB pathway in macrophages and boost antioxidant defense^626^. Early studies have shown that nutritional reconditioning with MRPs could improve antioxidant status of the heart and provide cardioprotective benefits against severe OxS (as in ischemia reperfusion injury)^627^. MR could significantly enhance the antioxidant and other functional properties of *lactoferrin* (LF) by forming covalent complexes with beet pectin^628^. Structural modifications to diol type ginsenosides form MRPs that potentiate *hydroxyl* (OH•) radical-scavenging activity of *Panax ginseng*^629^. MRPs prepared from fermentation of milk proteins by lactic acid bacteria show higher antioxidant activity than the intact milk protein, low intracellular ROS production and sustain reduced-GSH levels in hepatocytes^630^. Milk-based MRPs could protect against oxidative damage and reduce cardiovascular risks^631^. Milk-based MRP consumption could reduce OxS and resolve dysbiosis from virus-induced HMRD^632^. Taken together, in food systems containing phenolic antioxidants (e.g., quercetin) and proteins (e.g., lactoferrin), MR could enhance antioxidant defense and provide an effective delivery/carrier system to develop nutritional reset strategies to resolve OxS-associated impairments in virus-induced HMRD.

### Nutritional reset of virus-hijacked ACE2/RAAS

ACE2 is a key component of the *renin-angiotensin-aldosterone system* (RAAS) that plays a vital role in regulating blood pressure, vasoconstriction, sodium retention, tissue remodeling, pro-inflammatory and pro-fibrotic functions^633,634^. The viral hijack of human ACE2 receptor disrupts RAAS activation, upregulates NF-κB pathway, triggers cytokine storm, hypertension, cell proliferation, inflammation, and fibrosis, where all elicit detrimental effects on every bodily organ during SARS-CoV-2 infection^635^. Nutritional supplementation with l-carnitine could mitigate these pathobiological processes by inhibiting NF-κB and down-regulating NOX1/NOX2, thereby enhance the antioxidant effects of angiotensin II^636^. As an immunomodulator, l-carnitine could decrease proinflammatory cytokines (i.e., TNF-α, IL-6, and IL-1) and help reduce cytokine storm. l-carnitine could also protect against SARS-CoV-2-induced cardiotoxicity resulting from dysregulated ACE2 signaling pathway^637,638^.

*l-Carnitine*, a micronutrient composed of essential AAs (i.e., lysine and methionine), is a cofactor that converts long-chain free fatty acids to acyl-carnitine and transfers these metabolites into the mitochondrial matrix^639^. This hydrophilic AA is widely distributed in CNS, PNS, heart and skeletal muscle, holding >95% of body’s total carnitine^638,640^. l-Carnitine plays a vital role in lipid metabolism and its deficiency could induce feeling of tiredness or general fatigue. Therefore, fatigue could possibly be relieved by restoring serum carnitine levels through supplementation^641^. l-carnitine levels in patients with *chronic fatigue syndrome* (CFS) is 30 to 40% lower than healthy subjects. Oral administration of l-carnitine (3 g/d) with omega-3 fatty acids could increase *carnitine palmitoyl-transferase-I* activity and relieve clinical symptoms of CFS^642^. SARS-CoV-2 infection requires a high basal energy expenditure for immune activation, hyper-inflammation (‘cytokine storm’), anorexia followed by muscle loss, weakness, and fatigue^643^. Circulating autoantibodies may also have a major role in the manifestation of long-term fatigue in PASC patients^644^. Acetyl- l-carnitine supplementation could generate energy from mitochondrial oxidation of fatty acids and help mitigate fatigue in PASC patients^637^.

### Nutritional reset of virus-hijacked NRP1/neuro-cognitive impairment

NRP1 is expressed in olfactory epithelium, astrocytes, and neuronal cells (which lack ACE2 expression) and serve as major CSR for SARS‐CoV‐2 infection of the *central nervous system* (CNS)^645,646^. Viral hijack of NRP1 facilitates CNS invasion of SARS-CoV-2 through the *blood brain barrier* (BBB), and consequential neuro-cognitive symptoms such as anosmia, ageusia, headaches, confusion, delirium, and strokes in early COVID-19^647^. The ensuing pathophysiology involves virus-induced neuronal damage, neuroinflammation, rupture of the BBB, microvasculitis and hypoxia^648^. Neuroinflammation with hypometabolic lesions cause chronic cognitive impairment in COVID patients^649^. Neurological manifestations of COVID-19 and PASC include damage to CNS and PNS, encephalitis, myelitis, myositis, Guillain Barré syndromes, and cognitive impairments^171,650^. These neuro-complications are prevalent among one third of COVID-19 cases, and this clinical condition may persist as chronic symptoms in PASC patients as frequent complaints of brain fog (81%) and fatigue (58%)^651,652^. Nutritional reset of neuro-cognitive dysfunction from viral hijack of NRP1 indeed is of high priority in clinical management of PASC.

*Melatonin* (*N*-acetyl-5-methoxytryptamine) is a derivative of tryptophan, synthesized/secreted by the pineal gland and reaches peak levels in plasma during the night hours^653,654^. This chrono-biotic hormone serves as a photo-periodic switch, influencing the activity of suprachiasmatic nucleus and facilitates human sleep-wake. Melatonin plays multi-functional roles including the regulation of circadian rhythms, immune modulation, oxidative processes, apoptosis, and mitochondrial homeostasis^655^. Melatonin deficiency may lead to CVD with manifestations of hypertension and myocardial ischemia/reperfusion injury, which are prevalent in COVID-1; as well as neuro-cognitive complications such as brain fog, sleep disorders and *myalgic encephalomyelitis/chronic fatigue syndrome* (ME/CFS), which are persistent among PASC^656,657^.

Melatonin with its antioxidant, anti-inflammatory, immune-modulatory, and anti-apoptotic effects, is considered a potential therapeutic against COVID-19 and PASC. As a powerful antioxidant, melatonin scavenges toxic free radicals (ROS/RNS) and prevents oxidative damage of DNA through activation of DNA repairing pathways^658^. This neuro-hormone could also elevate levels of antioxidant enzymes (i.e., SOD, CAT, GSH and GPx), and inhibit detrimental effects of NLRP3 inflammasome^659^. Melatonin could resolve hyper-inflammatory conditions in COVID-19 and PASC by down-regulating proinflammatory cytokines (i.e., TNF-α, IL-1β, IL-6, and IL-8) and increasing anti-inflammatory cytokines such as IL-10^660,661^. The anti-inflammatory activity of melatonin may also involve *Sirtuin-1* induction, suppression of NF-κB activation, and stimulation of Nrf2^662^. Furthermore, the anti-apoptotic and cyto-protective effects of melatonin could stabilize mitochondrial membrane and help reset *m*-Dys^663^.

Based on several human RCTs, melatonin is considered an effective intervention to resolve delirium, ameliorate respiratory stress (i.e., ARDS), and restore circadian balance in COVID and PASC patients^664–666^. Furthermore, as a cyto-protectant, melatonin helps alleviate several COVID-19 comorbidities, including T2DM, metabolic syndromes, and both ischemic as well as non-ischemic CVD^657^. In an open-label RCT (*n* = 96), oral administration of melatonin tablets (3 mg/d, 1 h before sleep, for 7 days) with standard treatment, substantially improved sleep quality and blood O_2_ saturation parameters in hospitalized COVID-19 patients^667^. In another RCT (*n* = 80), *prolonged-release melatonin* (PRM 2-mg) therapy showed a significant improvement in sleep hours and reduction in delirium episodes among hospitalized insomniac COVID-19 patients^668^. Melatonin activates two of the G-protein-coupled receptors: MT1 that regulates vigilance states of *rapid eye movement* (REMS), and MT2 that controls non-REMS. In accordance with the circadian rhythm, melatonin release into blood at night could improve sleep quality of insomnia patients through activation of MT1 and MT2 receptors^669^. Finally, melatonin could help alleviate neurological complications such as brain fog, ME/CFS, anxiety, and sleep disorders; resolve ALI/ARDS with related vessel permeability issues. Based on its multi-functionality, high safety profile, melatonin could be considered a promising adjuvant support for nutritional reset of neuro-COVID in PASC patients^171,665,670^.

### Nutritional reset of virus-hijacked serine proteases

In healthy lungs, *type II transmembrane serine proteases* (i.e., TTSPs: TMPRSS2, CTSL, HAT) play a major role in cellular regeneration, repair, and homeostasis^671,672^. Furthermore, anti-proteases (i.e., *secretory leukocyte protease inhibitor*, SLPI) are important for proteolytic inhibition and host defense^673^. A functional balance between proteases and anti-proteases is vital to ensure respiratory homeostasis. Any imbalance towards increased protease expression and activity may lead to overt inflammation and trigger chronic lung disorders such as COPD and emphysema^674,675^. Furthermore, respiratory serine proteases that belong to the TTSP family, increase host susceptibility to SARS-CoV infection-2^55^. Anti-proteases, such as SLPI, inhibit the activity of serine proteases and block viral entry into host target cells^70^. Therefore, the protease/antiprotease balance not only is critical for respiratory homeostasis but also serves as a powerful determinant of SARS-CoV-2 pathogenesis. Nutritional antioxidants could stimulate anti-protease secretion, decrease protease activity and protect epithelial cells against viral infection^676^. Thus, antioxidants serve as regulators of the protease/antiprotease balance that could effectively combat viral infection(s) including COVID-19.

Respiratory epithelium is constantly prone to inhalation insults from excess release of toxic free radicals, ROS/RNS, and peroxides, all cumulatively exert OxS^677^. Nrf2, the innate transcription factor, regulates synthesis and activity of several antioxidant enzymes to help resolve OxS, and prevent tissue damage in lungs^678^. Nrf2 also regulates TMPRSS2, protease/antiprotease balance and protects the respiratory milieu against viral infections^676,679^. Thus, bioactive nutrients that activate Nrf2 could reduce persistent OxS as well as help in functional optimization of viral-hijacked serine proteases and reset HMRD in PASC patients^70^.

*Flavan-3-ols* found in green tea, such as *epigallcatechin-3-gallate* (EGCG), is shown to induce SLPI secretion, reduce TMPRSS2 secretion, and decrease viral replication^680^. Antioxidant supplementation with flavan-3-ols may serve as a possible nutraceutical therapy to protect against lung disease in the context of a viral infection.

*Sulforaphane* (SFN), a sulfur-containing isothiocyanate compound naturally found in cruciferous vegetables (i.e., cauliflower, broccoli, brussels sprouts, and cabbage) could enhance Nrf2 activity. SFN could induce cellular antioxidants such as *heme oxygenase* (HO)-1 and *NADPH quinone oxidoreductase 1* (NQO1) activities, thereby inhibit proinflammatory cytokine release^681^. SFN supplementation could also increase SLPI secretion, regulate TMPRSS2 expression and plays an important role in modulation of protease/antiprotease balance^676,679^.

### Nutritional reset of immune impairment

SARS-CoV-2 infection triggers immune response, a regular host defense strategy to restrain viral entry and constrain disease progression. However, when immune system exhausts and/or compromised, the viral infection become aggressive in vulnerable hosts and evolves into a severe pathophysiological state with hyper-inflammation, extensive tissue damage, and MODS^682^. Such hyper-inflammatory state could lead to loss of appetite, altered intestinal absorption, impaired gut permeability, and malnutrition^683,684^. Malnutrition in turn could aggravate inflammatory pathways, compromise the immune system, onset dysbiosis, increase risks of new microbial infection(s) as well as reactivate latent pathogens^685^. Therefore, nutritional resolution of immune dysfunction is an important aspect of recovery from severe inflammation, malnutrition, and sarcopenia during clinical rehabilitation of COVID-19 and PASC.

*Vitamin D3 (Vit-D)* is a lipid-soluble seco-steroid that exists in two forms as D_2_ (ergocalciferol) derived mainly from plant sources and D_3_ (cholecalciferol), which is present in higher animals^686^. Both endogenous and exogenous forms of vit-D are inactive and require two successive hydroxylation steps by cytochrome P450 (CYP) enzymes to form fully active vit-D. In humans, vit-D3 is produced by the skin with conversion of 7-dehydro-cholesterol to cholecalciferol via exposure to sunlight^687^. Circulatory vit-D is initially transported to liver by vit-D binding protein^688^. Vit-D is mainly known to regulate calcium/phosphate homeostasis and bone metabolism. However, vit-D is also vital for several biological pathways including modulation of innate and adaptive immune responses^689^. Immuno-modulatory role of vit-D could be categorized into three essential functions: (i) physical barrier, (ii) natural cellular immunity, and (iii) adaptive immunity^690^. Vit-D could activate the release of *cathelicidin* and *defensins* to inhibit viral replication; furthermore, could down-regulate release of proinflammatory Th1 cytokines (i.e., TNF-α and IFN-γ) and stimulate macrophages to generate anti-inflammatory cytokines to minimize the risk of ARDS in COVID-19^691^. Vit-D could also inhibit NF-κB activation to down-regulate proinflammatory cytokine synthesis and other key activators of cell-mediated immunity^692^. *Vit-D receptor* (VDR) is expressed on antigen-presenting cells, T and B lymphocytes that also synthesize active vit-D metabolite. Vit-D can modulate both innate and adaptive immune responses, and vit-D deficiency is linked to increased autoimmunity as well as increased susceptibility to microbial infections. Vit-D deficiency is prevalent among patients with autoimmune disorders^689^. Vit-D is a negative regulator for expression of renin and interacts with the RAAS/ ACE/ACE-2 signaling axis, therefore could affect SARS-CoV-2 infection process as well as host CV/circulatory function^693^.

In COVID-19 patients, vit-D deficiency was reported in 41.7% cases, vit-D insufficiency in 46.0%, and the remaining 12.3% of cases with normal vit-D levels. The odds of severe COVID-19 outcomes increase by 38.1 and 5.6 times for vit-D-deficiency and -insufficiency patients, respectively, for each standard deviation decrease in serum 25(OH)D^694^. Low vit-D levels are associated with elevated inflammatory cytokines with increased risk of pneumonia and viral upper respiratory tract infections. Vit-D deficiency is also associated with an increase in thrombotic episodes, frequently reported in COVID-19^695^. Severe cases of COVID-19 demonstrate 64% more vit-D deficiency than mild cases, while vit-D insufficiency could significantly increase hospitalization and CFR^696^. Vit-D deficiency is a risk factor for unregulated cytokine storm and hyper-inflammation in COVID-19 patients^697^. Also, COVID-19 survivors and PASC patients show lower 25(OH)D levels compared to matched-patients without PASC^698^. Vit-D deficiency refers to serum levels of *25-hydroxyvitamin D*, 25(OH)D, <20 ng/mL (50 nmol/L)^699^.

SARS-CoV-2 pneumonitis could rapidly incapacitate the lung and lead to severe ALI/ARDS, including death in some patients. Vit-D deficiency and/or failure to activate the vit-D receptor could trigger a cytotoxic response in stellate cells of the lung and aggravate respiratory complications in COVID-19 and PASC^396^. Vit-D could ameliorate pulmonary inflammation and facilitate repair of epithelial layers, damaged organs with inherent anti-fibrotic properties and help resolve inflammation-induced pathologies, such as fibrosis^690,700,701^. CVD sequelae such as cardiomyopathy, arrhythmias, thrombotic complications, and cardiogenic shock are prominent features of COVID-19 and PASC pathologies^702^. Vit-D could activate VDR function, regulate calcium flux, reset optimal myocardial contractility, and help reduce risks of myocardial infarction in COVID-19 and PASC patients^703^.

In Spain, a population-based cohort study of 4.6 million people supplemented with cholecalciferol or calcifediol, when achieved serum 25(OH)D levels >30 ng/mL, showed a reduction of about half the risk of SARS-CoV-2 infection, severe COVID-19, or COVID-19 mortality than those not treated^704^. An observational study at the U.S. Veterans Affairs healthcare facilities, patients *(n* = 4599) when adjusted to 25(OH)D levels from 15 to 60 ng/mL, showed a decrease in probability of COVID-19-related hospitalizations from 24.1 to 18.7%, and mortality rates from 10.4 to 5.7%^704^. There is mounting evidence that optimal 25(OH)D levels are associated with reduced risk of COVID-19.

Reactivation of latent *Epstein–Barr virus* (EBV) is an emerging risk factor with adverse outcomes for both COVID-19 and PASC^269,705^. Vit-D supplementation (20,000 IU/week over 96 weeks) could significantly reduce humoral immune responses against latent EBV antigen in relapsing-remitting multiple sclerosis^706^. Although more evidence is needed on therapeutic benefits of vit-D in COVID-19 and PASC, the multifunctional role of this vitamin on immune system is evident. Vit-D deficiency is cost-effective, safe, and readily available supplement strategy for COVID-19 and PASC management^707^. Therefore, individuals at higher risk of vit-D deficiency during COVID-19 pandemic should consider taking vit-D supplements to reset the circulating 25(OH)D levels to optimum (75–125nmol/L) and avoid and/or recover from COVID-19 and PASC.

*Omega-3 Polyunsaturated Fatty Acids (Omega-3 or n-3 PUFAs)*, *eicosapentaenoic acid* (EPA), *docosapentaenoic acid* (DPA), and *docosahexaenoic acid* (DHA), are broad-spectrum anti-inflammatory compounds that modulate several pathways of inflammation including leukocyte chemotaxis, adhesion molecule expression, and leukocyte-endothelial adhesive interactions, inflammatory cytokine synthesis and T cell reactivity^708^. As potent antioxidants, omega-3 PUFAs upregulate Nrf2, *mitogen-activated protein kinase* (MAPK) phosphatases, GSH and HO-1 genes. Metabolites of omega-3 PUFAs are vital for the synthesis of several inflammatory mediators including *prostaglandins* (PG), *leukotrienes* (LT), *thromboxanes* (TX), *protectins*, and *resolvins*^709^. Omega-3 PUFAs modulate both innate and acquired immune systems through activation of macrophages, neutrophils, T-cells, B-cells, dendritic cells, NK cells, mast cells, basophils, and eosinophils. As an integral part of the cellular membrane, omega-3 PUFAs regulate membrane fluidity and complex assembly in lipid rafts^710^. PUFAs could help alleviate mitochondrial ROS production in T cells to combat SARS-CoV-2 infection^21^. Virus-induced HMRD of host lipid metabolism may persist as chronic inflammatory condition in PASC patients. *Palmitoylethanolamide* (PEA), a lipid-derived *peroxisome proliferator-activated receptor-α* (PPAR-α), is shown to dismantle lipid droplets via β-oxidation and restore innate cellular defenses^711^.

Omega-3 PUFAs may interact at different stages of SARS-CoV-2 infection, particularly during viral entry and replication phases where persistence of viral antigens may lead to sustained inflammatory state in PASC patients^277,712,713^. Omega-3 PUFAs, particularly EPA, are potential remedials to reduce pro-inflammatory cytokines, alter the HPA axis, modulate neurotransmission via lipid rafts, and alleviate neuro-cognitive complications in PASC patients^714^. Furthermore, omega-3 PUFAs and their metabolites (i.e., specialized *pro-resolvin* mediators), could effectively ameliorate uncontrolled inflammatory responses, reduce OxS, mitigate coagulopathy, and restore tissue homeostasis^715^. Besides antioxidant and anti-inflammatory activities, omega-3 PUFAs could regulate platelet homeostasis and lower the risk of thrombosis, which indicates its potential use in COVID-19 and PASC management.

COVID-19 patients show significantly low *Omega-3 Index* (O3I = 4.15%) compared to healthy subjects (O3I = 7.84%). Lower O3I is associated with an increased risk of developing severe COVID-19 with mechanical ventilation and high CFR^716,717^. In a retrospective clinical study (*n* = 80), oral supplementation with omega-3 PUFAs has significantly lowered proinflammatory procalcitonin and IL-6 levels and reduced prothrombin time in ICU/hospitalized COVID-19 patients^718^. Therefore, the nutritional status of omega-3 PUFAs is particularly important for the overall immune response, tissue inflammation and repair, which may be an effective nutritional strategy for PASC recovery.

*Vitamin-C (Vit-C)* (ascorbic acid) is a water-soluble, essential nutrient (cannot be synthesized by the human body), important for antioxidant activity and immune-modulation^719^. Vit-C regulates NF-kB release and attenuates pro-inflammatory cytokines production^720^. In a meta-analysis of 7 RCTs and 7 retrospective studies (*n* = 751 patients), vit-C supplementation showed a significant alleviation in inflammatory response by increasing ferritin levels and lymphocyte counts in COVID-19^721^. Another meta-analysis of 19 RCTs showed a reduced in-hospital mortality rate in vit-C supplemented COVID-19 patients (24.1%) compared to non-supplemented group (33.9%)^722^. In a retrospective study from UAE (*n* = 63), oral supplementation of COVID-19 patients yielded no difference in vit-C levels across BMI categories; however, a significant correlation was noted between vit-C levels and SARS-CoV-2 clearance rate among the obese patient group^723^. Extended fecal viral RNA shedding suggests that SARS-CoV-2 infection in the GI tract could be prolonged in a subset of COVID-19 and PASC patients^724^. The potential viral clearance activity of vit-C supplementation could be a promising adjuvant combo with other ‘reset’ nutritional strategies to treat certain PASC patients.

### Nutritional reset of gut dysbiosis

SARS-CoV-2 infection alters gut microflora composition and function, which leads to intestinal barrier dysfunction and immune activation^725^. Epithelial tight junction is a critical intestinal barrier, and its disruption could leak toxic substances from the gut into blood circulation and cause systemic injury. The maintenance of intestinal epithelial tight junctions is closely related to energy homeostasis and mitochondrial function^397^. Such virus-induced dysbiosis triggers cytokine release with NF-κB-mediated hyper-inflammatory response, and the ensuing immune dysregulation could worsen the clinical outcomes of COVID-19^240^. Gut microbiota plays a multi-functional role in the GI tract, including energy extraction from the diet, immune modulation, synthesis of vitamins and *short-chain fat acids* (SCFAs)^726^. A complex equilibrium exists among prebiotics (i.e., fructo-oligosaccharides), probiotics (i.e., LAB), and postbiotics (i.e., bacteriocins, SCFAs), with the involvement of several networks between gut microflora and other organ systems through different axes (i.e., Gut-Lung, Gut-Liver, Gut-Brain axes) that affect a plethora of pathways in health and disease^244,727^.

Gut dysbiosis could persist for at least 6 months in COVID-19 patients after hospital discharge, and this chronic inflammatory condition may onset a wide range of neurological and neuropsychiatric symptoms in PASC patients^244^. Accordingly, several gut metabolic dysfunctions could contribute to long-term neuro-cognitive impairments in PASC, including: (i) perturbed ‘gut-brain’ axis due to loss of SCFA producing intestinal flora, which may cause neuropsychiatric disorders^728^; (ii) cytokine storm-induced immune-metabolic reprogramming, which could elevate ‘kynurenine:tryptophan’ ratio and trigger chronic depression syndrome^729^; and iii) ACE2 activation in the gut could alter l-DOPA production and neurotransmitter synthesis, thereby inflict neurological complications including CFS^730^.

*Probiotics/lactic acid bacteria (LAB)* regulate cytokine secretion and affect both nonspecific as well as specific immune responses. Bacteriocins produced by LAB are antimicrobial compounds known to inhibit adhesion and invasion of microbial pathogens in the GI epithelia^731^. Probiotics could block SARS-CoV-2 proliferation in host cells, via potential immuno-modulation, and inhibit NLRP3 inflammasome activation^732^.

Oral probiotic therapy of hospitalized COVID-19 patients (*n* = 70) with *Streptococcus thermophilus*, *L. acidophilus*, *L. helveticus*, *L. paracasei*, *L. plantarum*, *L. brevis*, *B. lactis*, and *B. lactis*, experienced remission of diarrhea and other symptoms within 72 h. These probiotics show a significant ameliorating effect and reduce the risk of developing respiratory failure by almost eight times in SARS-CoV-2 infected individuals^733^. Probiotic therapy of COVID-19 patients with a combination of nine different lactobacilli strains (as daily dose), showed significant alterations in gut microbiota, partial restoration of pulmonary dysfunction as well as intestinal dysbiosis. Probiotic therapy also reduced inflammatory markers such as TNF-α, IL-1β, IL-4, and IL-12^734^. Co-administration of *L. rhamnosus* EH8 with mycelia could potentially inhibit inflammatory cytokine release induced by SARS-CoV-2 *membrane* (M) glycoprotein^735^. A human RCT with COVID-19 patients (*n* = 300) treated with *L. plantarum* and *Pediococcus acidilactici* for 30-days, showed total remission of 53.1% in the probiotic-treated compared to 28.1% in the placebo groups, respectively. Probiotic therapy showed significant reduction in pulmonary infiltrates as well as in lowering the nasopharyngeal viral load, shortened the duration of GI and non-GI symptoms, decreased the D-dimer levels, and boosted the synthesis of specific IgM and IgG responses against SARS-CoV-2^736^.

Co-administration of *L. plantarum* GUANKE (LPG) strain with COVID-vaccine has been suggested to act as an adjuvant, inducing specific and nonspecific immune response(s), thereby extending protection against SARS-CoV-2^737^. A 3-month administration of probiotic strain *Loigolactobacillus coryniformis* K8 on SARS-CoV-2 (mRNA) vaccine-induced immune responses in elderly population (*n* = 200) was evaluated. All participants completed mRNA vaccination, while the intervention started ten days after the first dose. The IgG levels in were significantly higher in the treated group; however, at ages >85, probiotic administration increased IgA antibody levels^738^. Preclinical studies have shown that probiotic therapy confers specific immune responses in inflammatory cytokine expression, prevent cell apoptosis, and induce immunological memory against COVID-19^739^. Thus, re-balancing of healthy gut microbiota through probiotics, prebiotics, and immune nutrients, could help reduce inflammation, promote anti-inflammatory mechanisms, and reset a functional ‘gut-brain’ axis for optimal recovery of COVID-19 and PASC patients.

### Nutritional reset of virus-induced metabolic disorders

HMRD in tandem with chronic metabolic syndromes could result in more severe outcomes of COVID-19 and subsequently extends into PASC^740^. A meta-analysis of 120 studies (*n* = 125,446 patients) reported that most prevalent comorbidities for SARS-CoV-2 infection include: hypertension (32%), obesity (25%), T2DM (18%), and CVD (16%)^741^. COVID-19 in conjunction with diabetes and obesity (both characterized by severe insulin resistance) has severe clinical consequences^742,743^. Therefore, restriction of dietary lipid and sugar intake could potentially benefit COVID-19 and PASC patients with T2DM, obesity or other metabolic syndromes.

#### Virus-induced metabolic disorder—‘new onset’ diabetes

T2DM is a progressive metabolic disorder due to insulin resistance with underlying chronic inflammation as well as endothelial and β-cell dysfunction^744^. SARS-CoV-2 infection could trigger hyper-inflammation, exacerbate insulin resistance, worsen endothelial dysfunction and lead to new-onset diabetes^745^. COVID-19-induced aberrant glycol-metabolic dysregulation could persist even after COVID-19 recovery. In a cohort of hospitalized COVID-19 patients (*n* = 551) about 46% of cases showed long-term hyper-glycemia (who were normo-glycemic prior to infection)^746^. Therefore, not only patients with metabolic and endocrine dysfunction are predisposed to risks of severe COVID-19; but also, SARS-CoV-2 infected normal population could potentially develop ‘new-onset’ diabetes or aggravation of pre-existing metabolic syndromes^747^. The hyperglycemia in non-diabetic COVID‐19 patients could result from impaired pancreatic islet function as well as viral inflammation-induced insulin resistance and abnormal β cell activation^748,749^. Oral administration of *liposome-embedded SOD* (L-SOD) could ameliorate oxidative damage and inflammatory responses via inhibition of *myeloperoxidase* (MPO) and pro-inflammatory cytokines, as well as protect gut barrier function by promoting the expression of the tight junction proteins *occludin* and *zonula occluden* (ZO)-1 in the colon^750^. Furthermore, L-SOD could also reduce OxS to intestinal barrier, thereby ameliorating the vicious circle between hyperglycemia and the oxidative damage^751^. Therefore, targeting the intestinal barrier with dietary bioactive L-SOD could be a promising glucose-lowering approach to reset HMRD-induced new onset diabetes in PASC patients.

#### Virus-induced metabolic disorder—obesity

*Body mass index* (BMI) strongly correlates with immune signatures that predict severity of COVID-19 and PASC^752^. Obesity is recognized as a high‐risk factor in COVID‐19 patients, and high-fat diet promotes ACE2 expression on adipocytes^753^. The lipid-rich adipocytes facilitate lipid raft formation on cell membranes to support viral entry, as well as provide building blocks to assemble viral capsules^754^. Viral-mediated adipocyte infection (cellular entry, invasion, and propagation) processes could inflict severe adipose tissue dysfunction and insulin resistance^754,755^. Interestingly, SARS‐CoV‐2 has been detected in the adipose tissue of overweight males but not in females. Inhibition of lipase‐mediated breakdown of body fat could effectively block viral propagation in adipocytes^756^. Thus, cellular metabolic state and overall nutrition status are key determinants in the pathobiology of COVID-19 and PASC^21,747^.

### **Nutritional reset of HMRD in complementary and integrative health (CIH) practices**

Demographic distribution of SARS-CoV-2 infection and severity of COVID-19 disease spectrum vary around the world. Viral susceptibility and infectious outbreak in a regional population depends on several geo-genomic factors including local dietary habits, public health practices (including traditional/herbal medicines), environmental as well as socio-economic strata, and prevalence of nutrient (vitamin and mineral) deficiencies. Ready access, minimal side effects with low risk of developing drug resistance, makes *Complementary and Integrative Health (CIH)* practices an ideal adjunct therapeutic strategy to combine with nutrient-based remedial to reset virus-induced HMRD in the global combat of PASC.

*Traditional Chinese Medicine* (TCM) has played a significant role in combat against COVID-19 and PASC in China. Several herbal formulas have been shown to be efficacious such as *Jinhua Qinggan* (JHQG) granules, *Lianhua Qingwen* (LHQW) capsules, *Xuanfeibaidu* (XFBD) granules, *Huashibaidu* (HSBD) and Xuebijing (XBJ)^757^. A bedside-to-bench study in Taiwan (*n* = 12) reported that a novel TCM formula, *Taiwan Chingguan Yihau* (NRICM101), could disrupt progression of SARS-CoV-2 infection through antiviral and anti-inflammatory activities, thereby provide both preventive and therapeutic benefits to combat COVID-19^758^. A prospective cohort has reported that TCM could improve pulmonary inflammation and help in early recovery of PASC patients^759^. *Bufei Huoxue* capsule is shown to resolve hyper-inflammatory response, coagulation abnormalities, and myocardial damage in PASC patients^760^. Acupuncture has been suggested to alleviate many of the clinical symptoms of PASC, including headaches, myalgia, and abdominal pain. A meta-analysis found auricular acupuncture effective in relieving anxiety and depression in COVID-19 patients^761^. Accordingly, stimulation of the *Interferon Point* (located on tragus/ear helix) is shown to improve innate immune defense and accelerate remission in PASC^762^. A human clinical study in Hubei, China (*n* = 84) reported that auricular point pressure involving seed (*Vaccaria segetalis*) placement could relieve insomnia (improve sleep) and reduce situational anxiety in PASC patients^763^. Electro-acupuncture may reduce expression of proinflammatory cytokines and modulate immunity through neuro-regulation^764^. Acupoint stimulation therapy is shown to improve palpitations, dyspnea, cognitive impairment, anxiety, depression, and other symptoms in PASC patients^765^. Nutritional reset of HMRD in combination with TCM could provide a cost-effective remedial strategy to combat PASC, especially in Asia.

#### Phytotherapy

Several phytochemicals have demonstrated activity against the SARS-CoV-2 through mechanisms such as viral entry inhibition, inhibition of replication enzymes, and virus release blockade^766^. Plant-derived natural *non-nucleoside analog inhibitors* (NNAIs) effective against SARS-CoV-2 *RNA-dependent RNA polymerase* complex (*nsp7/nsp8/nsp12*) were reported^767^. Also, phytochemicals could specifically inhibit in silico, viral protein *nsp5*-encoded main protease (M^pro^), the autocleavage enzyme critical for COVID-19 pathogenesis^768^. Phytochemicals from medicinal herbs with antiviral activity include hesperidin, apigenin, luteolin, seselin, 6-gingerol, humulene epoxide, quercetin, kaempferol, curcumin, and *epigallocatechin-3-gallate* (EGCG) have been reported to inhibit multiple molecular targets of SARS-CoV-2 viral replication in silico^769^. For neuro-PASC, the therapeutic potential of 31 phytochemicals (derived from 19 medicinal herbs) to resolve neuro-cognitive impairments such as anxiety, depression*, mixed anxiety-depressive* (MAD) syndromes, and irreversible dementia has been reported^770^. Polysaccharides, terpenoids, flavonoids, alkaloids, glycosides, and lactones are plant-derived immunomodulators also considered as potential remedials against viral infections^771^.

*Ayurvedic Rasayana therapy* is traditionally practiced in India for its immunomodulatory and adaptogenic properties, thus used as a therapeutic adjuvant for COVID-19 and PASC recovery. Amongst several others, *Withania somnifera* (Ashwagandha), *Tinospora cordifolia* (Guduchi) and *Asparagus racemosus* (Shatavari) play a major role in Rasayana therapy^772^. Advanced computational technology has provided rapid and cost-effective techniques to screen phytochemicals from AYUSH (*Ayurveda, Yoga, Naturopathy, Unani, Siddha, Sowa-Rigpa*, and *Homeopathy*) for PASC management. *Basti* and *Rasayana* treatments showed potent immunomodulatory effects in regulating pro-inflammatory cytokines, IgG, and T cell function; accordingly, proposed for rejuvenation therapy to combat PASC^773^. Mucormycosis is an opportunistic angio-invasive fungal infection associated with PASC^774^. In a prospective human RCT (*n* = 77), Ayurvedic therapy as an adjunct to conventional medical treatments showed significant improvement across the entire spectrum of mucormycosis in PASC patients^775^.

Patients discharged from intensive care pose higher risk of functional loss or undernutrition, even after 6-months post-COVID infection. Malnutrition and loss of muscle strength should be considered in the clinical assessment of these PASC patients^776^. Yoga is a psycho-somatic approach to enhances innate immunity and mental health, so it can be used as complementary therapy with nutritional reset of PASC^777^.

*Chiropractic* could provide an adjuvant modality to complement the nutritional reset of virus-induced HMRD in PASC. Skeleto-muscular complaints, fatigue, insomnia, and cognitive impairments are prominent clinical manifestations of PASC, which are also common features in *fibromyalgia*, a disorder of the *autonomic nervous system* (ANS)^778^. Chiropractic *spinal manipulation therapy* (SMT) could regulate ANS at peripheral level and reach the CNS. The vagal parasympathetic stimulation by SMT, could then release neurotrophins (*brain-derived neurotrophic factor*, BNDF and *nerve growth factor*, NGF) to help resolve depression and related neuro-cognitive impairments^779^. Other multi-modal chiropractic treatments such as massage and intermittent motorized cervical traction could relieve soft-tissues, inter-vertebral joints, and stretch the core musculatures to facilitate rehabilitation of FM patients^780^. *Endogenous paired associative stimulation* (ePAS), a neuro-modulatory intervention, could increase muscle power and resolve total neuro-muscular fatigue^781^. Furthermore, chiropractic SMT could also resolve migraine and cervicogenic headaches, which are prevalent among PASC patients. A 17-month RCT in Norwegian patients (*n* = 104) showed a significant improvement in migraine duration and headache index with SMT compared to control group^782^. Also, chiropractic modalities with SMT, *soft tissue therapy* (STT), stretching and mobilizations may also provide safe adjunct treatment for treatment of GI disorders^783^, in combination with nutrient-based reset of HMD/R in PASC. A combination of chiropractic modalities with nutritional reset strategies needs an in-depth evaluation, especially in resolving neuro-cognitive, skeleto-muscular, and GI impairments in PASC patients.

## Conclusions/future directives

This narrative review elaborates critical steps involved in the emergence, gradual progression, and chronic manifestation of PASC or long-COVID, the ongoing virus-induced global ‘new-onset’ human metabolic syndrome. The pathophysiology of PASC is described right from the initiation of the ‘novel’ SARS-CoV-2 infection by primordial hijacking of host cellular metabolic machinery, subsequent progression of virus-induced *human metabolic reprogramming/dysregulation* (HMRD) in a susceptible host, subjecting the patient through a crucial tri-phasic symptomatic clinical onset of COVID-19 (lasting from 3–4 weeks), and ultimate transition of a survivor into PASC or long-COVID, a virus-free state, lingering with earlier and/or new onset disease manifestations resulting from pre-acquired HMRD (lasting for weeks to months).

Besides hijacking of specific host cellular factors (i.e., ACE2, NRP1, furin, TMPRSS2, CTSL), this ‘novel’ RNA (29.9-kb) virus encodes 14 ORFs that could also partake in >4,780 unique high-confidence virus–host protein–protein interactions in the human body. Such extensive viral ORF/protein interactions with host-specific cellular targets could trigger severe HMRD, a rewiring of sugar-, AA-, FA-, and nucleotide-metabolism(s); as well as hypoxia (’Warburg’ effect) with *m*-Dys (altered ATP synthesis), immune impairment, and FeRD in an infected individual. Accordingly, the SARS‐CoV‐2 genome and its products potentially modulate HMRD at transcription, translation, and *post-translational modification* (PTM) levels of human metabolism. A plethora of PASC clinical symptoms and related metabolic impairments indicate an involvement of various pathophysiological mechanisms originating from virus-induced HMRD. Thus, PASC is not a simple disease, but a complex disorder of multi-organ systems resulting from virus-induced HMRD; henceforth, should be categorized as a ‘new onset’ human metabolic syndrome.

The virus-free, dysfunctional metabolic state of PASC could manifest with >200 different and overlapping clinical symptoms involving multiple organ/systems. Such dysfunctional metabolic sequelae are cumulative outcomes of virus-induced HMRD involving about 10 divergent pathophysiological mechanisms, comprising of both virus-derived virulence factors as well as a multitude of extreme innate host responses. Each of these underlying etiologies amplified by HMRD would require specific system-targeted remedial(s) to achieve healthy recovery of PASC patients. Therefore, precision nutrition protocols to resolve systemic impairments and ‘reset’ the virus-induced HMRD is the most relevant and effective strategy to combat PASC. An ideal nutritional remedy should demonstrate human RCT proven health benefits with optimal ADME (*Administration, Distribution, Metabolism, Excretion*) profile and deliver functional advantage to reset HMRD and help total recovery of PASC patients. *The term ‘RESET’ refers to nutritional or dietary-based remedials or interventions that help resolve dysfunctional cellular pathways from virus-induced HMRD and recalibrate host metabolism to optimal function and homeostasis*. We have described a few evidence-based, human RCT tested, bioactive nutritional interventions for resetting of virus-induced HMRD in PASC through precision nutrition (*refer* Table 1).

FeRD, the cellular iron redox imbalance triggered by HMRD plays a detrimental role during the cytokine storm in COVID-19 and its clinical aftermath^27,48,49^. Furthermore, hyperferritinemia with oxidized iron levels modulate several pathways of coagulation cascade and cause severe thromboembolism in PASC^99^. Innate Fe-Redox regulators such as *lactoferrin* (LF), *heme oxygenase* (HO)-1, *erythropoietin* (EPO), and *hepcidin* (HEP) serve as front-line innate barriers against free radical (ROS/RNS) damage and hyper-immune responses during COVID-19 and PASC^27^. Nutrient remedials to reset FeRD with ferroptosis inhibitors (i.e., ferrostatin-1, vit-E) could be effective in post-recovery strategy for PASC^94,441^. Virus-induced HMRD in tandem with hypoxia and *m-*Dys affects several cellular metabolic pathways in COVID-19 survivors (after viral clearance), which could ultimately evoke severe PASC with metabolic impairments including new onset T2DM, cardiovascular disease, chronic fatigue syndrome, brain fog, and blood clotting issues^21,143^. l-tryptophan is an essential AA, vital for biosynthesis of serotonin (5-HT), melatonin and co-factor NAD^+^ through its downstream metabolic pathways^425^. l-tryptophan metabolism is the most prominent pathway that undergoes HMRD during SARS-CoV-2 infection. Nutrient availability is the major regulator of life and reproduction, and a complex cell signaling network monitors the cellular energy metabolism, especially the mitochondrial ATP synthesis and NAD^+^/NADH ratio, are major sensors of metabolic state. Re-activation of TCA cycle, mitochondrial metabolism, OXPHOS, and ATP synthesis could reverse and reset virus-induced HMRD. Nutritional revitalization of *m*-Dys should include specific bioactives and cofactors (i.e., l-tryptophan, NAD^+^, CoQ10, α-lipoic acid, creatine, vit-B12), which are essential for mitochondrial-ETC and provide potential benefits to resolve hyper-inflammation, exercise intolerance, CFS and reset HMRD in PASC^541,542^.

Administration of antioxidant enzymes could be considered an effective nutritional strategy to resolve OxS and reset Fe-RH in COVID-19 and PASC. Since *m*-Dys and OxS jointly contribute to both COVID-19 and PASC pathology, nutritional reset of virus-induced HMRD with NAC, glutamine, GSH, and antioxidant enzymes (i.e., SOD, catalase) could potentially resolve OxS and persistent pulmonary fibrotic sequelae in PASC patients^582^. Bioactive nutrients such as flavan-3-ols that activate Nrf2 could also reduce chronic OxS, help restore activity of viral-hijacked serine proteases and reset HMRD in PASC patients. The SARS-CoV-2 infection could reach the brainstem and inflict cerebral lesions as long-term sequelae with several neuro-cognitive dysfunctions in PASC patients. Nutritional reset of HMRD with l-tryptophan and 5-HT could help resolve neuro-psychiatric disorders (resulting from viral hijack of NRP1), is of high priority in clinical management of neuro-PASC. Melatonin could help alleviate neurological complications such as brain fog, ME/CFS, anxiety, and sleep disorders; resolve ALI/ARDS with related vessel permeability issues of neuro-COVID. Considering a high safety profile, melatonin could provide a promising adjuvant support for nutritional reset of HMRD-inflicted neuro-COVID in PASC patients^171,665,670^. The viral hijack of human ACE2 receptor disrupts RAAS activation, upregulates NF-κB pathway and the ensuing cytokine storm, hypertension, cell proliferation, inflammation, and fibrosis elicits detrimental effects on every bodily organ, the CV system, in particular^635^. Nutritional supplementation with l-carnitine could mitigate these pathophysiological processes by inhibiting NF-κB and down-regulating NOX1/NOX2, thereby enhancing the antioxidant effects of angiotensin II^636^. Also, the vit-D supplementation could activate VDR function, regulate Ca^2+^ flux, reset cardiac muscle contractility, and help reduce risks of myocardial infarction in COVID-19 and PASC patients. While the omega-3 PUFAs could regulate platelet homeostasis and lower the risk of thrombosis, oral supplementation of l-arginine is a promising nutritional remedy to reset HMRD-associated CV disorders and immune dysfunction in PASC patients. In PASC, the HMRD-amplified immune disruption could cause tissue damage and aggravate GI disorders such as loss of appetite, leaky gut with severe malnutrition. Such immune exhaustion could onset dysbiosis, increase risk of new microbial infection(s) and reactivate latent viral pathogens with new onset of ME/CFS in PASC patients. Gut dysbiosis may persist for months in COVID-19 patients after hospital discharge, and the virus-induced HMRD could also trigger neuro-cognitive symptoms in PASC patients. Re-balancing the GI tract with effective probiotics, prebiotics, and immune nutrients, could help resolve inflammation, promote anti-inflammatory activity, and reset a functional ‘gut-brain’ axis for recovery of PASC patients.

### Epilogue—the final note

Life is a genetically programmed, intricately organized, chemical processing thermo-dynamic system, that self-sustains by breaking chemical bonds (catabolism) to release and capture free energy to form complex macro-molecules (anabolism) for specific structure-function (metabolism) in a lipid-based envelope, known as the cell. Viruses infect almost every species and are probably the most abundant biological entities on the planet Earth. Recently emerged coronavirus, the SARS-CoV-2, is a 29.9-kb RNA virus that possess a unique genomic ability to reprogram a mega-sized 3.1-Mb human DNA and its cellular metabolic machinery to prime, alter, and redirect host macro-molecules for its infection, replication, and propagation. The 14 viral ORFs interact with a few thousands of human metabolites in a specific manner for its intra-cellular invasion, replication, and transmission, which results in HMRD in favor of the viral pathogen. Genomic and meta-genomic data have revealed that co-evolution between viral and cellular genomes involves frequent horizontal gene transfer and the occasional co-option of novel functions over evolutionary time^784^, the virus-induced HMRD with PASC, is one such prime example. Therefore, the nutraceutical/food supplement industry should take a serious note that formulating a RESET composition is not a simple blend of food compounds or nutritional ingredients into a dosage form. A deeper meta-genomic insight to assure 3-D structure/function of nutrient molecules, priming/activation of nutrients with co-factors, compositional stoichiometry, optimal milieu (pH, redox, and ionic strengths), molecular interplay between bioactives (synergism/antagonism), target delivery, ADME/Safety/Toxicity profiles, and most importantly the bio-functional activity, are vital prerequisites in the development of nutrient-based remedials to RESET the virus-induced HMRD in long-COVID, the ‘new onset’ global metabolic syndrome. This precision nutrition-based dietary rehabilitation of PASC patients should be developed as an affordable, interventional regimen for divergent socio-economic populations worldwide for effective public health management of any future emergence of virus-induced ‘new onset’ human metabolic syndromes.

### Reporting summary

Further information on research design is available in the Nature Research Reporting Summary linked to this article.

### Supplementary information

Reporting summary

## Data Availability

The data that support the findings of this study are available from the corresponding author upon reasonable request.
