# Author Correction: Precision nutrition to reset virus-induced human metabolic reprogramming and dysregulation (HMRD) in long-COVID

**DOI:** 10.1038/s41538-024-00267-w

**Published:** 2024-05-06

**Authors:** A. Satyanarayan Naidu, Chin-Kun Wang, Pingfan Rao, Fabrizio Mancini, Roger A. Clemens, Aman Wirakartakusumah, Hui-Fang Chiu, Chi-Hua Yen, Sebastiano Porretta, Issac Mathai, Sreus A. G. Naidu

**Affiliations:** 1 Global Nutrition Healthcare Council (GNHC) Mission-COVID, Yorba Linda, CA USA; 2 N-terminus Research Laboratory, 232659 Via del Rio, Yorba Linda, CA 92887 USA; 3 School of Nutrition, Chung Shan Medical University, 110, Section 1, Jianguo North Road, Taichung, 40201 Taiwan; 4 College of Food and Bioengineering, Fujian Polytechnic Normal University, No.1, Campus New Village, Longjiang Street, Fuqing City, Fujian China; 5 President-Emeritus, Parker University, 2540 Walnut Hill Lane, Dallas, TX 75229 USA; 6 University of Southern California, Alfred E. Mann School of Pharmacy/D. K. Kim International Center for Regulatory & Quality Sciences, 1540 Alcazar St.,CHP 140, Los Angeles, CA90089 USA; 7 International Union of Food Science and Technology (IUFoST), Guelph, ON Canada; 8 IPMI International Business School Jakarta; South East Asian Food and Agriculture Science and Technology, IPB University, Bogor, Indonesia; 9 Department of Chinese Medicine, Taichung Hospital, Ministry of Health &Well-being, Taichung, Taiwan; 10 Department of Family and Community Medicine, Chung Shan Medical University Hospital; School of Medicine, Chung Shan Medical University, Taichung, Taiwan; 11 President, Italian Association of Food Technology (AITA), Milan, Italy; 12 Experimental Station for the Food Preserving Industry, Department of Consumer Science, Viale Tanara 31/a, I-43121 Parma, Italy; 13 Soukya International Holistic Health Center, Whitefield, Bengaluru, India

**Keywords:** Nutrition, Mechanisms of disease

Correction to: *npj Science of Food* 10.1038/s41538-024-00261-2, published online 30 March 2024

In this article, the affiliation details ‘World Federation of Chiropractic, Toronto, ON, Canada.’ was incorrectly added for Fabrizio Mancini. The original article has been corrected.

